# Design, Synthesis, and In Vitro Enzymatic Evaluation of Novel Flavone Derivatives as Dual COX-2/5-LOX Inhibitors Supported by Molecular Docking and ADMET Analysis

**DOI:** 10.3390/cimb48030243

**Published:** 2026-02-25

**Authors:** Elmehdi Fraj, Amine Elbouzidi, Haytham Bouammali, Hanane Jaouani, Chaymae Bourhou, Mohamed Addi, Susu M. Zughaier, Allal Challioui, Rachid Touzani, Boufelja Bouammali

**Affiliations:** 1 Laboratory of applied Chemistry and Environment, Faculty of Sciences, Mohammed First University, Oujda 60000, Morocco; elmehdi.fraj@ump.ac.ma (E.F.); haytham.bouammali@ump.ac.ma (H.B.); hanane.jaouani.d23@ump.ac.ma (H.J.); bourhou.chaymae96@gmail.com (C.B.); a.challioui@ump.ac.ma (A.C.); r.touzani@ump.ac.ma (R.T.); 2 Laboratory for Agricultural Production Improvement, Biotechnology, and Environment (LAPABE), Faculty of Sciences, Mohammed First University, Oujda 60000, Morocco; amine.elbouzidi@ump.ac.ma (A.E.); m.addi@ump.ac.ma (M.A.); 3 College of Medicine, QU Health, Qatar University, Doha P.O. Box 2713, Qatar; szughaier@qu.edu.qa

**Keywords:** flavone–tetrazole hybrids, anti-inflammatory activities, COX-2, 5-LOX molecular docking, ADMET

## Abstract

The development of new anti-inflammatory agents with improved safety and efficacy remains a major therapeutic challenge, particularly in light of the adverse effects associated with conventional nonsteroidal anti-inflammatory drugs. In this study, a series of new flavone derivatives were synthesized and evaluated for their inhibitory activities against Cyclooxygenase-1 (COX-1), Cyclooxygenase-2 (COX-2), and 5-Lipooxygenase (5-LOX) through combined in vitro and in silico approaches. Biological screening demonstrated that several derivatives exhibited moderate to strong inhibitory activity across the three enzymes, with IC_50_ values ranging from 35.67 ± 2.92 to 1137.44 ± 371.05µM. Among these, compounds **5a** and **5b** emerged as the most promising dual COX-2/5-LOX inhibitors, displaying potent activity toward both targets while maintaining limited COX-1 inhibition, as reflected by their favorable selectivity indices (SI = 2.09 and 5.21, respectively). Molecular docking studies supported the experimental findings, revealing favorable binding affinities of compounds **5a** and **5b** within the COX-2 active site (PDB: 1CX2), while the flavone–tetrazole hybrid **6b** exhibited the highest binding affinity toward the 5-LOX active site (PDB: 6N2W), consistent with its notable inhibitory activity. In silico ADME and toxicity predictions further suggested that the selected derivatives (**4a-b**, **5a-b**, and **6a-b**) possess acceptable physiochemical properties, low predicted toxicity, and favorable drug-likeness. Overall, this study identifies flavone-based scaffolds as a promising early-stage lead for the development of dual COX-2/5-LOX inhibitors and provides a rational basis for the design of safer anti-inflammatory agents.

## 1. Introduction

Inflammation plays a central role in the development and progression of many chronic diseases, including articular, cardiovascular, oncological, and neurological disorders [1,2,3]. During inflammation, multiple mediators are generated, among which eicosanoids, such as prostaglandins and leukotrienes, derived from arachidonic acid metabolism, are of particular importance [4,5]. Arachidonic acid, released from membrane phospholipids through the action of phospholipase A_2_, acts as an essential precursor in the regulation of inflammatory responses [6]. Once released, it undergoes metabolism through two major enzymatic pathways [1,7]. The first is the cyclooxygenase (COX) pathway, which gives rise to prostaglandins, prostacyclins, and thromboxanes. These bioactive lipids participate in key physiological and pathological processes, including pain, fever, vasodilation, and platelet aggregation. The second is the lipoxygenase (LOX) pathway, which leads to the formation of leukotrienes, hepoxilins, and lipoxins. These metabolites play critical roles in modulating both inflammatory and immune responses. Collectively, these interrelated pathways ensure the fine-tuned regulation of inflammation, immune function, and cellular homeostasis. Consequently, COX and LOX enzymes have emerged as major therapeutic targets in the development of anti-inflammatory agents [1,8]. Their inhibition, whether selective or combined, represents a key strategy to mitigate inflammation and its associated detrimental effects, such as pain and oxidative stress.

**Figure 1:**
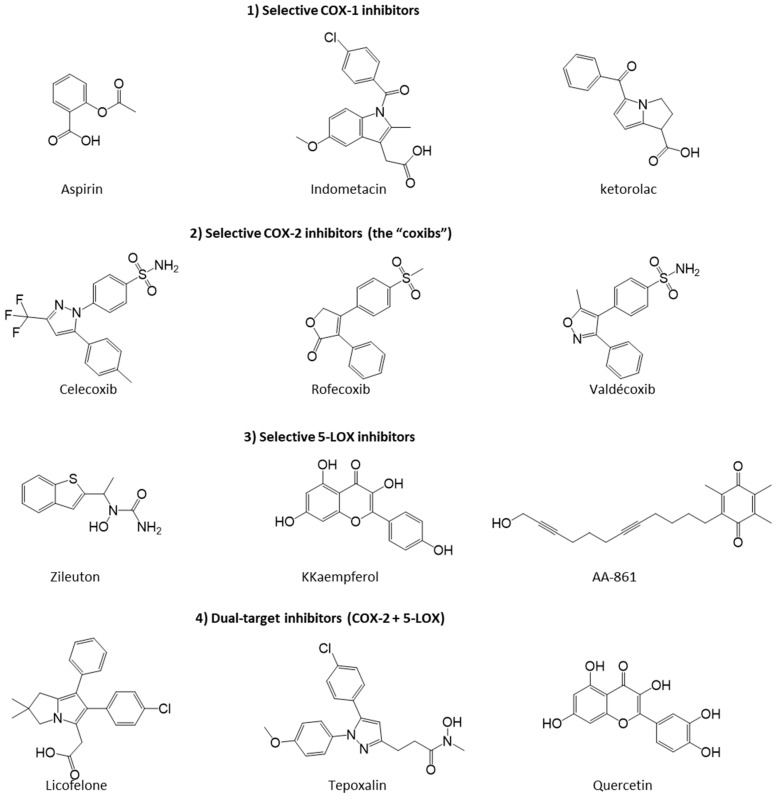
Structures of well-known NSAIDs used as a COX and/or LOX inhibitors.

Nonsteroidal anti-inflammatory drugs (NSAIDs) remain the most widely used anti-inflammatory agents, acting primarily by inhibiting COX enzymes (COX-1 and COX-2) and, in some cases 5-LOX [1,9]. COX inhibitors block the conversion of arachidonic acid to prostaglandin H_2_ (PGH_2_), reducing the synthesis of mediators responsible for pain, fever, and vasodilation [10,11]. Common examples include ibuprofen, naproxen, diclofenac, aspirin, celecoxib, etoricoxib, and rofecoxib, along with several natural compounds such as alkaloids, terpenoids, flavonoids, saponins, and fatty acids (Figure 1) [12,13,14].

The lipoxygenase (LOX) enzyme family consists of several isoforms (5-LOX, 8-LOX, 12-LOX, and 15-LOX) classified by the position of oxygenation arachidonic acid [15,16]. Among them, 5-LOX plays a central role in leukotriene biosynthesis, acting as a key mediator in both acute and chronic inflammatory processes [17,18]. LOX inhibitors impede the formation of arachidonic acid hydroperoxides, thereby limiting leukotriene production. Representative examples include zileuton, montelukast, and pranlukast, as well as natural compounds such as flavonoids (quercetin, kaempferol), phenolic acids, and coumarins (Figure 1) [19,20]. These inhibitors show considerable therapeutic potential for treating asthma, allergies, atherosclerosis, and certain cancers, owing to their capacity to suppress leukotriene synthesis and reduce oxidative stress [4].

The simultaneous inhibition of COX and LOX represents a next-generation strategy to enhance efficacy while reducing NSAIDs-related side effects [21,22]. Dual inhibitors suppress both prostaglandin and leukotriene formation, thereby preventing the metabolic shunting of arachidonic acid toward the uninhibited pathway [23]. Among these multi-target agents, licofelone and tepoxalin (Figure 1) have been investigated for their balanced inhibition of COX-1, COX-2, and 5-LOX isoforms. Although these compounds demonstrated dual inhibitory activity, their clinical development was limited by efficacy or safety concerns. Several naturally occurring flavonoids, such as quercetin, apigenin, and luteolin, also demonstrate dual inhibitory properties [24]. Furthermore, recent studies have focused on novel heterocyclic derivatives (including pyrazoles, thiazoles, oxadiazoles, and tetrazoles) for their potential as dual COX/LOX inhibitors combining both anti-inflammatory and antioxidant activities [2].

Dual COX/LOX inhibitors offer several notable therapeutic benefits: they reduce the risk of gastric ulceration typically linked to conventional NSAIDs, prevent the accumulation of pro-inflammatory leukotrienes, and provide a more balanced pharmacological profile, resulting in improved tolerance and fewer gastrointestinal and cardiovascular side effects [25]. Nevertheless, current anti-inflammatory drugs are still associated with numerous adverse effects, including gastrointestinal complications, cardiovascular risks, and renal toxicity [26]. Hence, the search for novel, safer, and more effective anti-inflammatory agents capable of concurrently inhibiting COX and LOX pathways has become a key research priority.

**Scheme 1:**
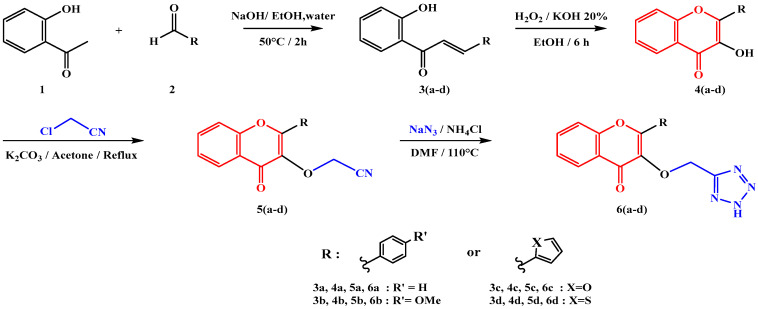
Synthesis of flavone–tetrazole hybrids **6**.

In line with our research on novel anti-inflammatory agents [27], we report the synthesis of a new series of flavone–tetrazole hybrids **6** (Scheme 1) and evaluated their activity against COX-2 and 5-LOX. The tetrazole moiety contributes pharmacophoric activity, and acts as a bioisostere of carboxylic acids and amides, enhancing their lipophilicity, bioavailability and metabolic stability [28].

Finally, molecular docking studies were performed as a qualitative tool to elucidate ligand–enzyme interactions and to identify the structural features underlying the observed anti-inflammatory effects.

## 2. Materials and Methods

### 2.1. Reagents and Instruments

All reagents employed in this study were obtained from Sigma-Aldrich via local distributors and were of analytical grade. They were used without any additional purification.

Reaction progress was monitored by thin-layer chromatography (TLC) using silica gel 60 F254 plates (0.25 mm). Purification by column chromatography was carried out on silica gel 60 (230–400 mesh). Melting points were measured in open capillary tubes using an Electrothermal 9100 apparatus and are reported without correction. Infrared (IR) spectra were recorded on a Jasco-4700-ATR FT-IR spectrophotometer, and absorption bands are given in cm^-1^ (ν). ^1^H and ^13^C-NMR spectra were acquired on spectrometers operating at 300, 400, or 600 MHz. Chemical shifts (δ) are reported in parts per million (ppm) relative to tetramethylsilane (TMS), using CDCl_3_ or DMSO-d_6_ as solvents. Coupling constants (J) are expressed in hertz (Hz), and signal multiplicities are denoted as s (singlet), d (doublet), t (triplet), q (quartet), quint (quintet), m (multiplet), dd (doublet of doublets), ddd (doublet of doublets of doublets), and td (triplet of doublets). High-resolution mass spectra (HRMS, ESI) were recorded on an Ultimate 3000-Exactive Plus Thermo mass spectrometer (CNRST platform, Rabat, Morocco), with *m*/*z* values reported in atomic mass units.

### 2.2. Synthesis Procedure

The synthesis and characterization of compounds **3a-c**, **4a-c**, **5a-c**, and **6a-c** are described in detail in our previous publication (the NMR and HRMS spectra of these compounds are provided in the Supplementary Material, Figures S27–S62) [27].

#### 2.2.1. Synthesis of Chalcones (**3d**)

A mixture of 2′-hydroxyacetophenone (**1**) (1 g, 7.35 mmol, 1 equiv) and NaOH (735 mg, 18.4 mmol, 2.5 equiv) was dissolved in 25 mL of a 40% ethanol–water solution and stirred at 0 °C for 15 min. Then, 2-thiophenecarboxaldehyde (**2**) (824 mg, 7.35 mmol, 1 equiv) was added dropwise, and the reaction mixture was maintained at 60–65 °C for 2 h. After cooling in an ice bath, the medium was acidified with 12 mL of 1 N HCl. The resulting precipitate was collected by filtration, washed with water and cold methanol, and recrystallized from methanol to afford the desired product **3d**.

Yellow solid (1.354 g, 80%), mp: 94.1 °C. IR (ν; cm^-1^): 3086.51 ν(O-H); 1630.52 ν(C=O); 1554.34 ν(C=C); 1481 and 1434 ν(C=C, aromatic); 1204 ν(C2-C3); 1146 ν(C1′-S-C4′); 835 and 761 δ(C H, para and meta). ^1^H-NMR (CDCl_3_, 300 MHz): 12.86 (1H, s, OH); 8.05 (1H, d, J = 15 Hz, H-1); 7.88 (1H, dd, J = 8.1 and 1.5 Hz, H-9); 7.51 (1H, dt, J = 8.5 and J = 1.5 Hz, H 7); 7.50 (1H, dd, J = 6 and 1.2 Hz, H-4′); 7.43 (1H, d, J = 15 Hz, H-2); 7.40 (1H, dd, J = 5.1 and 1.2 Hz, H-2′); 7.11 (1H, dd, J = 5.1 and 3.6 Hz, H-3′); 7.02 (1H, dd, J = 8.4 and 1.2 Hz, H-6); 6.95 (1H, dt, J = 8.5 and 1.5 Hz, H-8). ^13^C-NMR (CDCl_3_, 75 MHz): 193.15 (C3); 163.56 (C-5); 140.17 (C-1′); 137.88 (C-1); 136.37 (C-9); 132.78 (C-3′); 129.57 (C-7); 129.54 (C-2); 128.55 (C-6); 119.94 (C-4); 118.88 (C-8); 118.82 (C-5′); 118.61 (C-4′). HRMS (ESI) for C_13_H_10_O_2_S [M+H]+ (*m*/*z*): calculated 231.04351, found 231.04550.

#### 2.2.2. Synthesis of 3-Hydroxy-2-(thiophen-2-yl)-4H-chromen-4-one (**4d**)

In an appropriate reaction flask, chalcone **3d** (500 mg, 2.17 mmol, 1 equiv) was suspended in 18 mL of ethanol, followed by the addition of 2.2 mL of 20% aqueous KOH. The mixture was stirred at room temperature for 15 min. Subsequently, 4 mL of 30% hydrogen peroxide was added dropwise over 15 min, and the reaction was allowed to proceed under continuous stirring at ambient temperature for 6 h. After completion, the mixture was cooled in an ice bath, and 8 mL of 5 N HCl was added, resulting in the formation of a precipitate. The solid product (4d) was collected by filtration, washed with cold water, and purified by recrystallization from a chloroform–methanol mixture (9:1).

Bright green solid (320 mg, 60%), mp: 202 °C. IR (ν; cm^-1^): 3127 ν(O-H); 1593 ν(C=O); 1556 ν(C=C); 1476 and 1427 ν(C=C, skeletal vibrations of the aromatic ring); 1244 ν(C2-O-C9); 1118 ν(C1′-S-C4′); 855 and 754 δ(Ar-H, para and meta). ^1^H-NMR (DMSO- d_6_, 600 MHz): 10.23 (1H, s, OH); 8.06 (1H, dd, J = 7.9 and 1.6 Hz, H-5); 7.93 (1H, dd, J = 3.8 and 1.2 Hz, H-4′); 7.87 (1H, dd, J = 5.0 and 1.2 Hz, H-2′); 7.75 (1H, ddd, J = 8.6, 7.0 and 1.7 Hz, H-7); 7.68 (1H, dd, J = 8.5 and 1.03 Hz, H-8); 7.42 (1H, ddd, J = 8.0, 6.9 and 1.1 Hz, H-6); 7.26 (1H, dd, J = 5.0 and 3.8 Hz, H 3′). ^13^C-NMR (DMSO- d_6_, 151 MHz): 172.57 (C-4); 154.69 (C-9); 143.74(C-3); 137.20 (C-7); 134.20 (C 4′); 132.87 (C-1′); 131.56 (C-2′); 128.92 (C-2); 128.36 (C-3′); 125.36 (C-6); 125.13 (C 5); 122.32 (C-10); 118.68 (C-8). HRMS (ESI) for C_13_H_8_O_3_S [M+H]+ (*m/z*): calculated 245.02724, found 245.02545.

#### 2.2.3. Synthesis of 2-((4-oxo-2-(Thiophen-2-yl)-4H-chromen-3-yl)oxy) Acetonitrile (**5d**)

**Figure 2:**
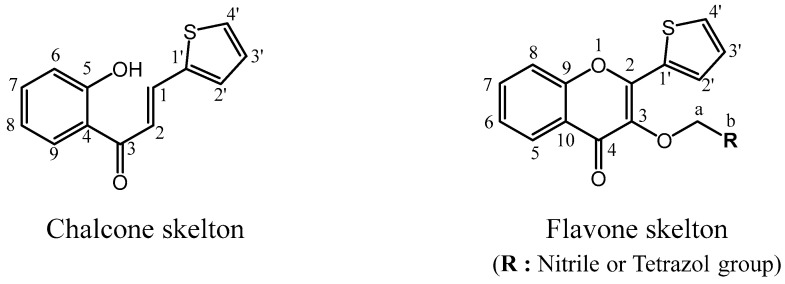
Numberings used to name chalcones and flavones.

A solution of flavonol derivative **4d** (250 mg, 1.02 mmol, 1 equiv) was prepared in 10 mL of anhydrous acetone. To this solution, potassium carbonate (353 mg, 2.55 mmol, 2.5 equiv) was added, and the reaction mixture was stirred at room temperature for 15 min. Subsequently, a solution of 2-chloroacetonitrile (77 mg, 1.02 mmol, 1 equiv) in 3 mL of dry acetone was added dropwise. The mixture was then heated under reflux for 24 h. Once the reaction was complete, the reaction was cooled in an ice bath, and 15 mL of cold water was added to induce precipitation. The resulting solid (compound **5d**) was collected by filtration, washed thoroughly with water, and purified by recrystallization from absolute ethanol. The numbering system used for the chalcones and flavones is depicted in Figure 2.

White solid (200 mg, 69%), mp: 152 °C. IR (ν; cm^-1^): 3106 ν(C-H, aromatic); 2985 ν(C-H, aliphatic); 2184 ν(CN, nitrile); 1625 ν(C=O); 1602 ν(C=C); 1466 and 1420 ν(C=C, skeletal vibrations of the aromatic ring); 1239 ν(C_2_-O-C_9_); 1133 ν(C1′-S-C4′); 853 and 802 δ(Ar-H, para and meta). ^1^H-NMR (DMSO- d6, 400 MHz): 8.13–8.10 (2H, m, H-2′ and H-4′); 8.09 (1H, d, J = 8.3 Hz, H-5); 7.89 (1H, td, J = 7.7 and 1.7 Hz, H-7); 7.80 (1H, d, J = 8.3 Hz, H-8); 7.54 (1H, t, J = 7.4 Hz, H-6); 7.38 (1H, t, J = 4.5 Hz, H 3′); 5.35 (2H, s, Ha). ^13^C-NMR (DMSO- d6, 100 MHz): 172.88 (C4); 154.83 (C-9); 152.02 (C-2); 135.59 (C 3); 135.02 (C-7); 134.15 (C-4′); 131.65 (C-2′); 130.76 (C-1′); 128.66 (C-3′); 125.94 (C 6); 125.37 (C-5); 123.68 (C 10); 118.79 (C-8); 116.73 (Cb); 56.93 (Ca). HRMS (ESI) for C_15_H_9_NO_3_S [M+H]+ (*m*/*z*): calculated 284.03814, found 284.03607.

#### 2.2.4. Synthesis of 3-((2H-Tetrazol-5-yl)methoxy)-2-(thiophen-2-yl)-4H-chromen-4-one (**6d**)

In an appropriate reaction flask, 3-O-cyanomethylflavone (**5d**) (150 mg, 0.53 mmol, 1 equiv) was dissolved in 5 mL of N,N-dimethylformamide (DMF). Sodium azide (NaN_3_) (87 mg, 1.33 mmol, 2.5 equiv) and ammonium chloride (NH_4_Cl) (71 mg, 1.33 mmol, 2.5 equiv) were added to this solution. The mixture was then heated under stirring at 110 °C for 24 h. After the reaction mixture was allowed to cool to room temperature, it was filtered to remove any insoluble materials, and the filtrate was concentrated under reduced pressure. The obtained residue was transferred into 5 mL of water, and 1 N HCl was added dropwise until complete precipitation occurred. The resulting solid was isolated by filtration, washed three times with 10 mL portions of water, and purified by silica gel column chromatography in two successive steps: first, elution with dichloromethane to eliminate impurities, followed by methanol elution to afford the target compound **6d**.

Brown solid (67 mg, 66.7%), mp: 181 °C. IR (ν; cm^-1^): 3018 ν(N-H); 2738 ν(C-H, aromatic); 1598 ν(C=O), 1551 ν(C=C); 1417 and 1402 ν(C=C, skeletal vibrations of the aromatic ring); 1239 ν(C2-O-C9); 1132 ν(C1′-S-C4′); 828 and 760 δ(Ar-H, para and meta). ^1^H-NMR (DMSO- d6, 400 MHz): 8.13 (1H, dd, J = 7.8 and 1.8 Hz, H-5); 8.04 (1H, dd, J = 3.9 and 1.5 Hz, H-4′); 7.98 (1H, dd, J = 5.07 and 1.41 Hz, H-2′); 7.87 (1H, ddd, J = 8.7, 6.9 and 1.7 Hz, H-7); 7.80 (1H, d, J = 8.0 Hz, H 8); 7.54 (1H, td, J = 7.9 and 1.3 Hz, H-6); 7.30 (1H, dd, J = 5.1 and 3.7 Hz, H-3′); 5.66 (2H, s, Ha). ^13^C-NMR (DMSO- d6, 100 MHz): 173.22 (C4); 154.83 (C-9); 151.82 (C-3 and Cb); 136.38 (C1′); 134.83 (C-7); 133.66 (C-4′); 131.18 (C-2′); 130.97 (C-2); 128.47 (C-3′); 125.77 (C 6); 125.45 (C 5); 123.92 (C-10); 118.75 (C-8); 62.09 (Ca). HRMS (ESI) for C_15_H_10_N_4_O_3_S [M+H]+ (*m/z*): calculated 327.05518, found 327.05333.

### 2.3. Evaluation of In Vitro Anti-Inflammatory Activity

#### 2.3.1. In Vitro Cyclooxygenase (COX-1/COX-2) Inhibition Assays

The inhibitory effects of compounds **4-6** on cyclooxygenase isoforms (COX-1 and COX-2) were evaluated using a prostaglandin biosynthesis assay, adapted with minor modifications from the protocol described by Futaki [29,30]. Test solutions were prepared in 1% (*v*/*v*) DMSO, resulting in a final concentration of 500 µg/mL. Each reaction mixture consisted of 1 unit of COX-1 or COX-2 in 0.1 M Tris-HCl buffer (pH 7.5), supplemented with 1 µM hemin and 2 mM phenol. The mixtures were pre-incubated with either the test compound or vehicle for 2 min at 37 °C, after which the reaction was initiated by adding arachidonic acid to a final concentration of 51.4 µM (Sigma, St. Louis, MO, USA) and allowed to proceed for 2 min at 37 °C. The reactions were terminated by extraction with 400 µL of an n-hexane/ethyl acetate mixture (2:1, *v*/*v*), followed by centrifugation at 2000 rpm for 1 min. The upper organic layer was collected and evaporated to dryness, and PGE_2_ levels were measured using a radioimmunoassay via liquid scintillation counting. COX-1 (EC 1.14.99.1; ram seminal vesicles) and COX-2 (sheep placenta, ~70% purity) were obtained from Cayman Chemical (Ann Arbor, MI, USA), and indomethacin served as the reference inhibitor. IC_50_ values (95% CI) were calculated by fitting dose–response data to a four-parameter logistic (4PL) model.

#### 2.3.2. In Vitro 5-Lipoxygenase (5-LOX) Inhibition Assays

The anti-inflammatory activity of compounds **4-6** was evaluated by measuring their inhibitory effects on the 5-lipoxygenase (5-LOX) enzyme. The assay was performed with modifications based on the procedure described by Ghuman [31], including adjusted concentration ranges for both the test compounds and the standard. Compounds **4-6** were prepared in DMSO, yielding a final concentration of 1% (*v*/*v*) in the assay. Enzymatic activity was monitored spectrophotometrically (Jenway 6300) at 560 nm through the formation of the Fe^3+^/xylenol orange complex.

Commercially available soybean 5-lipoxygenase (Glycine max) was used as a model enzyme due to its stability and structural similarity to human 5-LOX. The enzyme was pre-incubated at 25 °C for 5 min with either compounds **4-6** or the reference inhibitor quercetin. Linoleic acid was then added to a final concentration of 140 μM in 50 mM Tris-HCl buffer (pH 7.4), and the reaction was allowed to proceed for 20 min at 25 °C in the dark. The reaction was stopped by adding 100 μL of FOX reagent, consisting of a 9:1 (*v*/*v*) methanol/water solution containing 30 mM sulfuric acid, 100 μM xylenol orange, and 100 μM ferrous sulfate (Fe^2+^). Negative controls (buffer and enzyme only) and blanks (substrate added after FOX reagent) were included to ensure reproducibility. After a further 30 min incubation at 25 °C, absorbance was recorded at 560 nm to assess the inhibition of hydrogen peroxide production, serving as a measure of lipoxygenase activity. Percent inhibition was then calculated using the appropriate formula:

$$\mathrm{Inhibition}\;\mathrm{percentage}\left(\%\right)=\left[\frac{\left(A_{\mathrm{control}}-A_{\mathrm{sample}}\right)}{\left(A_{\mathrm{ccontrol}}-A_{\mathrm{blank}}\right)}\right]\times 100$$

$$IC_{50}=\mathrm{antilog}\left[\log\left(A_{\min}\right)+\frac{50-R_{\min}}{R_{\max}-R_{\min}}\times \left(\log\left(A_{\max}\right)-\log\left(A_{\min}\right)\right)\right]$$

where A_control_, A_blank_ and A_Sample_ represent the absorbances of the control well, blank and test sample respectively [32].

All measurements were performed in triplicate, and the results are expressed as mean ± standard deviation (SD). Statistical analysis was carried out using GraphPad Prism (version 8.0.1), and IC_50_ values were obtained by nonlinear regression.

### 2.4. Molecular Docking

All docking simulations were performed using the Schrödinger Maestro software suite (Maestro 13.4, Schrödinger Release 2022-4).

**Protein preparation**: The X-ray crystal structure of cyclooxygenase-2 and 5-Lipooxygenase was retrieved from the Protein Data Bank (https://www.rcsb.org) in PDB format (PDB= 1EQG for COX-1 [33]; 1CX2 for COX-2 [34]; and PDB= 6N2W for 5-LOX [35]). Using Maestro’s Protein Preparation workflow, the water molecules and co-crystallized ligand were removed, hydrogen atoms and missing residues were added, and the structure was subjected to energy minimization with the OPLS4 force field. During this minimization, a root-mean-square deviation (RMSD) restraint of 0.3 Å was applied.

**Ligands preparation**: The synthesized compounds (**4-6**) were initially drawn using ChemDraw Professional 16.0, saved as SDF files, and then imported into Maestro 13.4 for further processing. The Maestro LigPrep module was used to carry out both geometric optimization and energy minimization, while Epik generated ionized and tautomeric forms within a pH range of 7 ± 2. All remaining settings were maintained at their default values.

**Docking Procedure**: The active site of the target protein (PDB ID: 1EQG, 1CX2, and 6N2W) was defined using the Receptor Grid Generation tool in Maestro, where a cubic grid box with 20 Å dimensions along each axis was centered at the active site (1EQG: x = 26.77, y = 34.06, z = 200.17; 1CX2: x = 24.42, y = 21.82, z = 16.24; 6N2W: x = 35.91, y = 65.43, z = 38.84). Docking simulations were then performed with GLIDE (Grid-Based Ligand Docking with Energies) to estimate the binding affinities (kcal/mol), producing binding poses that suggest favorable ligand–protein interactions. Both 2D and 3D visualizations of these interactions were carried out in Discovery Studio Visualizer (Version 2020).

**Docking Validation**: The molecular docking protocol was validated using the native co-crystallized ligands (IBP for COX-1, S58 for COX-2, and NDGA for 5-LOX). Each ligand was removed from its protein structure and redocked into the corresponding binding site, and the resulting poses were then compared to the experimental crystallographic conformation and the root–mean–square deviation (RMSD) values were calculated. RMSD values below 2.0Å confirmed the reliability and accuracy of the docking protocol.

### 2.5. Predication of Physicochemical and ADMET Properties

The physicochemical and ADMET-related properties of the compounds **4a-b**, **5a-b**, and **6a-b** were predicted using QikProp module in the Schrödinger software, as well as pkCSM (https://biosig.lab.uq.edu.au/pkcsm, accessed on 27 November 2025) and SwissADME (http://www.swissadme.ch/index.php, accessed on 27 November 2025) web servers. These complementary computational tools provide a comprehensive evaluation of the drug-likeness and safety of the analyzed compounds, indicating their potential to be effective therapeutics agents. QikProp and SwissADME were used to estimate ADME properties, while toxicity predictions were obtained using the pkCSM server. All the selected ligands were drawn using ChemDraw Professional 16.0 and subsequently entered into the SwissADME and pkCSM platforms as SMILE notations.

## 3. Results and Discussion

### 3.1. Synthesis

The flavone derivative **6d** was synthesized according to the multistep procedure illustrated in Scheme 1. The synthetic route commenced with the preparation of flavonol **4d**, obtained through a Claisen–Schmidt condensation between 2′-hydroxyacetophenone (**1**) and 2-thiophenecarboxaldehyde (**2**) [36], affording the intermediate 2′-hydroxychalcone (**3d**). This intermediate was subsequently cyclized to flavonol **4d** via Algar–Flynn–Oyamada oxidation [37], employing hydrogen peroxide as the oxidant and potassium hydroxide as the base. The overall yield for these two steps was 48%.

In the next stage, flavonol **4d** was reacted with 2-chloroacetonitrile in acetone under reflux conditions in the presence of potassium carbonate, yielding 3-O-cyanomethylflavone (**5d**) with a 69% yield. The final step involved a click reaction [38,39] between sodium azide (NaN_3_) and compound **5d**, catalyzed by ammonium chloride (NH_4_Cl), leading to the formation of the target flavone derivative **6d** with a 67% yield. All synthesized compounds were purified, either by recrystallization or silica gel column chromatography, and their purity was confirmed by thin-layer chromatography (TLC) analysis.

### 3.2. Characterization

All synthesized compounds were characterized using spectroscopic methods (IR, NMR) and high-resolution mass spectrometry (HRMS) to confirm their chemical structures.

The IR spectrum of chalcone **3d** (Figure S1; Supplementary Material) displayed distinct absorption bands at 1630 cm^−1^ and 1554 cm^−1^, corresponding to the carbonyl (C=O) and olefinic (C_2_=C_3_) functional groups, respectively. For flavonol **4d** (Figure S5), characteristic bands were observed at 3127 cm^−1^, 1593 cm^−1^, and 1556 cm^−1^, assigned to the hydroxyl group (O–H), carbonyl (C=O), and C=C stretching vibrations, respectively.

In the case of compound **5d** (Figure S9), the IR spectrum showed a notable cyano (C≡N) stretching band at 2184 cm^−1^, accompanied by the disappearance of the hydroxyl (O–H, position 3) absorption, confirming the formation of the cyanomethyl derivative. Finally, the IR spectrum of compound **6d** (Figure S13) revealed a broad absorption band of around 3018 cm^−1^, characteristic of the N–H stretching vibration of the tetrazole ring, thus supporting successful conversion to the final flavone–tetrazole structure.

The ^1^H NMR spectrum of chalcone **3d** displayed two characteristic olefinic proton signals, appearing as doublets of doublets at δ 8.05 and 7.43 ppm, corresponding to H-1 and H-2, respectively. In contrast, the spectrum of flavonol **4d** (Figure S2) showed the disappearance of the H-1 resonance, while the proton at C-2 in **3d** was replaced by a downfield signal at δ 10.23 ppm, attributed to the hydroxyl proton (OH).

For compound **5d** (Figure S6), the emergence of a singlet at δ 5.35 ppm, assigned to the methylene protons (-CH_2_-) of the cyanomethyl substituent, along with the absence of the OH signal, confirmed successful functionalization at position 3. In compound **6d** (Figure S10), this methylene resonance shifted further downfield to δ 5.66 ppm, consistent with the increased electron-withdrawing influence of the tetrazole ring relative to the nitrile group. Signal integrations and COSY spectral correlations (Figures S14 and S17) corroborated the proton assignments, thereby providing an unambiguous confirmation of the proposed structures of compounds **5d** and **6d**.

Similarly, the ^13^C NMR spectra confirmed that compounds **3d** through **6d** each exhibited the expected number of carbon signals, consistent with their molecular frameworks. In particular, compound **5d** (Figure S7) displayed a distinct resonance at δ 116.73 ppm, characteristic of the nitrile carbon (C≡N), while compound **6d** (Figure S11) showed a diagnostic signal at δ 136.38 ppm, corresponding to the tetrazole ring carbon.

Additional structural validation was obtained from complementary 2D NMR analyses, including DEPT, COSY, HSQC, and HMBC experiments (Figures S7, S8, S11, S12 and S14–S19; Supplementary Material), together with high-resolution mass spectrometry (HRMS) data. Collectively, these spectroscopic results provide strong evidence supporting the proposed structures of compounds **5d** and **6d**.

### 3.3. Anti-Inflammatory Assessments of the Compounds ***4*-*6***

The anti-inflammatory potential of the compounds **4-6** was evaluated based on their ability to inhibit COX-2 and 5-LOX. Molecular docking studies were performed on both target enzymes to explore possible binding orientation and to rationalize the trends observed in the in vitro enzymatic assay. This combined approach enables a structure–activity relationship analysis, highlighting how structural modification, particularly at position 3 of the flavonol core and within the aromatic substituents, affects both potency and selectivity toward the inflammatory enzymes.

#### 3.3.1. In Vitro Cyclooxygenase (COX-1/COX-2) and 5-Lipoxygenase Inhibition Assays

The anti-inflammatory potential of the synthesized derivatives (**4-6**) was evaluated through in vitro COX-1, COX-2, and 5-LOX inhibition assays using commercial screening kits. Indomethacin and quercetin served as reference inhibitors for cyclooxygenases and 5-LOX, respectively. IC_50_ values were calculated from three independent experiments and are summarized in Table 1. The enzymatic profiles of compounds **4-6** reveal distinct structure–activity relationships governed by heteroatom linkers and substituent effects.

**Figure 3:**
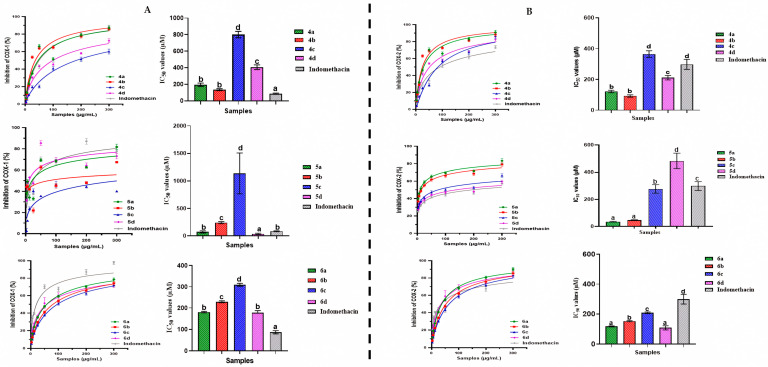
Concentration–response curves and IC_50_ bar graphs of the inhibitory activities of compounds **4-6** against COX-1 (**A**) and COX-2 (**B**). Indomethacin was used as a positive control. Values are expressed as mean ± SD (n = 3). Sigmoidal dose–response curves were fitted to determine IC_50_ values. Different letters indicate significant differences (one-way ANOVA followed by Tukey’s post hoc test, *p* ≤ 0.05).

As shown in Figure 3A, COX-1 inhibition varied markedly across the compound series. The unsubstituted flavonol **4a** (R′ = H) exhibited moderate COX-1-inhibitory activity, with an IC_50_ value of 194.55 ± 19.67 µM. The introduction of a para-methoxy substituent to compound **4b** resulted in a moderate improvement in activity (IC_50_ = 134.57 ±11.59 µM), suggesting that electron-donating groups may contribute to enhanced interactions within the COX-1 active site [40]. In contrast, heterocyclic derivatives **4c** (R = furanyl) and **4d** (R = thiofuranyl) showed significantly reduced COX-1-inhibitory activities (IC_50_ = 800.82 ± 39.04 and 405.75 ± 28.53 µM, respectively), suggesting that replacement of the phenyl ring with heteroaromatic bioisosteres may adversely affect ligand orientation and weaken productive interactions within the COX-1 catalytic pocket [41].

The introduction of a cyanomethyl group at position 3 generated the 3-O-cyanomethylflavone series (**5a-d**), which led to improved COX-1 inhibition in several cases. Compound **5a** (R′ = H) exhibited moderate inhibitory activity (IC_50_ = 74.62 ± 20.23 µM), representing a marked improvement over its parent compound **4a** and supporting a favorable contribution of the cyanomethyl substituent to COX-1 affinity. In contrast, derivative **5b** (R′ = OMe) showed reduced activity compared to **4b**, indicating that methoxy substitution within the O-cyanomethylflavones scaffold may be unfavorable for COX-1 inhibition. Among the heterocyclic analogues, compound **5d** (R = thiofuranyl) demonstrated the highest COX-1 inhibition within this series (IC_50_ = 39.11 ± 11.54 µM). While thiophene rings have been reported to enhance COX-1 inhibition in several scaffolds, our results indicate that this effect is not universal and is strongly influenced by the substitution pattern, particularly the presence of the O-cyanomethyl group, which appears to play a key role in activity [42]. This enhancement may be attributed to improved hydrophobic complementarity within the COX-1 active site, characterized by a narrow and predominantly lipophilic catalytic channel [43]. The transformation of the 3-O-cyanomethylflavones (**5a-d**) into their corresponding flavone–tetrazole hybrid (**6a-d**) generally resulted in reduced COX-1-inhibitory activity (Table 1, Figure 3A). This decrease may be attributed to the increased steric bulk and conformational rigidity introduced by the tetrazole ring, which could compromise optimal ligand accommodation within the narrow COX-1 catalytic channel. In addition, the incorporation of tetrazole moiety is known to reduce lipophilicity and increase polarity [44], factors that my further limit the favorable hydrophobic interactions required for effective binding within the lipophilic COX-1 active site.

The COX-2 inhibition followed a distinct structure–activity relationship, as illustrated in Figure 3B. The unsubstituted flavonol **4a** exhibited moderate COX-2 inhibition (IC_50_ = 122.06 ± 7.81 µM), while the para-methoxy substitution in compound **4b** provided a modest improvement in potency (IC_50_ = 92.63 ± 7.60 µM), suggesting that increased electron density in the aromatic ring my improve interactions within the COX-2 catalytic pocket. Replacement of the phenyl ring with a heterocyclic substituent (**4c-d**) resulted in reduced COX-2-inhibitory potency but increased selectivity indices (SI = 2.20 and 1.92, respectively), indicating a relative preference for COX-2 over COX-1. This behavior may be rationalized by the well-documented structural differences between COX-1 and COX-2, notably the substitution of ILE523 in COX-1 by VAL523 in COX-2, which creates a larger and more flexible side pocket that favors selective ligand adjustment [12]. Compounds **4a** and **4b** showed moderate COX-2 selectivity (SI = 1.45 and 1.59, respectively) compared with indomethacin (SI = 0.29), which preferentially inhibits COX-1. These results suggest that electron-donating substituents may enhance inhibitory potency, while heterocyclic substitutions mainly influence enzyme selectivity. Moreover, the introduction of the cyanomethyl group markedly enhanced both COX-2-inhibitory activity and selectivity. Compounds **5a** and **5b** exhibited IC_50_ values of 35.67 ± 2.92 and 46.44 ± 3.38 µM, respectively, significantly outperforming their corresponding flavonol precursors. Notably, compound **5b** displayed the highest selectivity index in the series (SI = 5.20), indicating a pronounced preference for COX-2. Among the heterocyclic derivatives, compound **5c** showed a modest improvement in inhibitory potency (IC_50_ = 276.90 ± 31.02 µM), accompanied by a substantial increase in selectivity (SI = 4.10), whereas compound **5d** did not exhibit a comparable enhancement.

The 3-O-tetrazolemethylflavone hybrids (**6a-d**) generally displayed reduced COX-2-inhibitory activity relative to their cyanomethyl counterparts (Table 1), consistent with the steric and conformational constraints imposed by the tetrazole moiety. Interestingly, heterocyclic derivatives **6c** and **6d** demonstrated improved COX-2 inhibition, with compound **6d** showing particularly enhanced potency and selectivity (IC_50_ = 107.87 ± 13.88 µM; SI = 1.65) compared with its precursor **5d** (IC_50_ = 482.84 ± 57.11 µM; SI = 0.08). This observation suggests that the combination of a thiofuranyl ring with a tetrazole scaffold may exert a synergistic effect, improving both inhibitory activity and selectivity toward COX-2.

**Figure 4:**
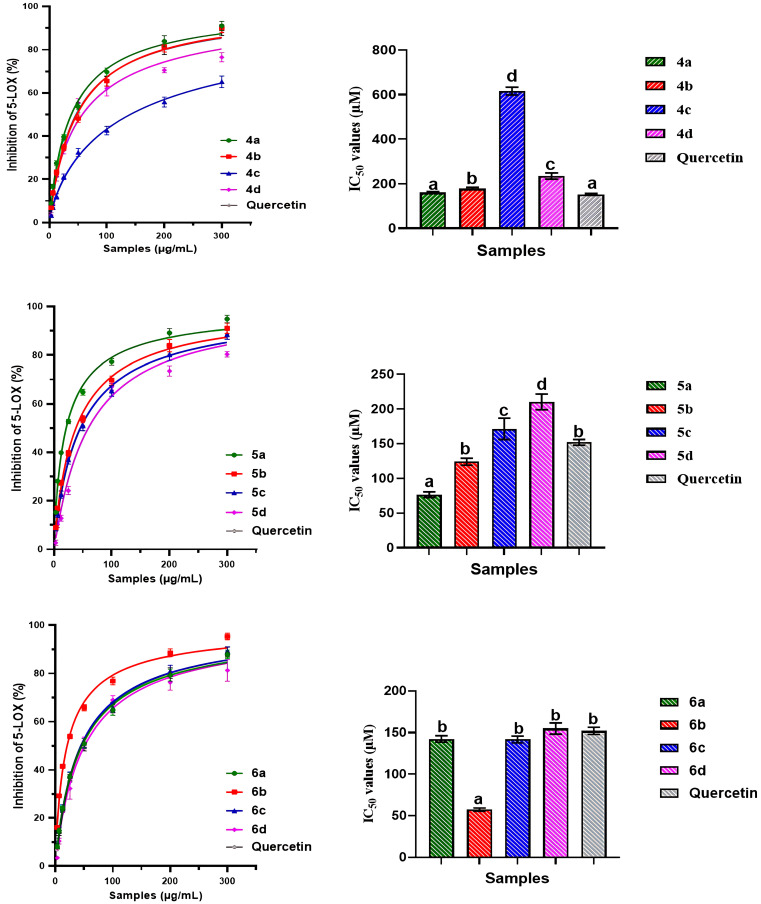
Concentration–response curves and IC_50_ bar graphs of the inhibitory activities of compounds **4-6** against 5-LOX. Quercetin was used as a positive control. Values are expressed as mean ± SD (n = 3). Sigmoidal dose–response curves were fitted to determine IC_50_ values. Different letters indicate significant differences (one-way ANOVA followed by Tukey’s post hoc test, *p* ≤ 0.05).

Regarding 5-LOX inhibition (Figure 4), the highest activity was observed for compound **6b**, which exhibited an IC_50_ value of 57.40 ± 2.03 µM. Its enhanced potency is likely related to the presence of the tetrazole moiety, which is known to interact efficiently with the iron-containing catalytic center of 5-LOX. Interestingly, despite its excellent 5-LOX-inhibitory effect, compound **6b** displayed only moderate COX-2 activity (IC_50_ = 152.23 ± 3.78 µM), suggesting that subtle differences in steric or electronic features govern the preferential inhibition of the lipoxygenase pathway. This observation highlights a trade-off between 5-LOX and COX-2 inhibition, where the tetrazole group enhances binding to the 5-LOX active site while partially compromising interactions within the COX-2 hydrophobic side pocket, likely due to steric bulk and conformational rigidity. Compounds **4a-b**, **5a-b**, and **6a-b** demonstrated both considerable COX-2 inhibition and moderate-to-strong 5-LOX inhibition, identifying them as promising dual COX-2/5-LOX inhibitors. Among these, **5b** and **5a** stand out as the most promising dual-acting candidates due to their strong COX-2 and 5-LOX inhibition, coupled with their weak inhibition of COX-1, reflected by high selectivity indices (SI = 5.20 and 2.09, respectively). This pharmacological profile is particularly desirable for the development of safer anti-inflammatory agents with reduced gastrointestinal side effects.

#### 3.3.2. In Silico Studies

##### Molecular Docking

Molecular docking studies were performed using GLIDE software (Maestro 13.4, Schrödinger Release 2022-4, LLC, New York, NY, USA) for the most active derivatives against COX-2 and 5-LOX in order to exploring possible binding modes and rationalizing trends observed in the in vitro enzymatic inhibitory activities. The moderate correlation between docking scores and experimental IC_50_ values reflects the inherent limitations of docking, which approximates binding affinity without fully considering protein flexibility, solvent interactions, or entropic contributions. The three-dimensional crystal structures of the selected target were obtained from the Protein Data Bank (PDB), with PDB codes 1CX2 for COX-2 and 6N2W for 5-LOX. The protein and ligand structures were prepared and energy-minimized using the OPLS4 force field at PH = 7 ± 2. The docking calculation employed the GLIDE scoring function for both pose prediction and estimation of binding energy (Kcal/mol) estimation, where more negative docking scores indicate a stronger predicted binding affinity.

To validate the docking protocol, the co-crystallized ligands (S58 for COX-2, and NDGA for 5-LOX) were re-docked into the active sites of the selected proteins. The resulting docked conformations were then superimposed onto the native co-crystal structures (Figure S20). Both conformations occupied the same binding pocket and exhibited the same interactions with the key active site residues. The calculated Root Mean Square Deviations (RMSD) of 0.27 Å for COX-2 and 1.9 Å for 5-LOX between the docked and experimental poses confirm the robustness of our docking method, as RMSD values below 2.0 Å are generally considered indicative of a highly reliable and reproducible docking procedure.

Subsequently, derivatives **4-6**, which exhibited in vitro dual-inhibitory effects against cyclooxygenase-2 and 5-lipooxygenase, were docked in the active site of both target enzymes (COX-2 and 5-LOX).

**a. Docking studies on COX-2 enzyme**

**Figure 5:**
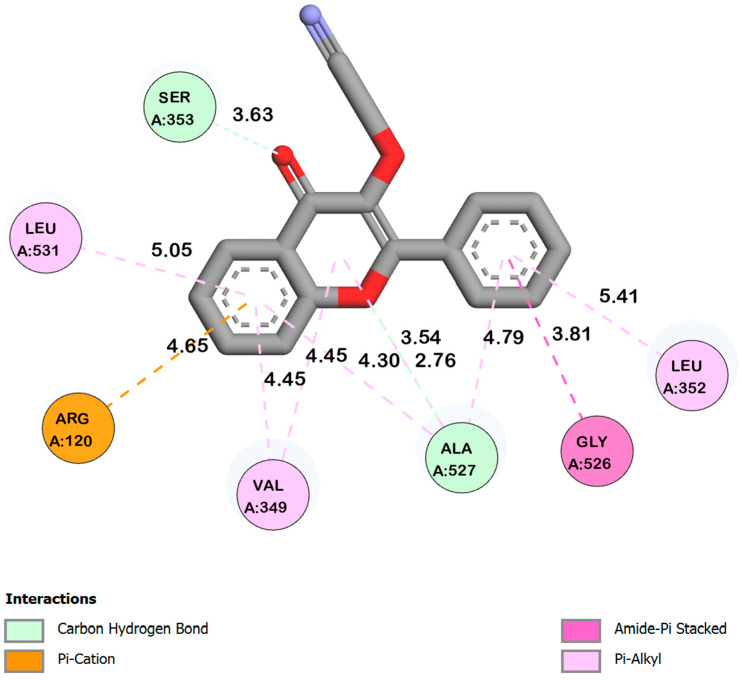
2D ligand–protein interaction diagram of derivative **5a** against COX-2 (PDB code: 1CX2). The numbers shown in black represent the interatomic distances (in Å) between the ligand and the corresponding amino acid residues, as calculated by Discovery Studio Visualize.

All the synthesized compounds (**4-6**) were docked into the COX-2 binding pocket of (PDB: 1CX2). The results (Table S1) show that the selected compounds exhibited favorable binding affinities, with docking scores ranging from −6.754 to −8.827 Kcal/mol, in moderate agreement with the experimental inhibition data (correlation indices r = 0.545). The O-cyanomethylflavones (**5a-b**), which displayed the strongest COX-2-inhibitory activity in vitro, with IC_50_ values of 35.67 and 46.44 µM, respectively, showed comparatively low binding energies of −8.827 and −8.247 kcal/mol (Table 2). These results suggest that the cyano group may interact more effectively with the COX-2 active site, comprising the hydrophobic cavity (ARG120, SER530, TYR385) and the hydrophilic side pocket (VAL434, LEU503, ARG513, VAL523), compared with the tetrazole ring [45,46]. Notably, compound **5a** forms a π–cation interaction with ARG120 at a distance of 4.65 Å (Figure 5), a residue located at the entrance of the active-site cavity and known to play a key role in substrate orientation [45]. Additionally, the pyran ring establishes carbon–hydrogen bonds with SER353 and ALA527, enabling selective interaction with the COX-2 pocket while sparing COX-1. Derivative **5a** also showed a π–alkyl interaction with VAL349, LEU331, LEU352, and ALA527, highlighting the complementarity between the ligand’s hydrophobic regions and the lipophilic side pocket of COX-2 active site. These molecular features may explain the highest docking score and significant anti-COX-2 activity observed in the in vitro assay.

**Figure 6:**
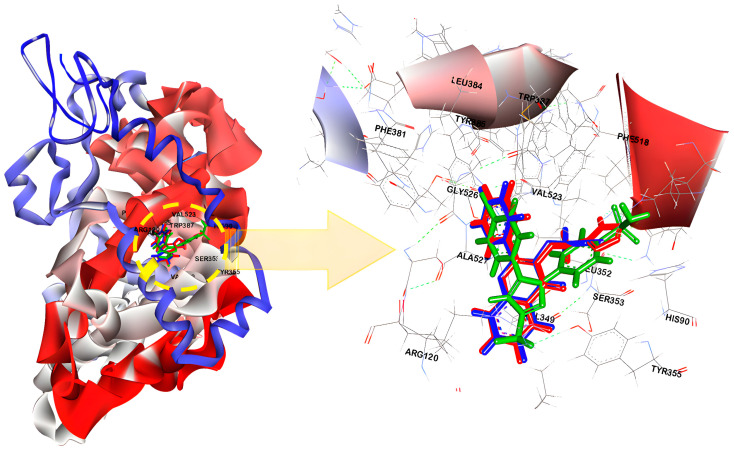
3D Binding mode of compounds **5a** (blue), **6a** (red), and **S58** (green) inside the active site of COX-2 (PDB: 1CX2).

The 3D binding pose of compounds **5a** (blue) and **6a** (red) in the COX-2 active site (Figure 6) indicate that these derivatives adopt a binding orientation that is highly consistent with the reference ligand **S58** (green). Moreover, compounds **5a** and **6a** slightly mimic the placement of the reference ligand, as indicated by the alignment between the aromatic rings of the synthesized compounds and the reference ligand, allowing them to exploit the same hydrophobic interaction formed with residues such as VAL349, LEU352 and ALA527.

The binding poses of the selected derivatives (**5a** and **6a**) adopt an L-shaped conformation, in which one portion of the molecule extends into the hydrophobic channel while the other reaches toward the polar side pocket of COX-2. This arrangement allows flavone cores to penetrate deep into the lipophilic cavity, maximizing the hydrophobic interaction, while positioning polar substituents near residue, such as SER353 and GLY526. This conformation is critical for selective COX-2 inhibition, as it allows the compounds to bind the COX-2-specific side pocket.

These findings suggest that the flavone cores of compounds **5a** and **6a,** in combination with their L-shaped orientation, play an essential role in stabilizing interactions within the COX-2 active site [47], thereby contributing to their selective inhibitory activity.

**Figure 7:**
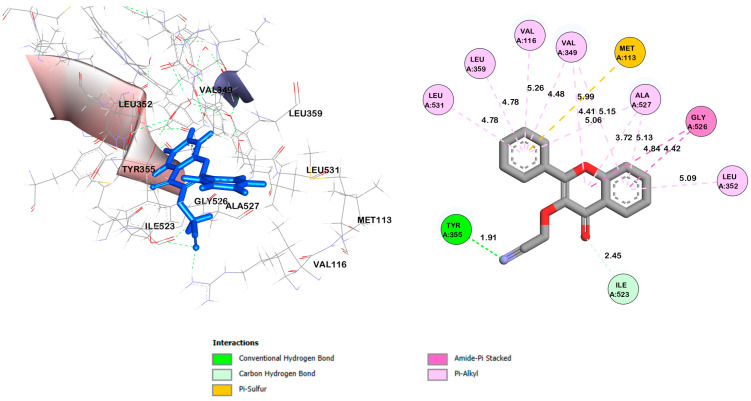
2D and 3D ligand–protein interaction diagram of derivatives **5a** against COX-1 (PDB code: 1EQG). The numbers shown in black represent the interatomic distances (in Å) between the ligand and the corresponding amino acid residues, as calculated by Discovery Studio Visualize.

To rationalize the observed COX-2 selectivity, compound **5a** was additionally docked into the COX-1 active site (PDB: 1EQG) and directly compared with its binding mode in COX-2. In contrast to the deep L-shaped insertion observed in COX-2 (Figure 6), compound **5a** adopted a more superficial and restricted conformation within the narrower COX-1 catalytic channel. In COX-1, the ligand was mainly stabilized by hydrophobic π-alkyl interaction with residues such as VAL116, VAL349, LEU352, LEU359, ALA527 and LEU531, together with a single hydrogen bond with TYR355 (Figure 7). These interactions are typical of nonspecific hydrophobic anchoring and do not compensate for the absence of deeper pocket engagement. A key structural factor governing this behavior is the ILE523 residue in COX-1, which sterically blocks access to the secondary side pocket that is present in COX-2 due to the substitution of ILE in position 523 by VAL [48]. In COX-2, this substitution creates an additional hydrophobic cavity that accommodates bulky or polar substituents and enables the L-shaped binding mode observed for compound **5a** (Figure 6). The cyanomethyl substituent of **5a** is therefore able to extend into this COX-2-specific pocket and form stabilizing interactions with residues such as ARG120 and ALA527, which are not accessible in COX-1. Consequently, the inability of **5a** to exploit the COX-2 side pocket results in weaker stabilization and reduced inhibitory potency toward COX-1, consistent with the experimentally determined selectivity index (SI = 2.02). This comparative docking analysis thus provides a clear molecular basis for the preferential inhibition of COX-2 by compound **5a** and further supports the SAR trend that cyanomethyl substitution at the 3-position of the flavonol core enhances COX-2 selectivity.

**b. Docking studies on 5-LOX enzyme**

**Figure 8:**
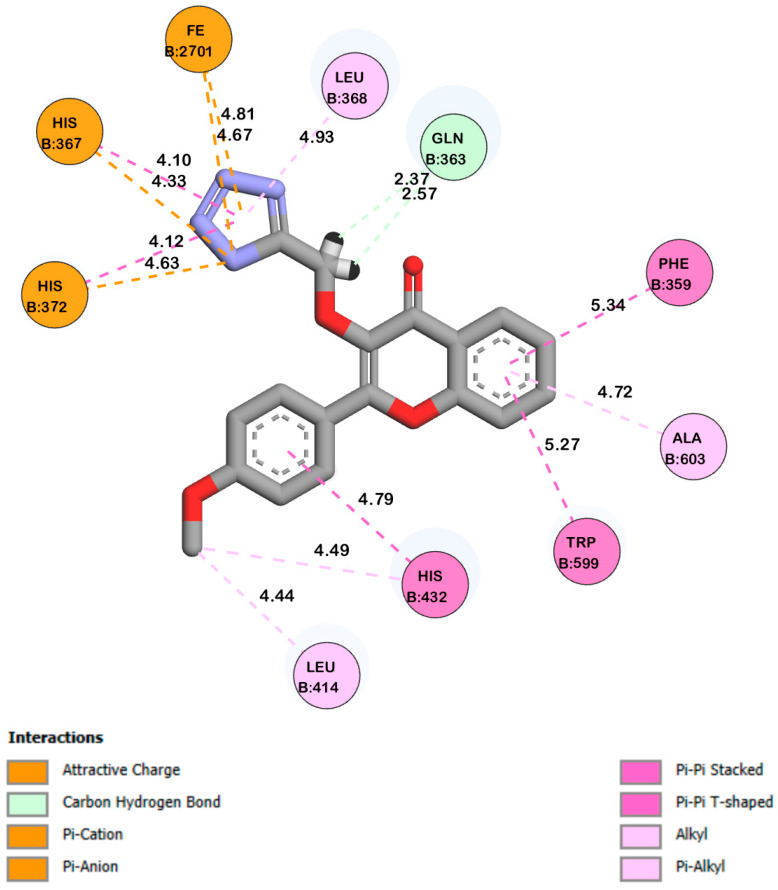
2D ligand protein interaction diagram of derivatives **6b** against 5-LOX active site (PDB; 6N2W). The numbers shown in black represent the interatomic distances (in Å) between the ligand and the corresponding amino acid residues, as calculated by Discovery Studio Visualize.

In the case of 5-Lipooxygenase, the docking results revealed that the selected derivatives (**4a-b**, **5a-b**, and **6a-b**) exhibited a moderate predicted binding within the enzyme’s active site, with docking scores ranging from −4.272 to −5.734 Kcal/mol (Table S1; see Supplementary Material). Among these compounds, the flavone–tetrazole hybrid **6b,** identified as the most potent inhibitor in vitro (IC_50_ = 57.40 µM), displayed the most favorable docking scores (Table 2, −5.734 Kcal/mol). The predicted binding pose (Figure 8) indicates that the tetrazole moiety is positioned near the catalytic center, where it forms a π–cation interaction with Fe^3+^. Moreover, the negative charge of nitrogen atoms at position 5 of the tetrazole ring establishes an attractive interaction that helps anchor the compound in a catalytically relevant orientation. Derivative **6b** also engages through electrostatic contacts, as well as π–π stacked and π–π T-shaped interactions. Additionally, compound **6b** forms two stable carbon hydrogen bonds with GLN363 at distances of 2.37 and 2.57Å, respectively, stabilizing the ligand at the entrance of substrate channel.

The flavone core of the **6b** ranges deep into the hydrophobic cavity, establishing multiple π–alkyl interactions with LEU414 and ALA603, as well as π–π stacked and π–π T-shaped interactions with the HIS432, PHE359, and TRP599 key residues lining the long substrate-binding tunnel of 5-LOX [35,49]. These contacts align with the characteristic binding pattern of substrate-competitive 5-LOX inhibitors, which rely on the hydrophobic channel being filled to prevent arachidonic acid from accessing the catalytic site [50].

Finally, the proximal interactions with the iron, along with multiple hydrophobic contacts, support the observed inhibitory potential of derivative **6b** (IC_50_ = 57.40 µM), suggesting a stabilizing effect within the active site that blocks substrate access and thereby inhibits the catalytic turnover rate of 5-LOX.

**Figure 9:**
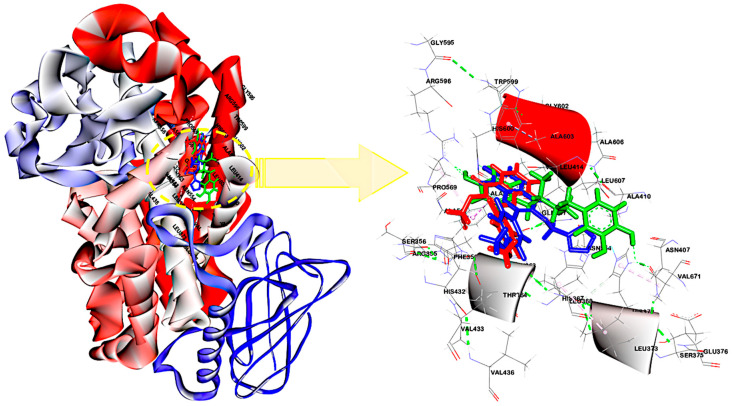
3D binding mode of compounds **5b** (red), **6b** (blue), and **NDGA** (green) inside the active site of 5-LOX (PDB: 6N2W).

Regarding the 3D binding poses of the selective compounds **5b** (red), **6b** (blue) in 5-LOX (Figure 9), the results show that both ligands occupy the active site in a manner closely comparable to the reference inhibitor NDGA (green), though notable differences appear in binding depth, orientation, and specific residue interactions. Compound **6b** and NDGA share key contacts with HIS367 and TRP599, residues that contribute to the central hydrophobic cavity and play an essential role in stabilizing bound ligands and controlling substrate entry. However, compound **6b** adopts a distinct U-shaped conformation, enabling additional interactions beyond those observed for NDGA. This folded geometry allows **6b** to extend farther toward the Fe-proximal region as well as the upper hydrophobic pocket, suggesting more extensive engagement within the active site. In contrast, compound **5b** displays a different orientation compared to **6b**. Its cyanomethyl group adopts an opposite orientation relative to the tetrazole moiety of **6b**. Moreover, compound **5b** does not adopt the U-shaped conformation, limiting its access to the Fe-proximal region and to deeper pocket residues [35,51,52].

This finding indicates that compound **6b** preserves essential NDGA interactions while expanding its interactions along the substrate tunnel, which enhances the stabilization toward the active site of 5-lipooxygense.

Despite the qualitative agreement observed between docking scores and the in vitro inhibitory profile, the overall correlation remains moderate, reflecting a well-known limitation of docking in the prediction of biological activities. Docking scores are derived from a static scoring function that approximates binding affinity but neglects critical factors such as solvent effects and entropic contribution, as well as protein flexibility [53,54,55]. For COX-2, subtle variations in ligand orientation, hydration of the active site, and interactions within the flexible side pocket can significantly influence inhibitory potency [56], while being only partially captured by docking calculations. These limitations are even more pronounced for 5-LOX, which is recognized as a highly dynamic enzyme undergoing substantial conformational rearrangements during substrate binding and catalysis, including changes associated with iron coordination and redox state [57]. Moreover, the in vitro inhibitory activity depends not only on the binding affinity but also on physicochemical and biological factors such as aqueous solubility, stability and potential allosteric or off-target effects, none of which are considered in docking simulations [58]. Consequently, molecular docking should be regarded as an exploratory tool that provides qualitative insights into possible binding modes rather than a quantitative predictor of enzymatic inhibition, explaining the observed moderate correlation with the experimental data.

##### Assessment of ADMET Properties

In Silico Prediction of Pharmacokinetic ADMET Parameters

**Figure 10:**
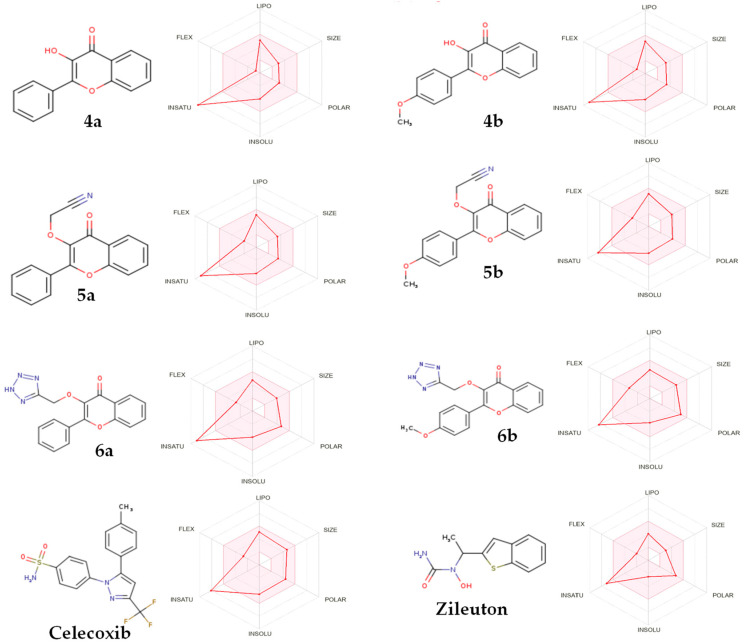
SwissADME bioavailability radar analysis of compounds **4a-b**, **5a-b**, **6a-b**, **celecoxib**, and **zileuton**.

The Swiss ADME bioavailability radar analysis (Figure 10) revealed that the selected compounds (**4a-b**, **5a-b**, and **6a-b**) fall within the acceptable limits of drug-like properties associated with orally active small molecules, except for the INSATUR parameter. This deviation is commonly observed for flavonoid-based scaffolds and does not necessarily preclude oral bioavailability. The radar plot indicates that the selected derivatives exhibited a balanced bioavailability profile, falling between those of the reference drugs celecoxib and Zileuton, supporting their classification as drug-like molecules at an early discovery stage.

The ADMET evaluation of these derivatives (Table S2; see Supplementary Material) revealed physicochemical trends that may influence their inhibitory profile against COX-2 and 5-LOX. Regarding solubility, compound of series “A” displayed moderate lipophilicity, with QPlogPo/W values ranging from 2.003 to 2.594, along with moderate aqueous solubility, defined by QPlogS values between −3.124 and −4.154. In contrast, series “B” exhibited slightly higher lipophilicity and acceptable aqueous solubility (QPlogS ranging from −3.101 to −4.321), suggesting that the bioavailability of compounds **4a**-**6a** may be slightly improved under physiological conditions compared with compounds **4b**-**6b**. For reference, celecoxib demonstrated higher lipophilicity (QPlogPo/W = 3.321) but poor solubility (QPlogS = −5.769) [59], whereas zileuton showed lower lipophilicity (QPlogPo/W = 0.927) and higher solubility (QPlogS = −1.674). Together, these findings indicate that the selected derivatives occupy an intermediate range that balances both solubility and lipophilicity.

The solvent-accessible surface area analysis (SASA), highlights the balanced exposure of polar and nonpolar regions. For example, compound **5a** principally exhibited a polar exposed surface (FISA = 111.708 Å^2^; FOSA = 36.804 Å^2^), which may facilitate aqueous solvation and interaction with polar residues in enzyme active sites, whereas compounds **5b** and **6b** showed hydrophobic exposure (FOSA = 130.357 and 130.594 Å^2^, respectively), that may favor binding within the predominantly lipophilic catalytic channels of COX-2 and 5-LOX. These trends are consistent with the observed enzymatic inhibition profiles and suggest that a balanced distribution of polar and hydrophobic surface areas may contribute to dual inhibitory activity. Additionally, the selected derivatives (**4a-b**, **5a-b**, and **6a-b**) demonstrated a favorable predicted membrane permeability (QPPCaco from 459.331 to 1297.581 nm/s and QPPMDCK between 213.377 and 655.594 nm/s), and high predicted human oral absorption (86 to 100%), suggesting that this derivatives are compatible with oral administration, which is essential for chronic anti-inflammatory therapy. However, the QPlogBB values suggest limited central nervous system penetration, a feature that is desirable for peripheral anti-inflammatory activity [60], while the QPlogHERG predictions indicate a low cardiotoxicity risk.

Based on the in vitro assays, O-cyanomethylflavones **5a** and **5b** exhibited the strongest COX-2 inhibition (IC_50_ = 35.67 ± 2.92 and 46.44 ± 3.38 µM, respectively), along with moderate inhibition of 5-LOX (IC_50_ = 76.60 ± 4.04 and 124.05 ± 4.91µM, respectively). These results are consistent with their moderate lipophilicity, SASA values, and favorable distribution of polar (FISA) and hydrophobic (FOSA) surface areas, which collectively support efficient interactions with both polar residues and hydrophobic pockets within the COX-2 and 5-LOX active sites. Additionally, compound **6b** demonstrated the highest inhibition of 5-LOX, with IC_50_ values of 57.40 ± 2.03 µM, a behavior likely influenced by its greater hydrophobic surface area (FOSA = 130.594 Å^2^) compared with compound **6a** (IC_50_ = 142.61 ± 3.81 µM), which displays considerably lower hydrophobic exposure (FOSA = 43.043 Å^2^).

Finally, the predicted ADME profile is consistent with the observed biological activities, suggesting that moderate lipophilicity and balanced solubility may contribute to the dual enzymatic inhibition of COX-2 and 5-LOX. Moreover, the favorable in silico pharmacokinetic properties of the synthesized derivatives indicate their potential as promising candidates for further investigation as anti-inflammatory agents. While these ADME predictions provide useful preliminary insights, experimental studies will be required to confirm the pharmacokinetic behavior, metabolic stability, and safety of these compounds.

In Silico Prediction of Toxicity Parameters and Safety Profile of Compounds **4a-b**, **5a-b**, and **6a-b**

The results obtained from pkCSM and presented in Table S3 (see Supplementary Material) provide an insight into the predicted toxicity and safety profiles of the selective compounds (**4a-b**, **5a-b**, **6a-b**) in comparison with the reference drugs celecoxib (selective COX-2 inhibitor) and Zileuton (5-LOX inhibitor).

Regarding the metabolic standpoint, none of the synthesized derivatives were predicted to be substrates of CYP2D6, which markedly reduces the likelihood of drug–drug interactions. Conversely, all the selected compounds, similarly to celecoxib, were predicted to be substrates of CYP3A4, suggesting a degree of metabolic dependance on this major isoenzyme. However, unlike celecoxib, which inhibited CYP3A4, the synthesized analogues were not predicted to inhibit CYP3A4 and CYP2D6 isoforms, indicating a reduced risk of drug–drug interaction being mediated through these metabolism dependances. All the synthesized compounds, along with the reference drug, inhibited CYP1A2, indicating moderate drug–drug interactions that remain comparable to celecoxib and Zileuton. Among them, compounds **5b**, **6a**, and **6b** exhibited minimal CYP inhibition and therefore possess the most favorable predicted metabolic safety profile.

Regarding the excretion profile, the predicted total clearance values (−0.0076 to 0.325 log mL/min/kg) suggest a moderate systemic elimination, and therefore a potentially prolonged half-life. Furthermore, the absence of renal OCT2 substrate in the case of compounds **4a**, **6a**, and **6b** indicates a reduced risk of renal accumulation and nephrotoxicity, reflecting a favorable predicted renal safety profile [61].

In terms of toxicity profile, all the synthesized derivatives were predicted to be non-mutagenic (negative AMES toxicity) and lacked hERG I/II inhibition, indicating a low predicted cardiotoxic risk. The estimated maximum tolerated doses suggest that compounds **6a** and **6b** (0.596 and 0.580, respectively) could be safely administrated within moderate dose ranges. Likewise, the predicted LOAEL values (0.811–2.945) and LD_50_ values (1.673–2.7) indicate relatively low subchronic and acute toxicity; these in silico results support their suitability as lead compounds rather than fully optimized drug candidates. Importantly, we acknowledge that these findings are based on computational models and require experimental validation, particularly through cellular toxicity assays and in vivo pharmacokinetic studies, to confirm their translational potential.

Finally, this in silico ADMET evaluation of derivatives **5b** and **6a-b** suggests that these compounds display physicochemical and pharmacokinetic properties compatible with early-stage drug discovery. The predicted oral absorption and low toxicity profiles support their drug-likeness and suggest a favorable safety margin for further development. Nevertheless, their moderate predicted metabolic stability may limit systemic exposure, representing a potential challenge for in vivo efficacy. From a medicinal chemistry perspective, these ADMET findings emphasize the need for structural optimization aimed at improving their metabolic robustness while preserving their dual COX-2/5-LOX-inhibitory activity. Such modification could enhance the pharmacokinetic profile and strengthen the translational potential of these compounds as orally active anti-inflammatory agents.

## 4. Conclusions

In this study, we reported the synthesis of new flavones derivatives (**5a-d** and **6a-d**), and their in vitro enzymatic evaluation against COX-1, COX-2, and 5-LOX, supported by molecular docking and in silico ADMET analyses. The biological screening showed that compounds **4-6** exhibited moderate to excellent inhibitory activities, with IC_50_ values ranging from 35.67 ± 2.92 to 1137.44 ± 371.05 µM. Among them, derivatives **5a-b**, **4a-b**, and **6a-b** emerged as the most promising candidates based on their dual COX-2/5-LOX enzymatic inhibition profile. Notably, compounds **5a** and **5b** demonstrated the most pronounced anti-inflammatory activity. Both exhibited strong COX-2 inhibition activity (**5a**: IC_50_ = 35.67 ± 2.92 µM; **5b**: IC_50_ = 46.44 ±3.38 µM) along with meaningful inhibition of 5-LOX (**5a**: IC_50_ = 76.60 ± 4.04 µM; **5b**: IC_50_ = 124.05 ± 4.91 µM). Importantly, they maintained low COX-1 inhibition, reflected in their elevated selectivity indices (SI = 2.09 for **5a** and SI = 5.20 for **5b**).

Molecular docking results suggest a favorable binding mode for compounds **5a** and **5b** within the COX-2 active site (PDB: 1CX2), supporting their in vitro activities. Similarly, the flavone tetrazole hybrid **6b** exhibited the most potent inhibition of 5-LOX (IC_50_ = 57.40 ± 2.03µM), accompanied by favorable interactions within the 5-LOX active site (PDB: 6N2W), likely facilitated by the tetrazole moiety. Additionally, the in silico ADME and toxicity prediction indicated that these dual inhibitors possess acceptable drug-like physicochemical proprieties, favorable oral absorption, and low predicted toxicity for the most active compounds. However, these predictions remain preliminary and require experimental validation, particularly through cellular toxicity and pharmacokinetic studies.

Overall, the present work identifies compounds **5a**, **5b**, and **6b** as early-stage lead candidates for the development of dual COX-2/5-LOX inhibitors and suggests that further structural optimization and biological validation at the cellular and in vivo levels will be necessary to assess their translational potential as anti-inflammatory agents.

## Figures and Tables

**Table 1:** In vitro cyclooxygenase (COX-1/COX-2) and 5-LOX inhibition activities of the compounds **4-6**.

No.	Structures	R′	X	IC_50_ µM			
				COX-1	COX-2	SI	5-LOX
**4a**	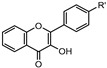	H	-	194.55 ± 19.67	122.06 ± 7.81	1.59	160 ± 4.70
**4b**		OMe	-	134.57 ± 11.59	92.63 ± 7.60	1.45	177.77 ± 5.62
**4c**	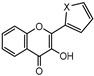	-	O	800.82 ± 39.04	364.63 ± 21.43	2.20	615.94 ± 17.70
**4d**		-	S	405.75 ± 28.53	211.53 ± 14.37	1.92	234.34 ± 14.3
**5a**	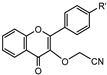	H	-	74.62 ± 20.23	35.67 ± 2.92	2.09	76.60 ± 4.04
**5b**		OMe	-	241.65 ± 19.10	46.44 ± 3.38	5.2	124.05 ± 4.91
**5c**	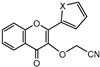	-	O	1137.44 ± 371.05	276.9 ± 31.02	4.11	171.34 ± 15.12
**5d**		-	S	39.11 ± 11.54	482.84 ± 57.11	0.08	210.24 ± 11.22
**6a**	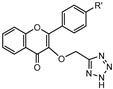	H	-	180.92 ± 3.37	117.42 ± 3.37	1.54	142.61 ± 3.81
**6b**		OMe	-	229.1 ± 4.57	152.23 ± 3.78	1.50	57.40 ± 2.03
**6c**	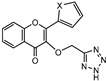	-	O	309.51 ± 6.83	208.7 ± 5.09	1.48	141.88 ± 4.03
**6d**		-	S	177.70 ± 10.14	107.87 ± 13.88	1.65	154.94 ± 6.68
**Indomethacin**				86.47 ± 7.63	298.5 ± 32.34	0.29	-
**Quercetin**				-	-	-	152.06 ± 4.2

SI = IC_50_ (COX-1)/IC_50_ (COX-2).

**Table 2:** The binding affinities and the contributing binding residues of COX-2 and 5-LOX with compounds **4a-b**, **5a-b**, and **6a-b**.

**COX-2 (PDB: 1CX2; RMSD = 0.27)**				
**Compounds**	**Docking Score (Kcal/mol)**	**Contributing Binding Residues**	**Hydrogen Bonds**	**Distance (Å)**
**4a**	−7.897	VAL349, LEU352, VAL523, GLY526, ALA527	-	-
**4b**	−8.198	HIS90, GLN192, VAL349, LEU352, SER353, ALA516, VAL523, GLY526, ALA527	HIS90	2.16
**5a**	−8.827	ARG120, VAL349, LEU352, SER353, GLY526, ALA527, LEU531	-	-
**5b**	−8.247	HIS90, GLN192, LEU352, SER353,ALA516, VAL523, ALA527	-	-
**6a**	−6.754	HIS90, ARG120, VAL349, LEU352, SER353, ALA516, PHE518, VAL523, GLY526, ALA527, LEU531	HIS90 LEU352	2.72 2.62
**6b**	−6.941	HIS90, VAL116, GLN192, VAL349, LEU352, SER353, TYR355, LEU359 ALA516 VAL523, GLY526, ALA527, LEU531	HIS90 TYR355	2.25 2.15
**S58**	−9.235	HIS90, ARG120, VAL349, LEU352, SER353, TYR355, PHE381, LEU384, TYR385, TRP387, PHE518, VAL523, GLY526, ALA527	HIS90 ARG120 TYR355 PHE518	2.79 2.10 2.11; 2.77 3.08
**5-LOX (PDB: 6N2W; RMSD = 1.9)**				
**4a**	−5.238	PHE359, LEU368, HIS432, ARG596, TRP599, ALA603	ARG596	6.19
**4b**	−4.272	PHE359, LEU414, HIS432, ARG596, TRP599, ALA603	ARG596	6.05
**5a**	−5.359	PHE359, HIS360, THR364, PRO569, ARG596, TRP599, ALA603	THR364 ARG596	3.02 5.94
**5b**	−5.190	PHE359, HIS360, LEU414, HIS432, ARG596, TRP599, ALA603	ARG596	6.14
**6a**	−4.660	PHE359, ARG596, ALA603	ARG596	5.73; 2.60; 2.13
**6b**	−5.734	PHE359, GLN363, HIS367, LEU368, HIS372, LEU414 HIS432, TRP599, ALA603	-	-
**NDGA**	−7.656	HIS367, ASN407, ALA410, ARG596, TRP599, HIS600, LEU607	ARG596 HIS600	2.51 2.31; 2.57

## Data Availability

The original contributions presented in this study are included in the article/Supplementary Material. Further inquiries can be directed to the corresponding author.

## Supplementary Materials

The following supporting information can be downloaded at: https://www.mdpi.com/article/10.3390/cimb48030243/s1.

## Abbreviations

The following abbreviations are used in this manuscript:

COX-1	Cyclooxygenase-1.
COX-2	Cyclooxygenase-2.
5-LOX	5-Lipooxygenase.
^1^H-NMR	Proton Nuclear Magnetic Resonance spectroscopy.
^13^C-NMR	Carbon-13 Nuclear Magnetic Resonance spectroscopy.
IR	Infrared Spectroscopy.
