# Supplementary material for: Design, Synthesis, and In Vitro Enzymatic Evaluation of Novel Flavone Derivatives as Dual COX-2/5-LOX Inhibitors Supported by Molecular Docking and ADMET Analysis
Source: Curr Issues Mol Biol. 2026 Feb 25;48(3):243. doi: 10.3390/cimb48030243 (PMC13025331; doi:10.3390/cimb48030243)
Supplement: Supplementary file 1 [file cimb-48-00243-s001.zip › cimb-4166381-supplementary.pdf]

Supporting Information

Design, Synthesis, and Biological Evaluation of Novel Flavone Derivatives as Dual COX-2/5-LOX Inhibitors Supported by Molecular Docking and ADMET Analysis

Elmehdi Fraj[1], Amine Elbouzidi [2], Haytham Bouammali[1], Hanane Jaouani[1], Chaymae Bourhou[1], Mohammed Addi[2], Susu M Zughaier [3], Allal Challioui[1], Rachid Touzani[1], and Boufelja Bouammali[1,*]

[1] *Laboratory of applied Chemistry and Environment, Faculty of Sciences, Mohammed First University, 60000 Oujda, Morocco.*

[2] *Laboratory for Agricultural Production Improvement, Biotechnology, and Environment (LAPABE), Faculty of Sciences, Mohammed Premier University, Oujda 60000, Morocco.*

[3] *College of Medicine, QU Health, Qatar University, Doha, Qatar.*

*Address correspondence to this author at the Laboratory of applied Chemistry and Environment, Faculty of Science, University Mohammed First, Oujda, Morocco. Tel : +212 662134880 ; E-mail : b.bouammali@ump.ac.ma

Table of contents:

**Figure S1.** FTIR spectrum of compound **3d**	**P.3**
**Figure S2.** ^{1}H-NMR spectrum of compound **4d.**	**P.3**
**Figure S3.** ^{13}C-NMR spectrum of compound **4d.**	**P.4**
**Figure S4.** MS spectrum of compound **4d.**	**P.4**
**Figure S5.** FTIR spectrum of compound **4d.**	**P.5**
**Figure S6.** ^{1}H-NMR spectrum of compound **5d.**	**P.5**
**Figure S7.** ^{13}C-NMR spectrum of compound **5d.**	**P.6**
**Figure S8.** MS spectrum of compound **5d.**	**P.6**
**Figure S9.** FTIR spectrum of compound **5d.**	**P.7**
**Figure S10.** ^{1}H-NMR spectrum of compound **6d.**	**P.7**
**Figure S11.** ^{13}C-NMR spectrum of compound **6d.**	**P.8**
**Figure S12.** MS spectrum of compound **6d.**	**P.8**
**Figure S13.** FTIR spectrum of compound **6d.**	**P.9**
**Figure S14.** COSY spectrum of compound **5d.**	**P.9**
**Figure S15.** HMBC spectrum of compound **5d.**	**P.10**
**Figure S16.** HMQC spectrum of compound **5d.**	**P.10**
**Figure S17.** COSY spectrum of compound **6d.**	**P.11**
**Figure S18.** HMBC spectrum of compound **6d.**	**P.11**
**Figure S19.** HMQC spectrum of compound **6d**	**P.12**
**Figure S20.** Interactions of the active site residues with native cocrystal (Red) in the complex and the predicted docking pose of cocrystal (Bleu).	**P.12**
**Table S1.** The binding affinities and the contributing binding residues of COX-2 and 5-LOX with compounds **4-6.**	**P.13**
**Table S2.** In silico Lipinski's rule of five and Predicted ADMET analysis for selected derivatives **4a-b**, **5a-b**, and **6a-b**.	**P.15**
**Table S3.** Predicted metabolism, exertion, and toxicity parameters of flavones derivatives **4a-b**,**5a-b,** and **6a-b** using pkCSM	**P.16**

Figure S21-S56. NMR and HRMS spectra of derivatives 4a-c, 5a-c , and 6a-c	p.17-36

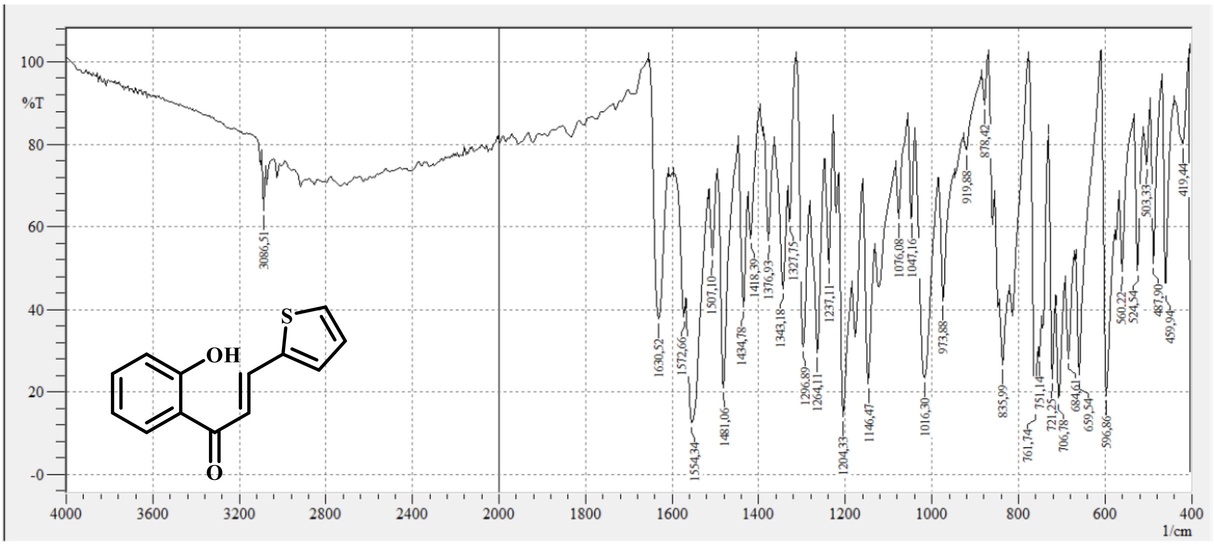

Figure S1. FTIR spectrum of compound **3d**

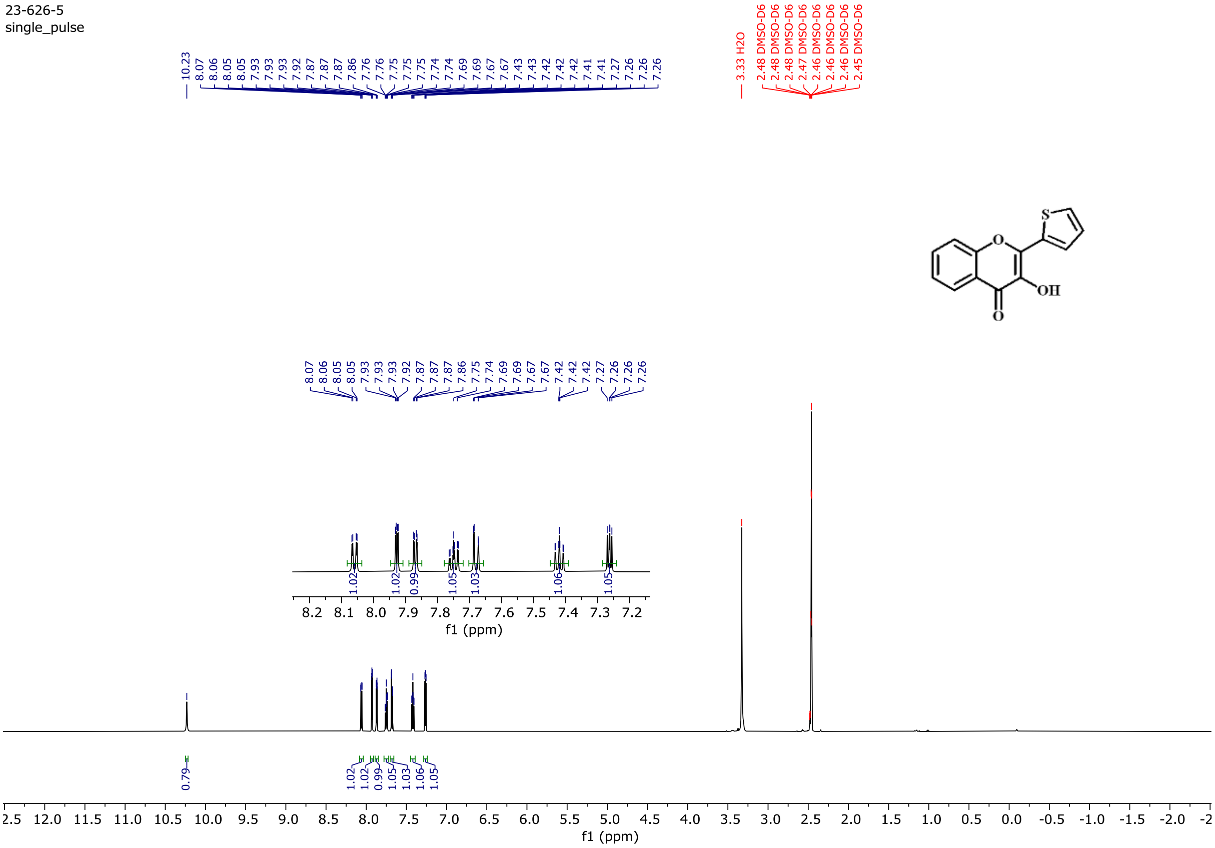

Figure S2. ^{1}H-NMR spectrum of compound **4d.**

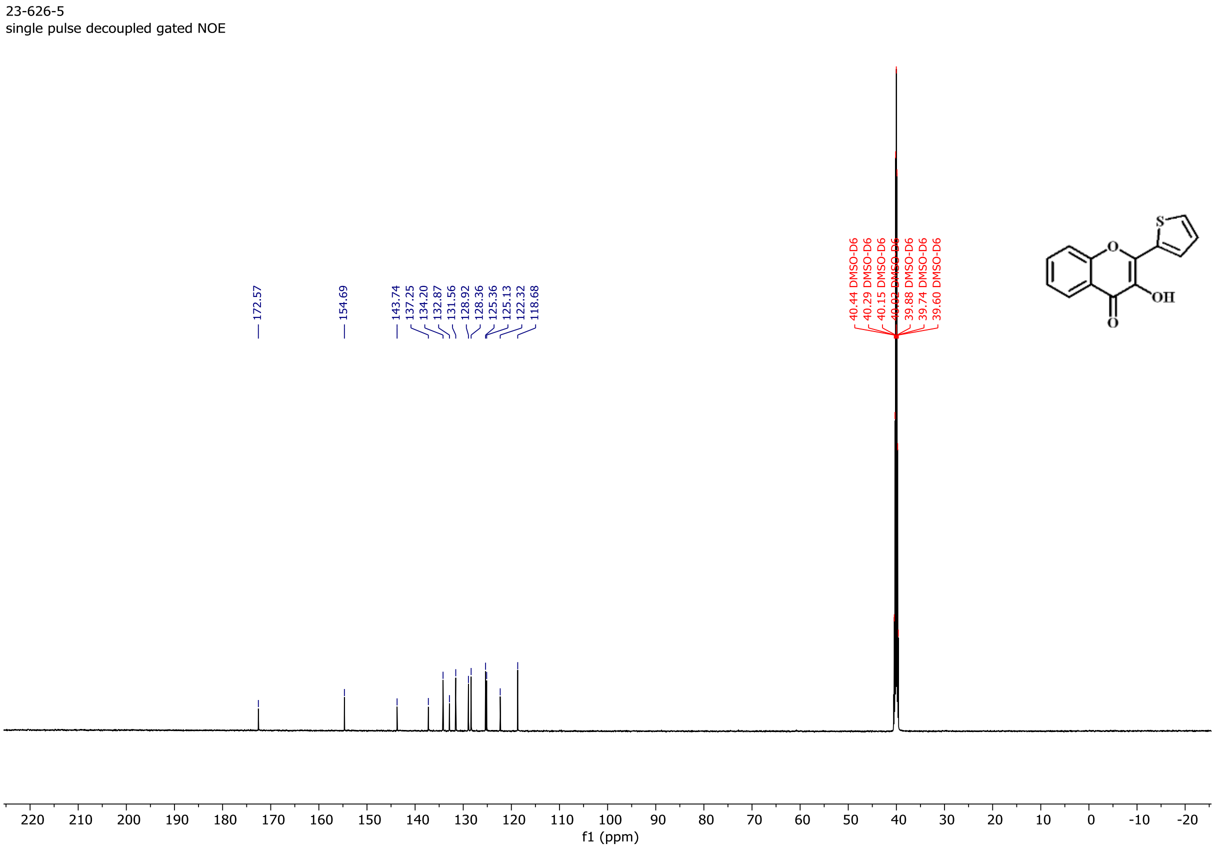

Figure S3. ^{13}C-NMR spectrum of compound **4d.**

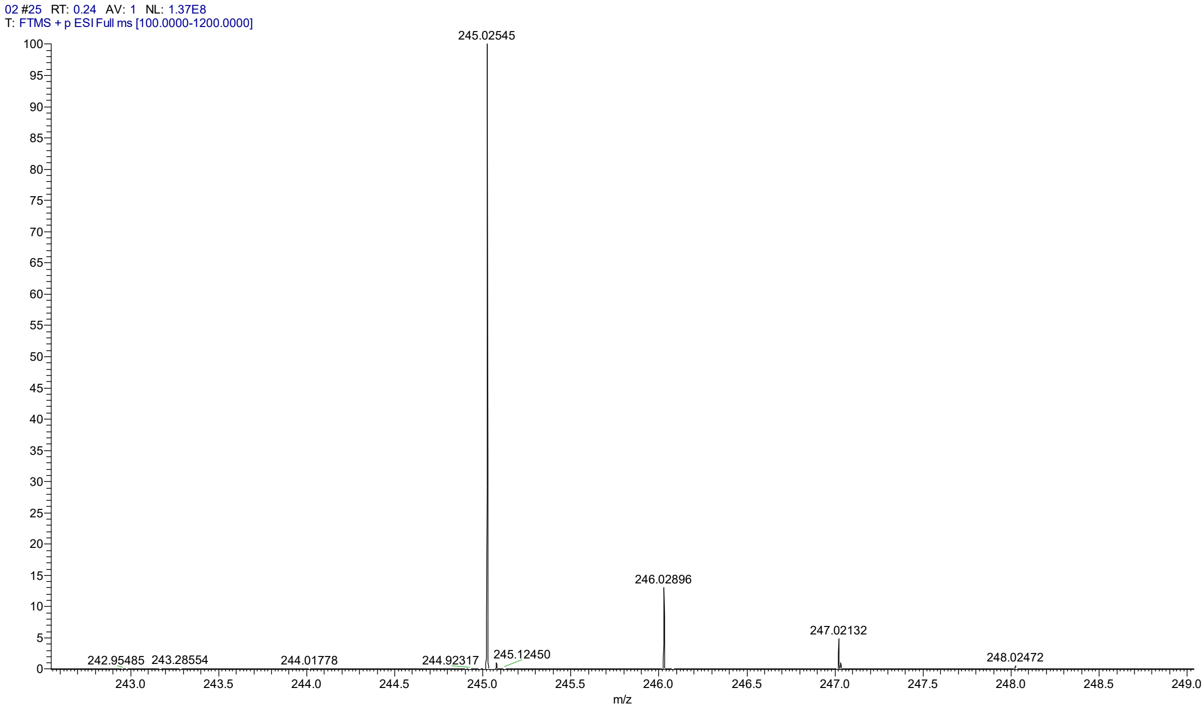

Figure S4. MS spectrum of compound **4d.**

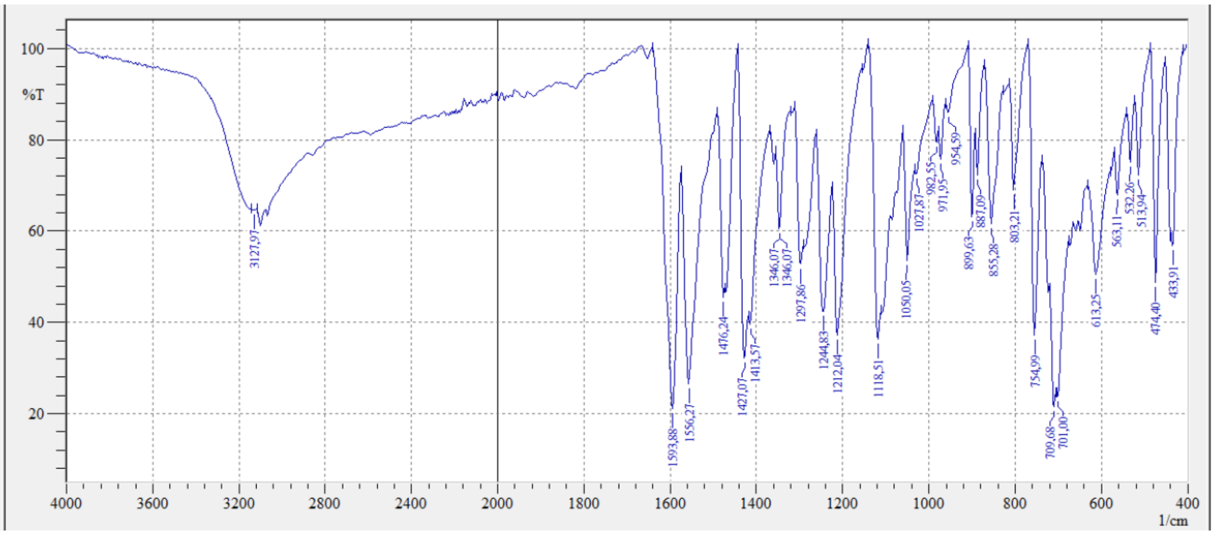

Figure S5. FTIR spectrum of compound **4d.**

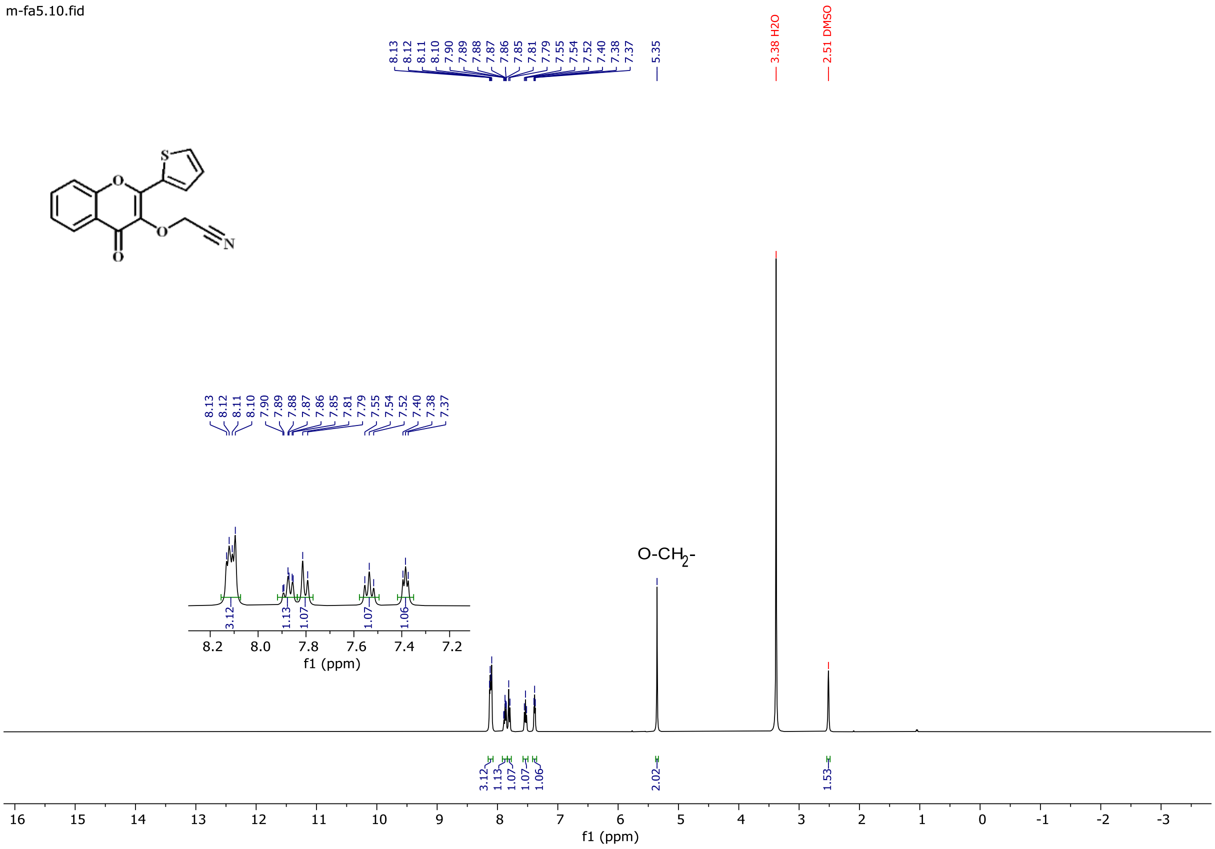

Figure S6. ^{1}H-NMR spectrum of compound **5d.**

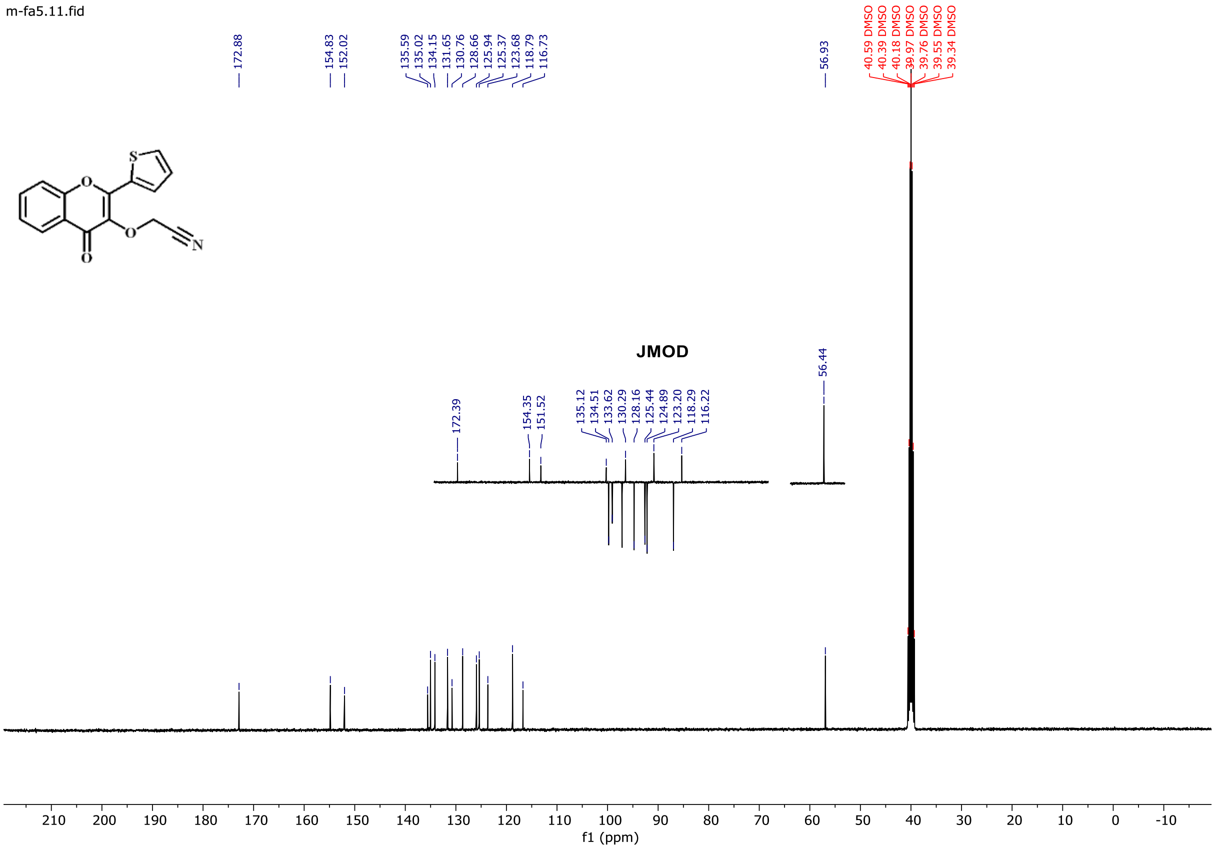

Figure S7. ^{13}C-NMR spectrum of compound **5d.**

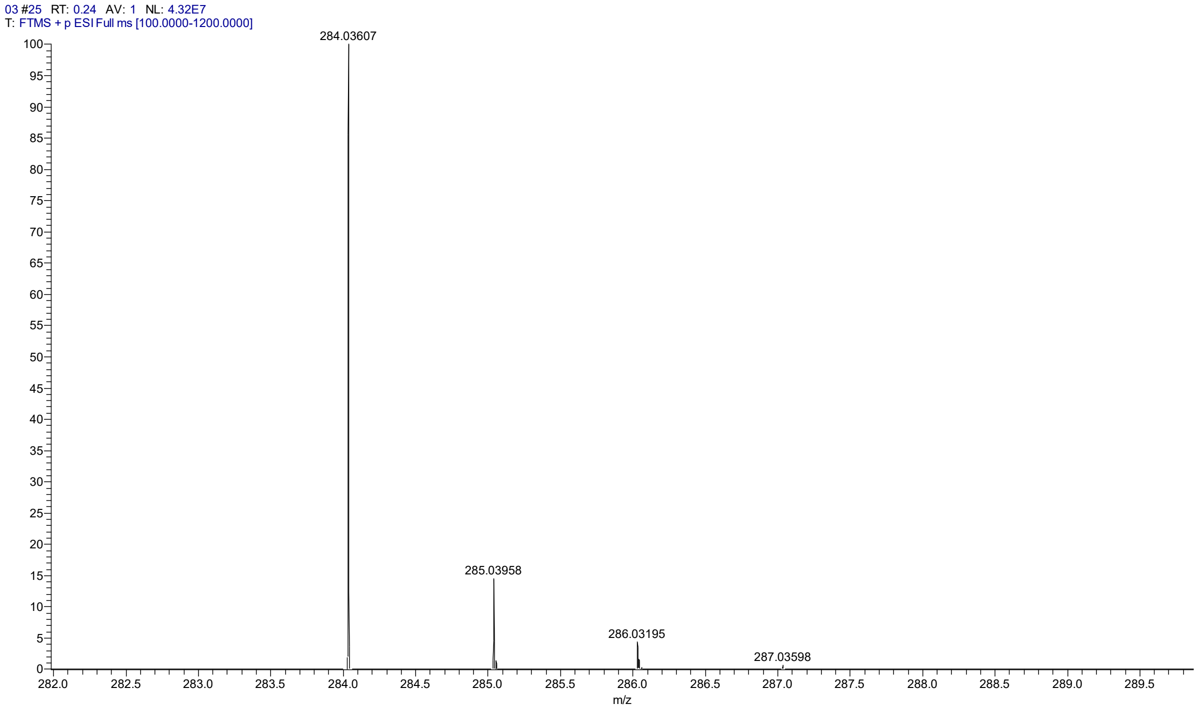

Figure S8. MS spectrum of compound **5d.**

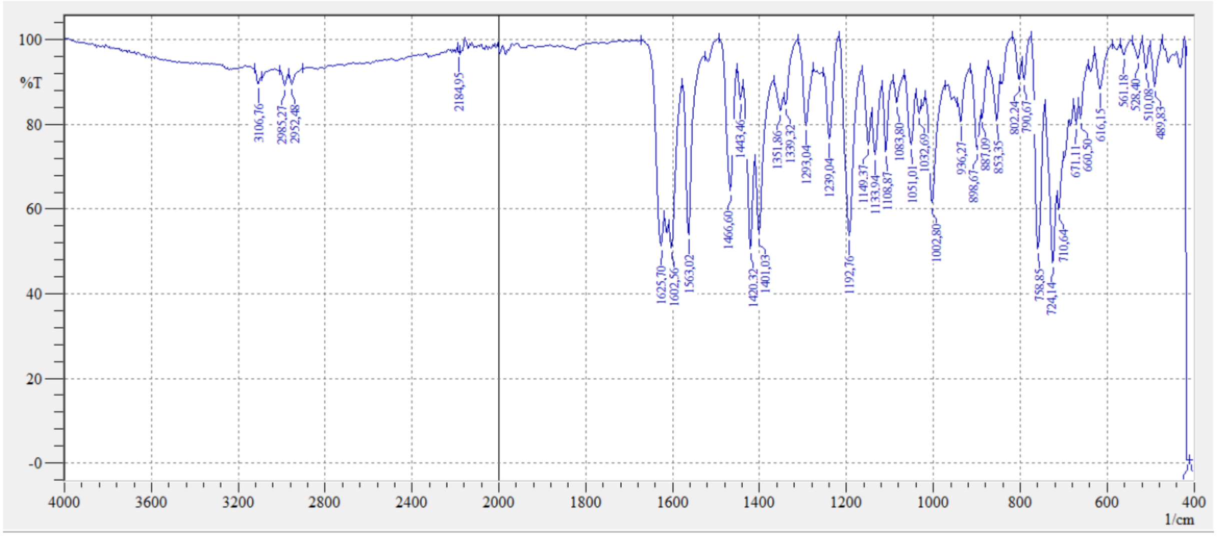

Figure S9. FTIR spectrum of compound **5d.**

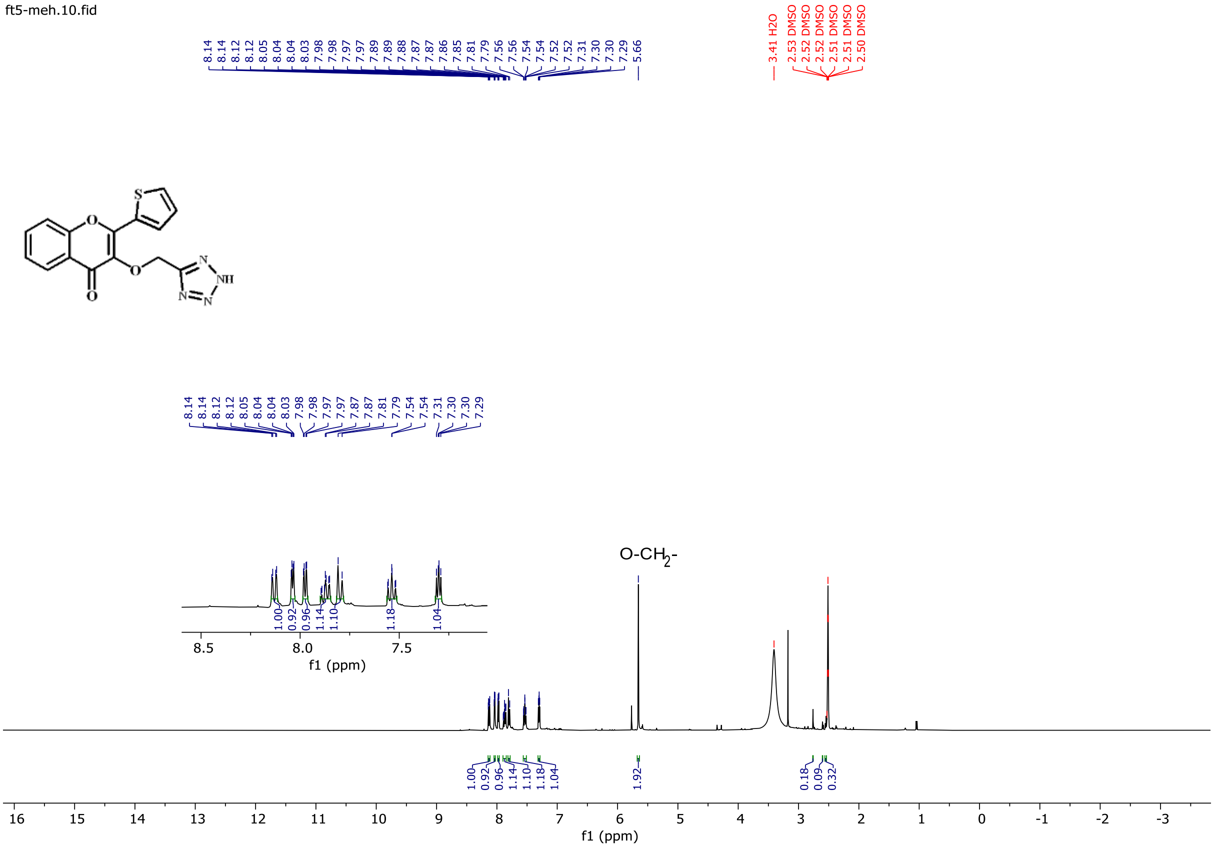

Figure S10. ^{1}H-NMR spectrum of compound **6d.**

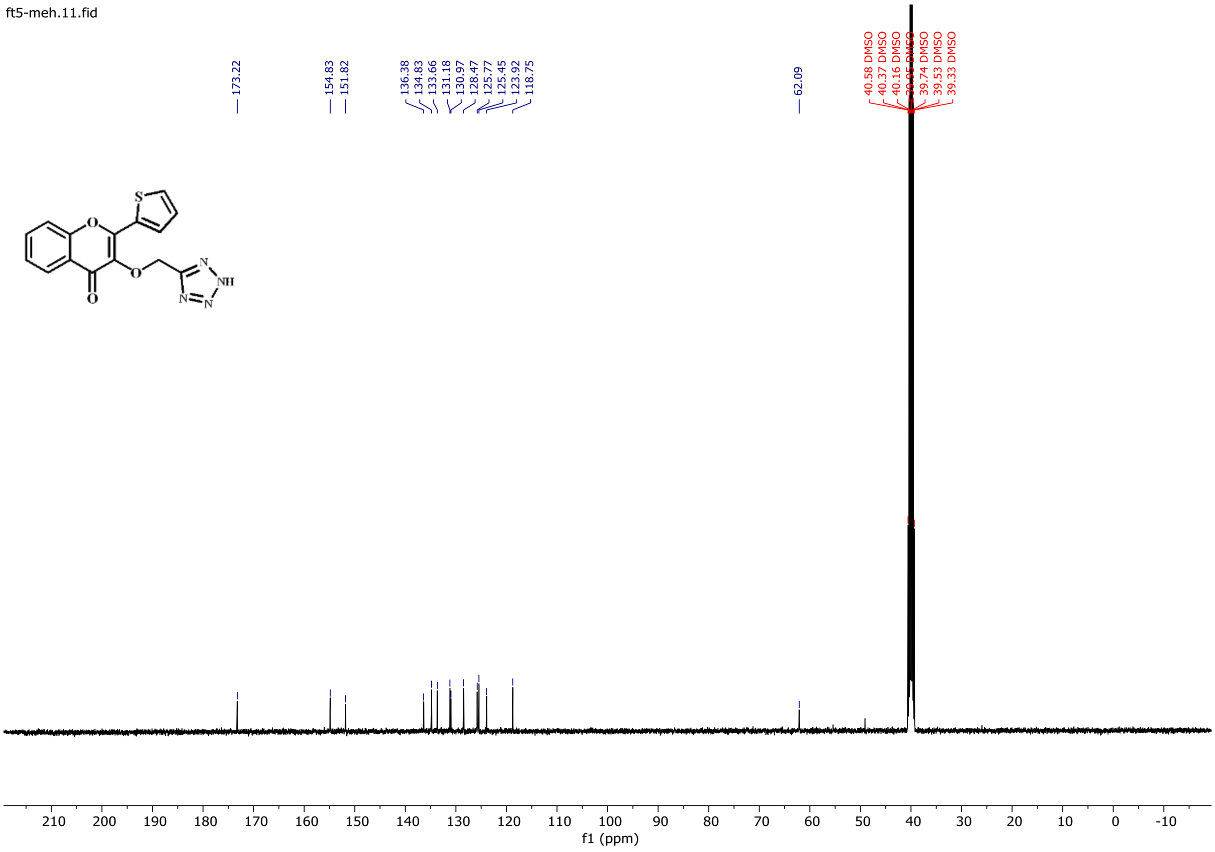

Figure S11. ^{13}C-NMR spectrum of compound **6d.**

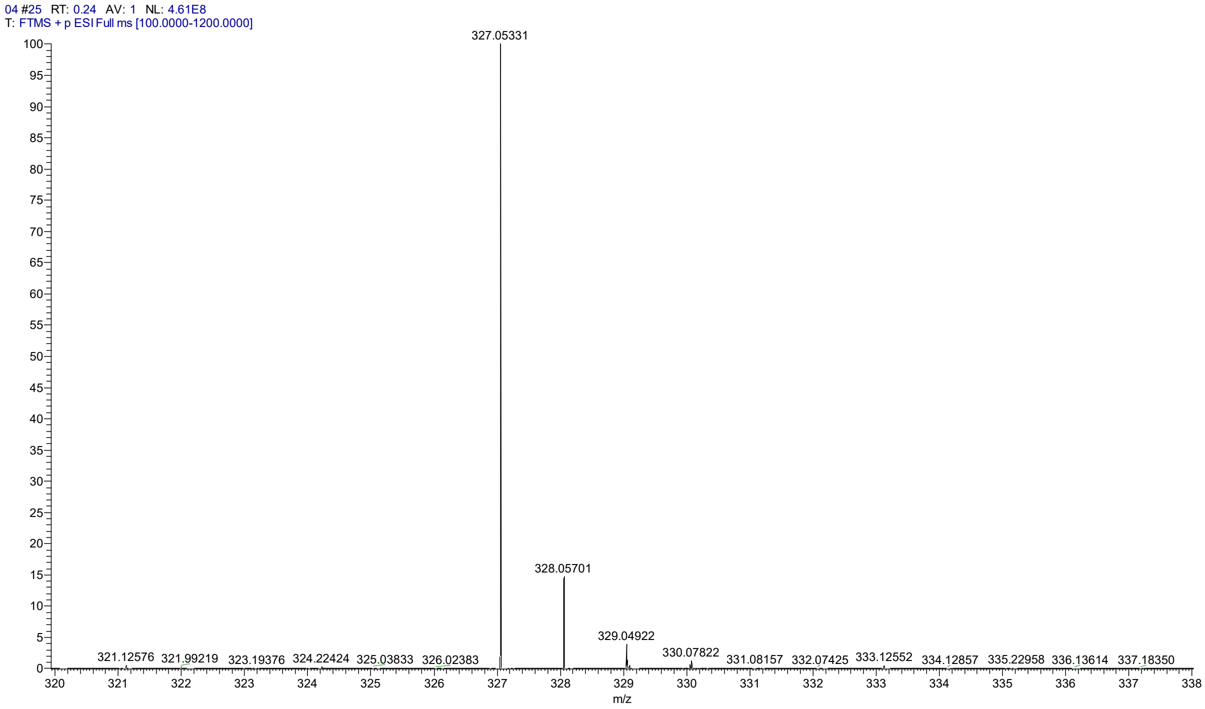

Figure S12. MS spectrum of compound **6d.**

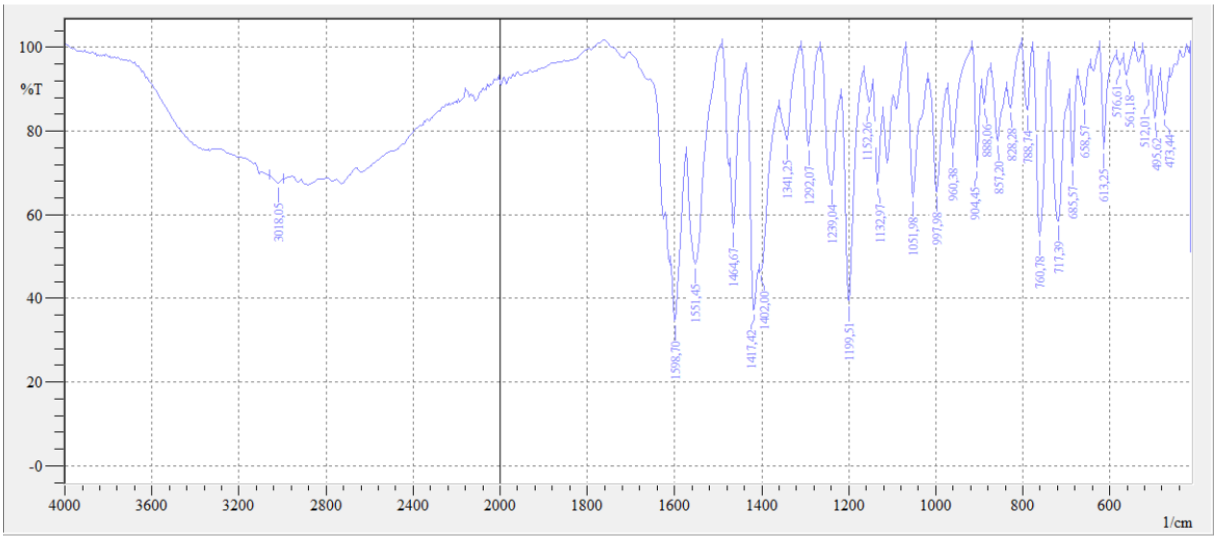

Figure S13. FTIR spectrum of compound **6d.**

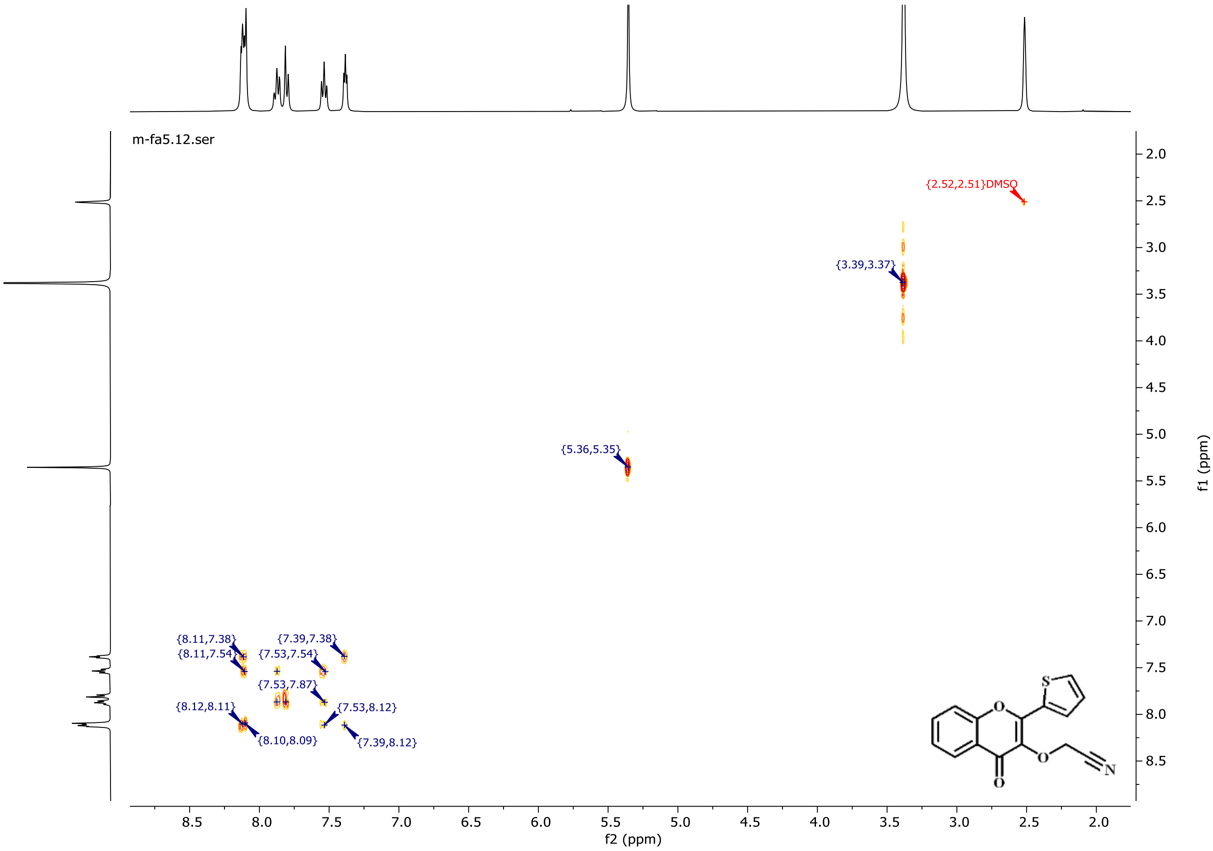

Figure S14. COSY spectrum of compound **5d.**

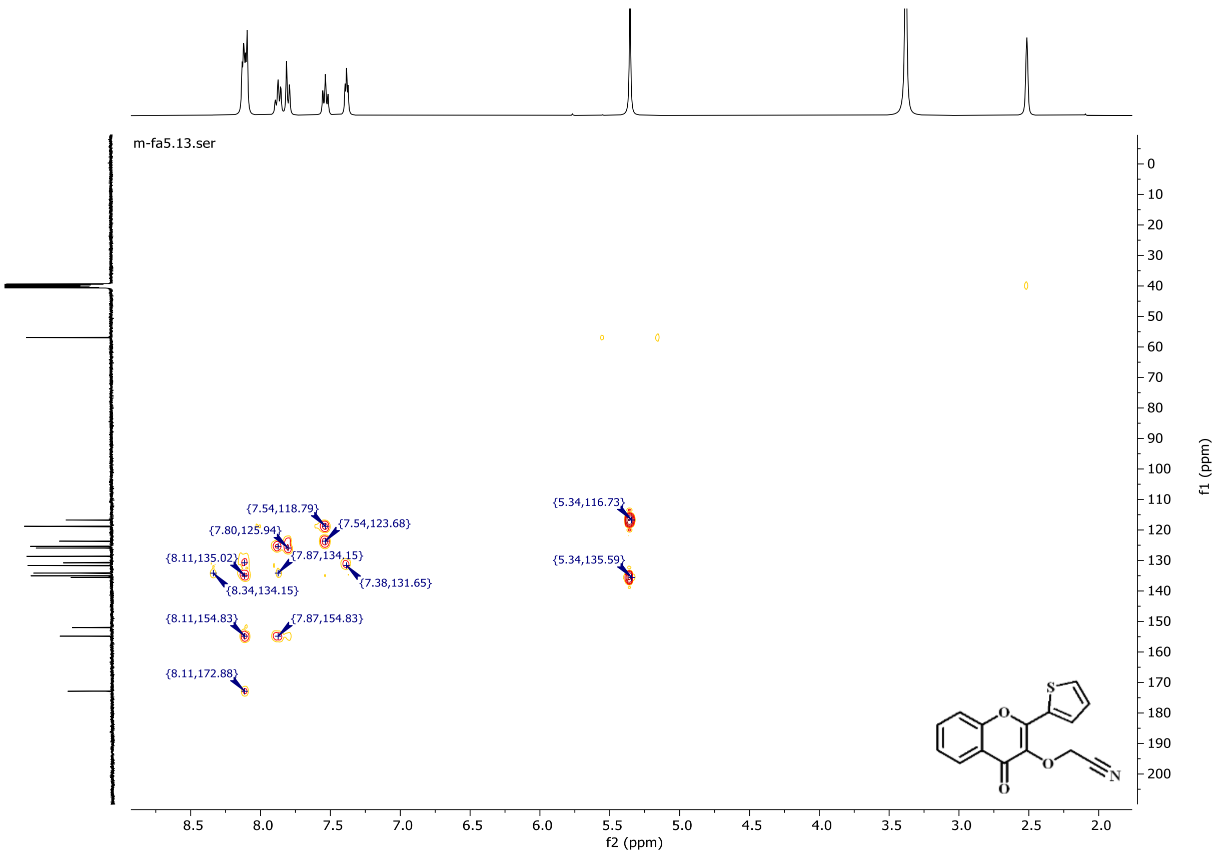

Figure S15. HMBC spectrum of compound **5d.**

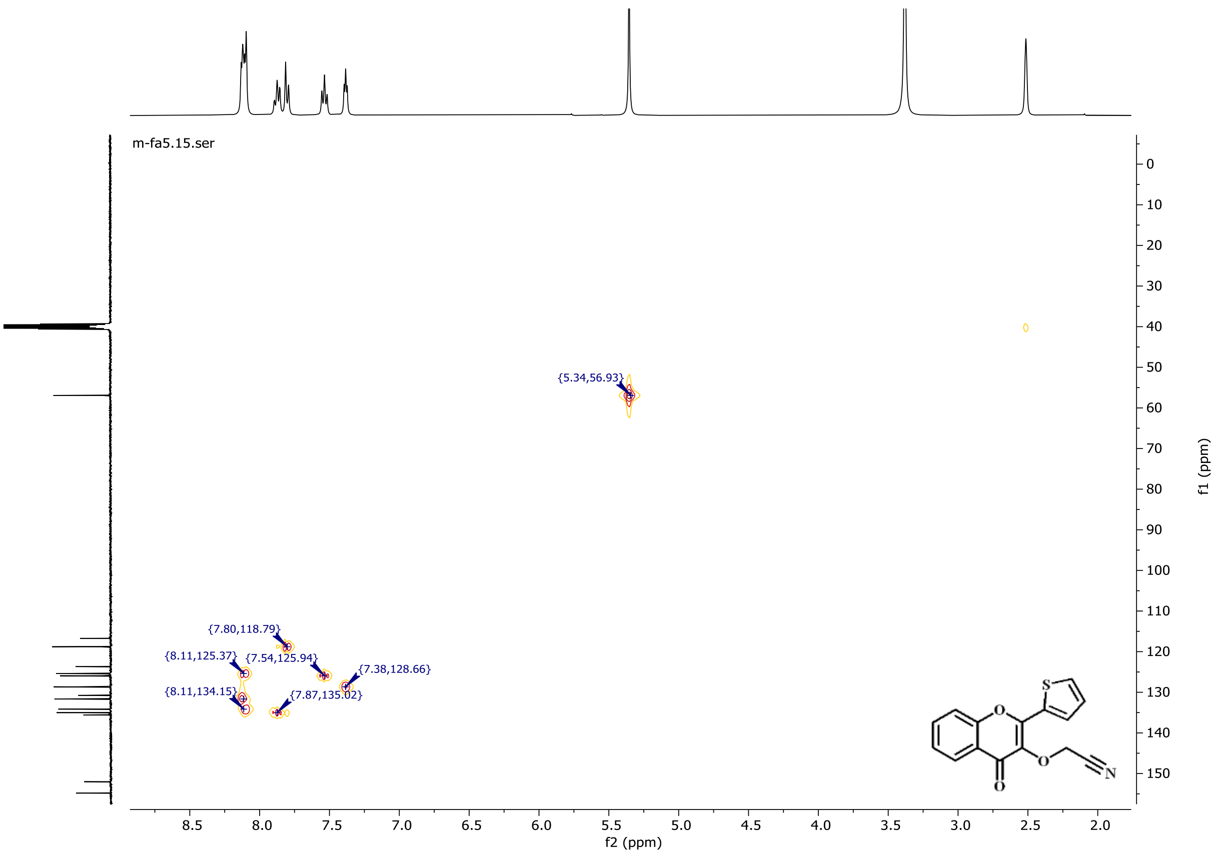

Figure S16. HMQC spectrum of compound **5d.**

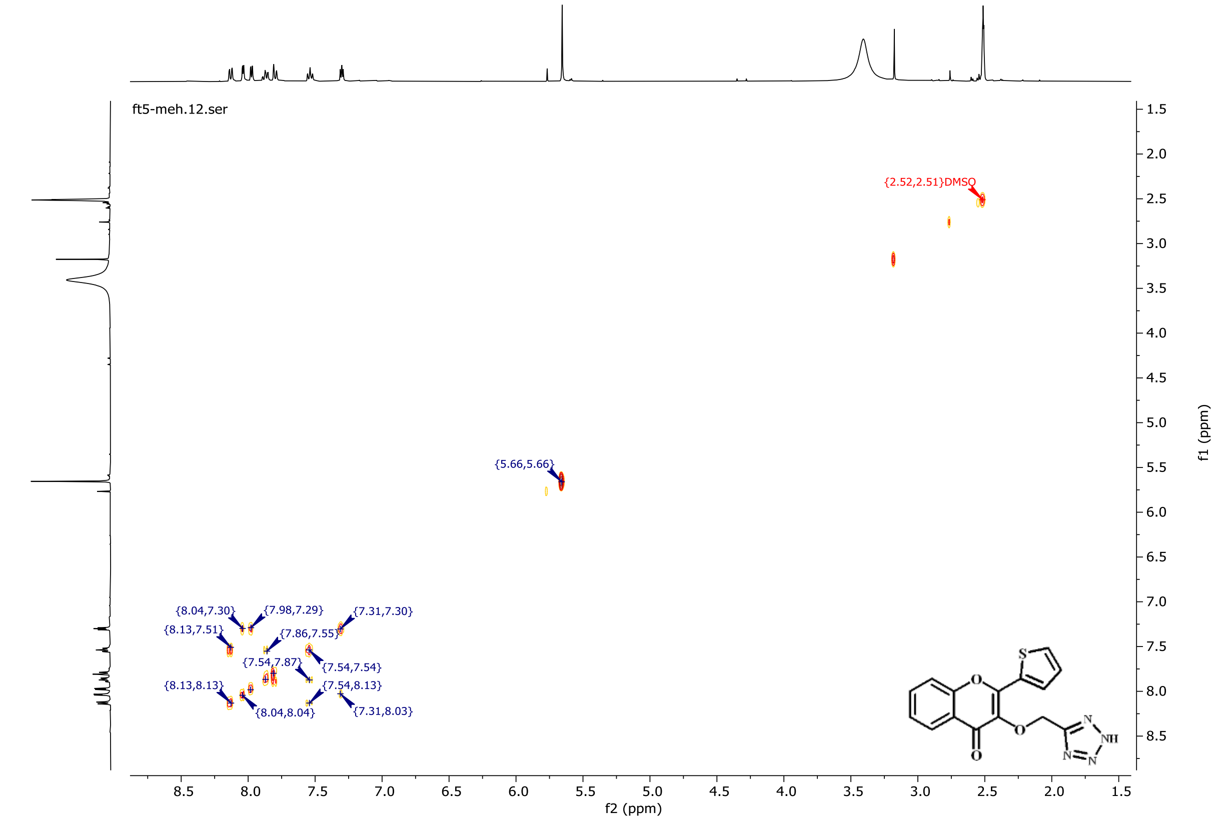

Figure S17. COSY spectrum of compound **6d.**

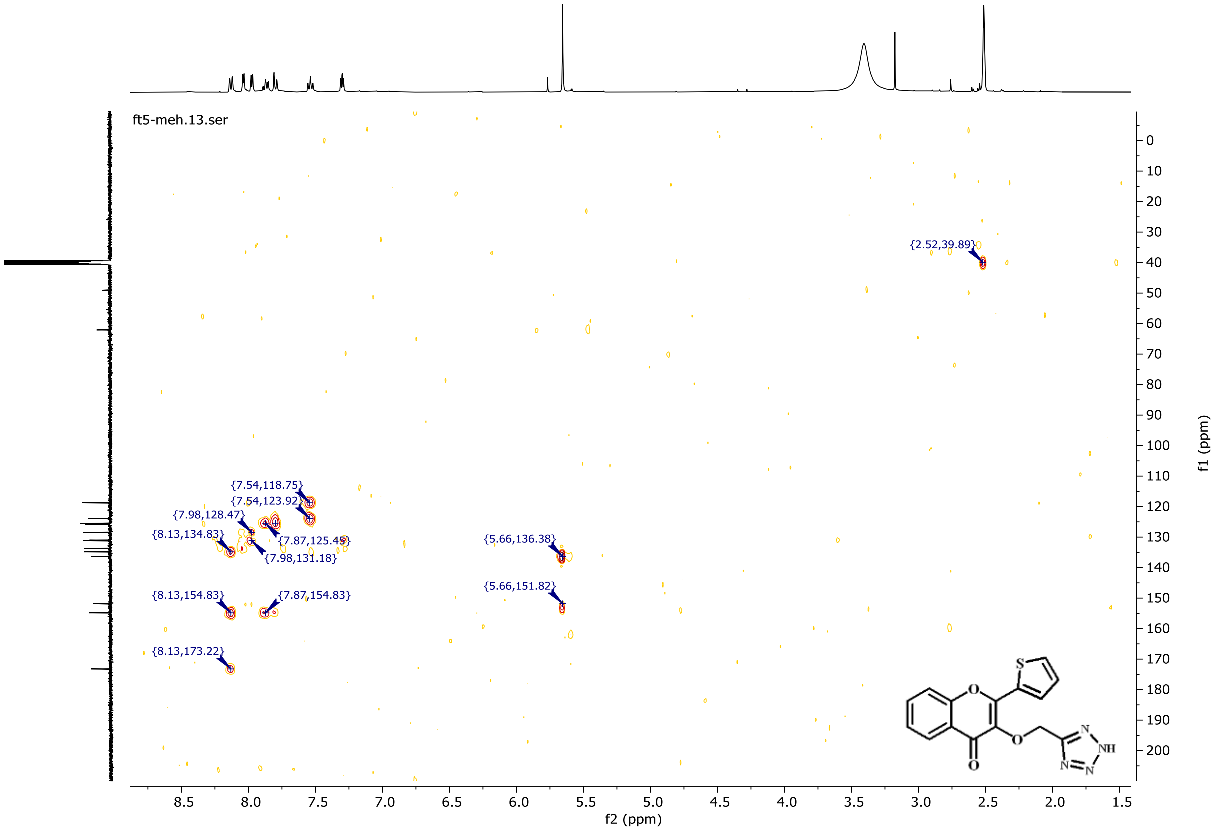

Figure S18. HMBC spectrum of compound **6d.**

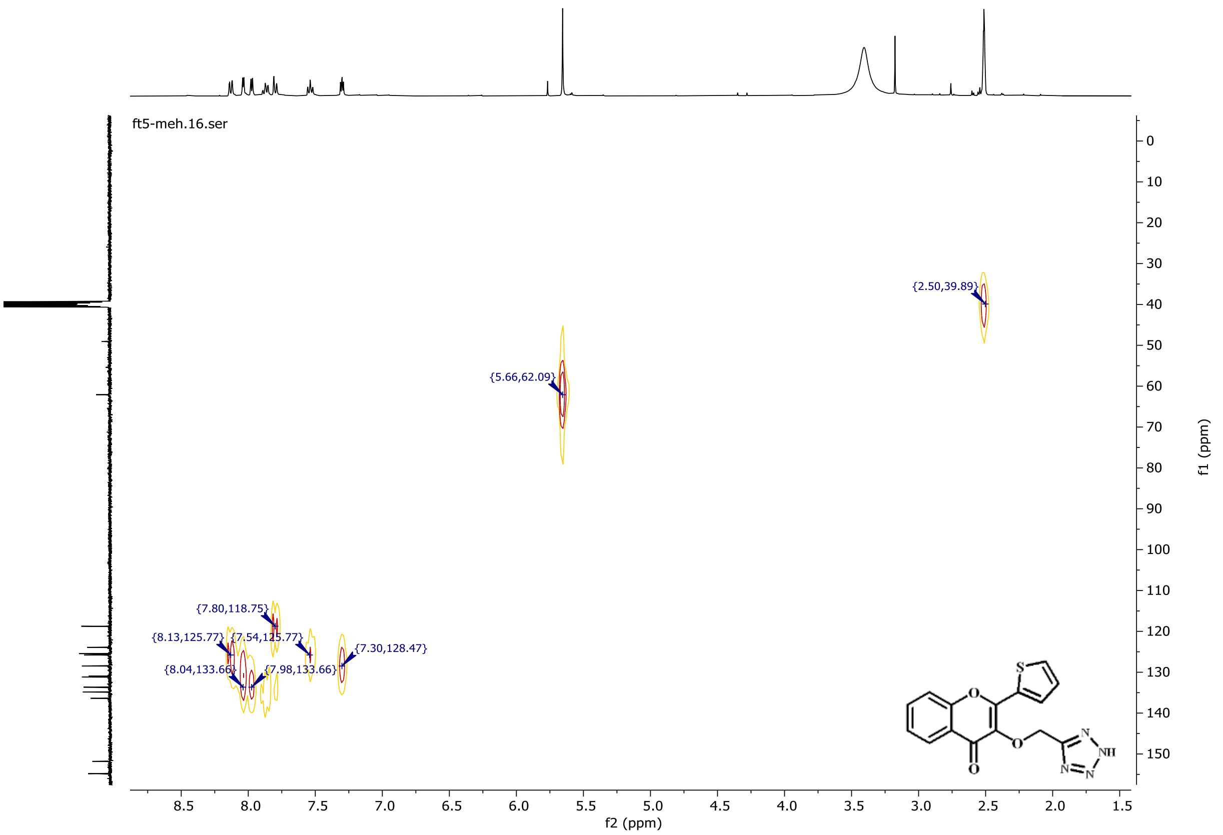

Figure S19. HMQC spectrum of compound **6d**

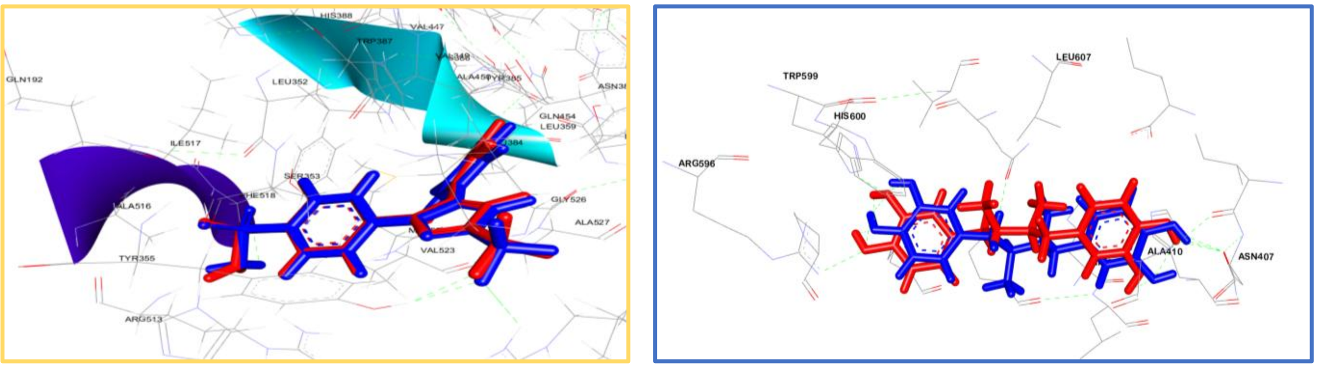

Cyclooxygenase-2 (PDB: *1CX2*)

RMSD= 0,27Å

5-lipooxygenase (PDB: *6N2W*)

RMSD= 1,9Å

Figure S20. Interactions of the active site residues with native cocrystal (Red) in the complex and the predicted docking pose of cocrystal (Bleu).

Table S1. The binding affinities and the contributing binding residues of COX-2 and 5-LOX with compounds **4-6**.

COX2 (PDB: 1CX2; RMSD= 0.27)					
**Compounds**	**IC50 (µg/mL)**	**Docking score (Kcal/mol)**	**Contributing binding residues**	**Hydrogen bonds**	**Distance (Å)**
**4a**	29,077	-7.897	VAL349, LEU352, VAL523, GLY526, ALA527	-	-
**4b**	24.85	-8.198	HIS90, GLN192, VAL349, LEU352, SER353, ALA516, VAL523, GLY526, ALA527	HIS90	2.16
**4c**	83.21	-7.134	VAL349, LEU352, SER353, TYR385, VAL523, GLY526, ALA527, LEU531	-	-
**4d**	51.67	-7.465	VAL349, LEU352, SER353, TRP387, VAL523, GLY526, ALA527, LEU531	-	-
**5a**	9.88	-8.827	ARG120, VAL349, LEU352, SER353, GLY526, ALA527, LEU531	-	-
**5b**	14.26	-8.247	HIS90, GLN192, LEU352, SER353,ALA516, VAL523, ALA527	-	-
**5c**	73.99	-7.277	VAL349, LEU352, VAL523, GLY526, ALA527, SER530	-	-
**5d**	136.790	-7.560	VAL349, LEU352, GLY526, ALA527, LEU531	-	-
**6a**	37.60	-6.754	HIS90, ARG120, VAL349, LEU352, SER353, ALA516, PHE518, VAL523, GLY526, ALA527, LEU531	HIS90<br>LEU352	2.72<br>2.62
**6b**	53.33	-6.941	HIS90, VAL116, GLN192, VAL349, LEU352, SER353, TYR355, LEU359 ALA516 VAL523, GLY526, ALA527, LEU531	HIS90<br>TYR355	2.25<br>2.15
**6c**	64.75	-6,196	VAL116, VAL349, LEU352, SER353, TYR355, VAL523, GLY526, ALA527, LEU531	TYR355	2.20
**6d**	35.190	-6,250	VAL116, VAL349, LEU352, TYR355, LEU359, ARG513, PHE518, VAL523, GLY526, ALA527, LEU531	TYR355	2.12
**S58**	-	-9.235	HIS90, ARG120, VAL349, LEU352, SER353, TYR355, PHE381, LEU384, TYR385, TRP387, PHE518, VAL523, GLY526, ALA527	HIS90<br>ARG120<br>TYR355<br>PHE518	2.79<br>2.10<br>2.11; 2.77<br>3.08

5-LOX (PDB: 6N2W; RMSD=1.9)					
**Compounds**	**IC50 (µg/mL)**	**Docking score (Kcal/mol)**	**Contributing binding residues**	**Hydrogen bonds**	**Distance (Å)**
**4a**	38.11	-5.238	PHE359, LEU368, HIS432, ARG596, TRP599, ALA603	ARG596	6.19
**4b**	47.69	-4.272	PHE359, LEU414, HIS432, ARG596, TRP599, ALA603	ARG596	6.05
**4c**	140.55	-4.211	PHE359, THR364, ARG596, TRP599, ALA603	ARG596	2.59
**4d**	57.23	-5.048	PHE359, HIS432, ARG596, ALA603	HIS432<br>ARG596	2.77<br>2.62
**5a**	38.11	-5.359	PHE359, HIS360, THR364, PRO569, ARG596, TRP599, ALA603	THR364 ARG596	3.02<br>5.94
**5b**	47.69	-5.190	PHE359, HIS360, LEU414, HIS432, ARG596, TRP599, ALA603	ARG596	6.14
**5c**	140.55	-5.000	PHE359, HIS360, THR364, HIS432, PRO569, ARG596, TRP599, ALA603	ARG596	2.61
**5d**	59.56	-4.937	PHE359, HIS360, THR364, HIS432, ARG596, ALA603	ARG596	2.77
**6a**	45.68	-4.660	PHE359, ARG596, ALA603	ARG596	5.73; 2.60; 2.13
**6b**	20.10	-5.734	PHE359, GLN363, HIS367, LEU368, HIS372, LEU414 HIS432, TRP599, ALA603	-	-
**6c**	44.68	-4.681	PHE359, GLN363, LEU368, LEU414, HIS432, ARG596, ALA603	GLN363<br>ARG596	3.28<br>2.79
**6d**	50.55	-5.059	PHE359, HIS432, ARG596, TRP599, ALA603	ARG596	2.64
**NDGA**	-	-7.656	HIS367, ASN407, ALA410, ARG596, TRP599, HIS600, LEU607	ARG596 HIS600	2.51<br>2.31; 2.57

Table S2. *In silico* Lipinski's rule of five and Predicted ADMET analysis for selected derivatives **4a-b**, **5a-b**, and **6a-b**.

**Compounds**	**4a**	**4b**	**5a**	**5b**	**6a**	**6b**	**Celecoxib**	**Zileuton**
**Molecular weight**	238.242	268.268	277.279	307.305	320.307	350.333	381.372	236.288
**Donors HB**	1	1	0	0	1	1	2	3
**Acceptors HB**	3.250	4.000	4.750	5.500	6.250	7.000	5.7	3.7
**QPlogPo/W**	2.594	2.710	2.485	2.558	2.003	2.129	3.321	0.927
**Rule of five**	0	0	0	0	0	0	0	0
**SASA**	472.135	509.431	523.072	561.206	521.226	544.405	620.510	444.343
**FOSA**	0.000	92.864	36.804	130.357	43.043	130.594	88.768	81.922
**FISA**	93.125	93.089	111.708	111.179	140.649	131.021	152.930	132.926
**PISA**	379.010	323.477	374.561	319.670	337.535	282.791	252.965	194.893
**Volume**	778.239	853.841	894.872	971.342	934.607	1009.623	1077.549	747.797
**QPPCaco**	1296.566	1297.581	864.123	874.149	459.331	566.798	351.289	325.689
**QPlogBB**	-0.323	-0.407	-0.628	-0.715	-0.809	-0.770	-0.768	-0.635
**QPlogS**	-3.586	-4.011	-4.154	-4.321	-3.124	-3.101	-5.769	-1.674
**QPPMDCK**	655.040	655.594	422.470	427.770	213.377	267.815	781.010	396.140
**QPlogHERG**	-5.497	-5.431	-5.607	-5.561	-4.971	-4.595	-5.664	-2.759
**%Human Oral Absorption**	100	100	94.058	94.575	86.318	88.692	91.951	77.345

SASA: Total solvent accessible surface area. Range 300.0 - 1000.0

FOSA: Hydrophobic component of the SASA. Range 0.0 - 750.0

FISA: Hydrophilic component of the SASA. Range 7.0 - 330.0

PISA: π (carbon and attached hydrogen) component of the SASA. Range 0.0 - 450.0

Volume: Total solvent-accessible volume in cubic angstroms. Range 500.0 - 2000.0

QPPCaco: Predicted apparent Caco-2 cell permeability. Range 25 -500

QPlogBB: Predicted brain/blood partition coefficient. Range -3.0 - 1.2

QPlogHERG: Predicted IC50 value for blockage of HERG K+ channel concern below -5

RuleOfFive Number of violations of Lipinski's rule of five. The rules are: mol_MW < 500, QPlogPo/w < 5, donorHB ≤ 5, accptHB ≤ 10

Table S3. Predicted metabolism, exertion, and toxicity parameters of flavones derivatives **4a-b**, **5a-b**, and **6a-b**using pkCSM

	Compounds	4a	4b	5a	5b	6a	6b	Celecoxib	Zileuton
Metabolism	CYP2D6 substrate	No	No	No	No	No	No	No	No
	CYP3A4 substrate	Yes	Yes	Yes	Yes	Yes	Yes	Yes	No
	CYP1A2 inhibitor	Yes	Yes	Yes	Yes	Yes	Yes	Yes	Yes
	CYP2C19 inhibitor	Yes	Yes	Yes	Yes	No	No	Yes	Yes
	CYP2C9 inhibitor	Yes	Yes	Yes	No	Yes	Yes	Yes	No
	CYP2D6 inhibitor	No	No	No	No	No	No	No	No
	CYP3A4 inhibitor	No	No	No	No	No	No	Yes	No
Excretion	Total Clearance (log ml/min/kg)	0.169	0.191	0.298	0.325	-0.106	-0.076	0.435	0.12
	Renal OCT2 substrate	No	Yes	Yes	Yes	No	No	No	No
Toxicity	AMES toxicity	No	No	No	No	No	No	No	No
	Max. tolerated dose (human) (log mg/kg/day)	0.089	0.01	-0.043	-0.194	0.596	0.580	0.178	0.812
	hERG I inhibitor	No	No	No	No	No	No	No	No
	hERG II inhibitor	No	No	No	No	No	No	No	No
	LD50 (mol/kg)	1.673	1.927	1.801	2.069	2.653	2.70	1.975	2.689
	LOAEL (log mg/kg_bw/day)	1.079	0.811	1.655	1.439	2.945	2.861	1.126	2.598

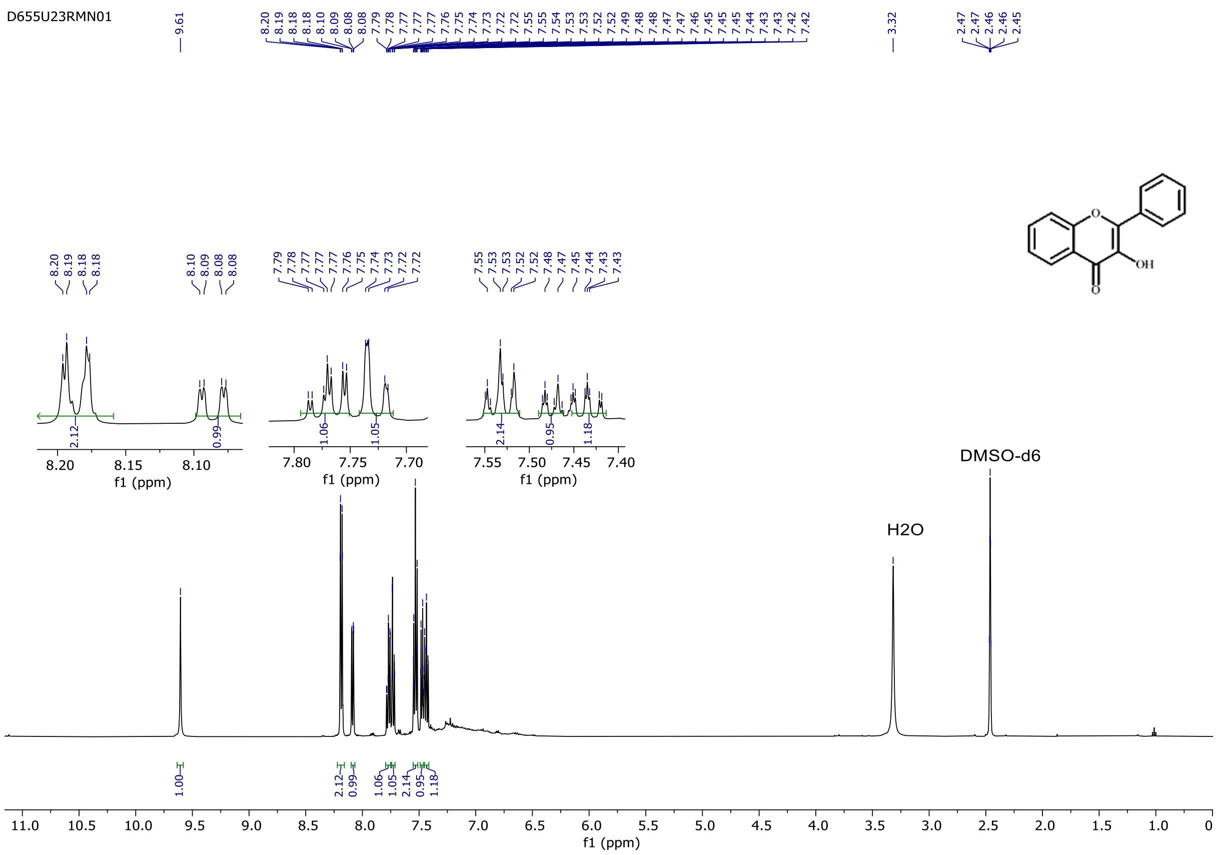

Figure S21. ^{1}H-NMR spectrum of compound **4a**

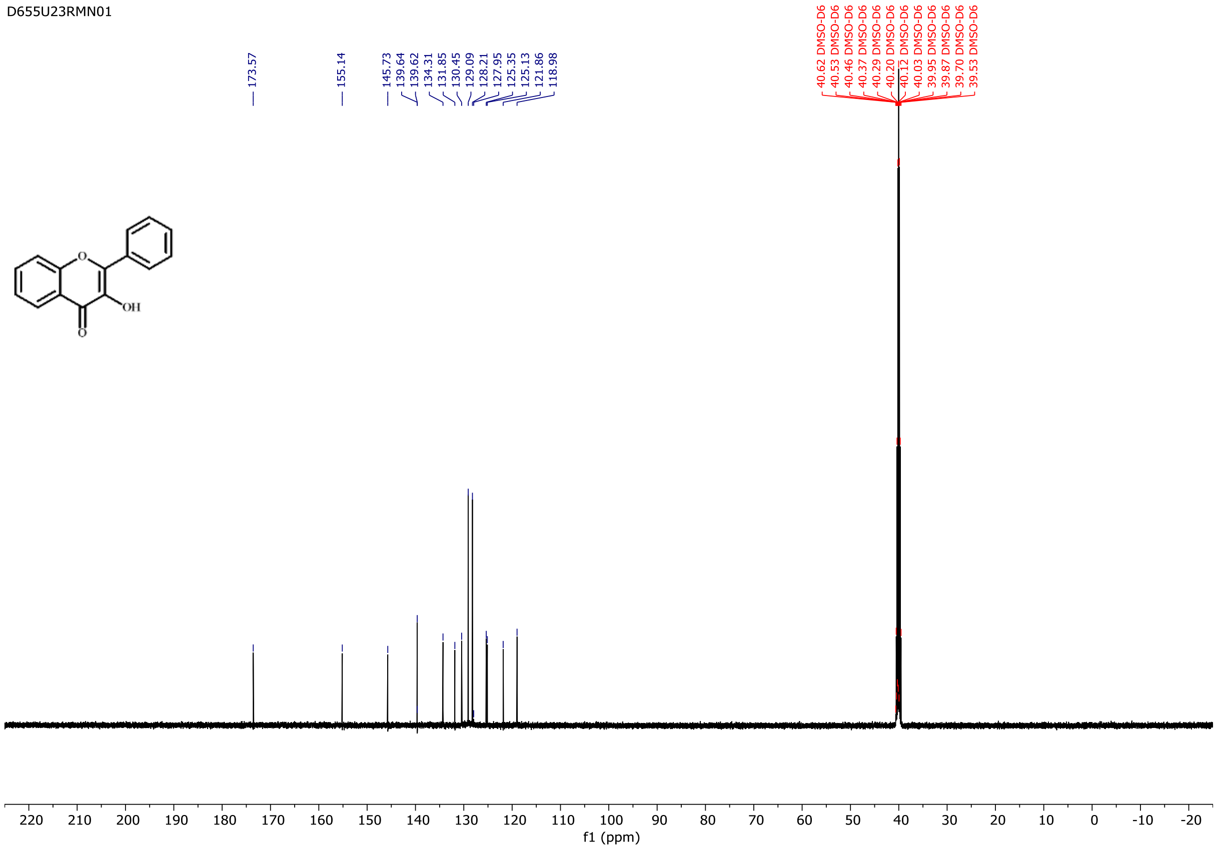

Figure S22. ^{13}C-NMR spectrum of compound **4a**

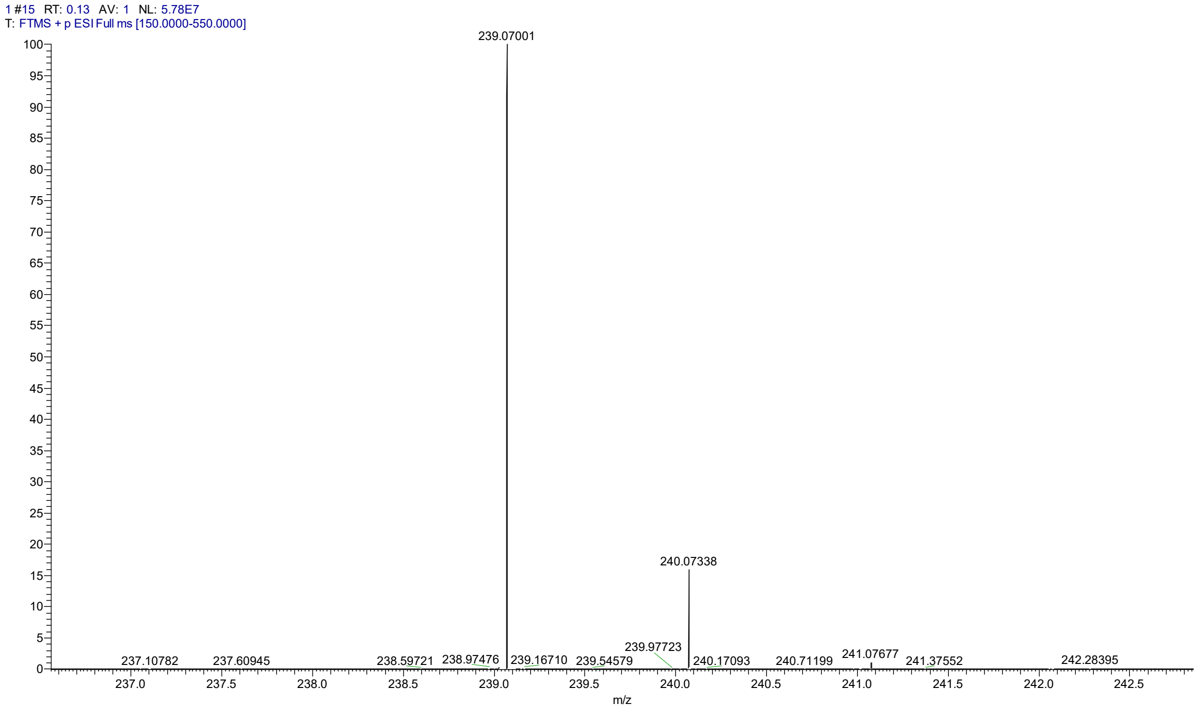

Figure S23. MS spectrum of compound **4a**

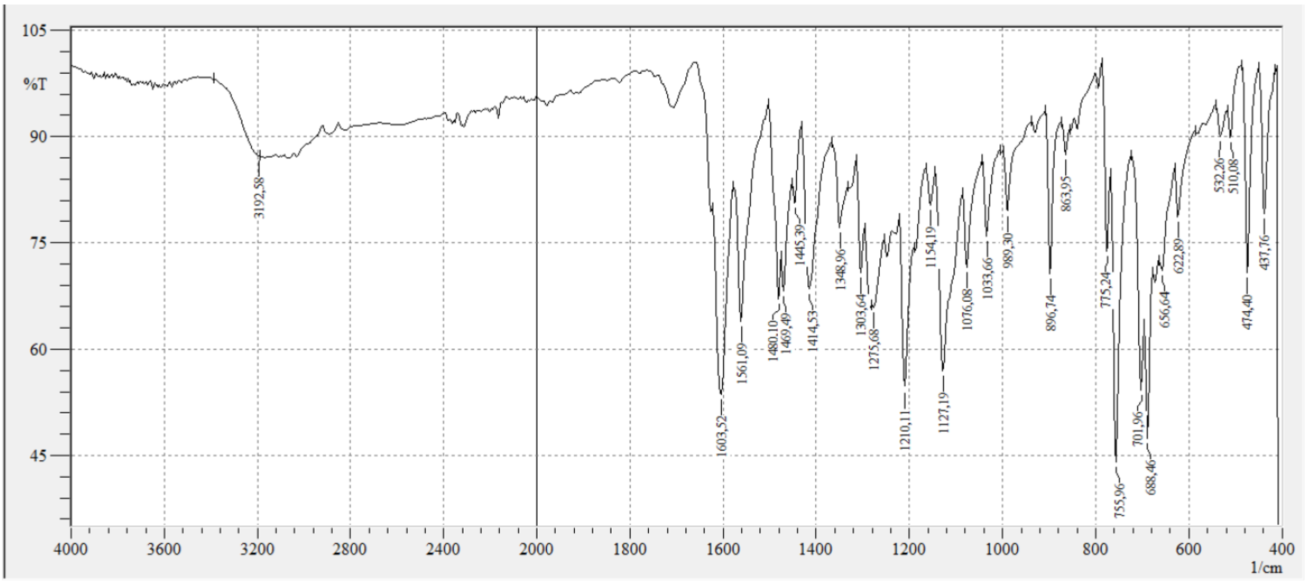

Figure S24. FTIR spectrum of compound **4a**

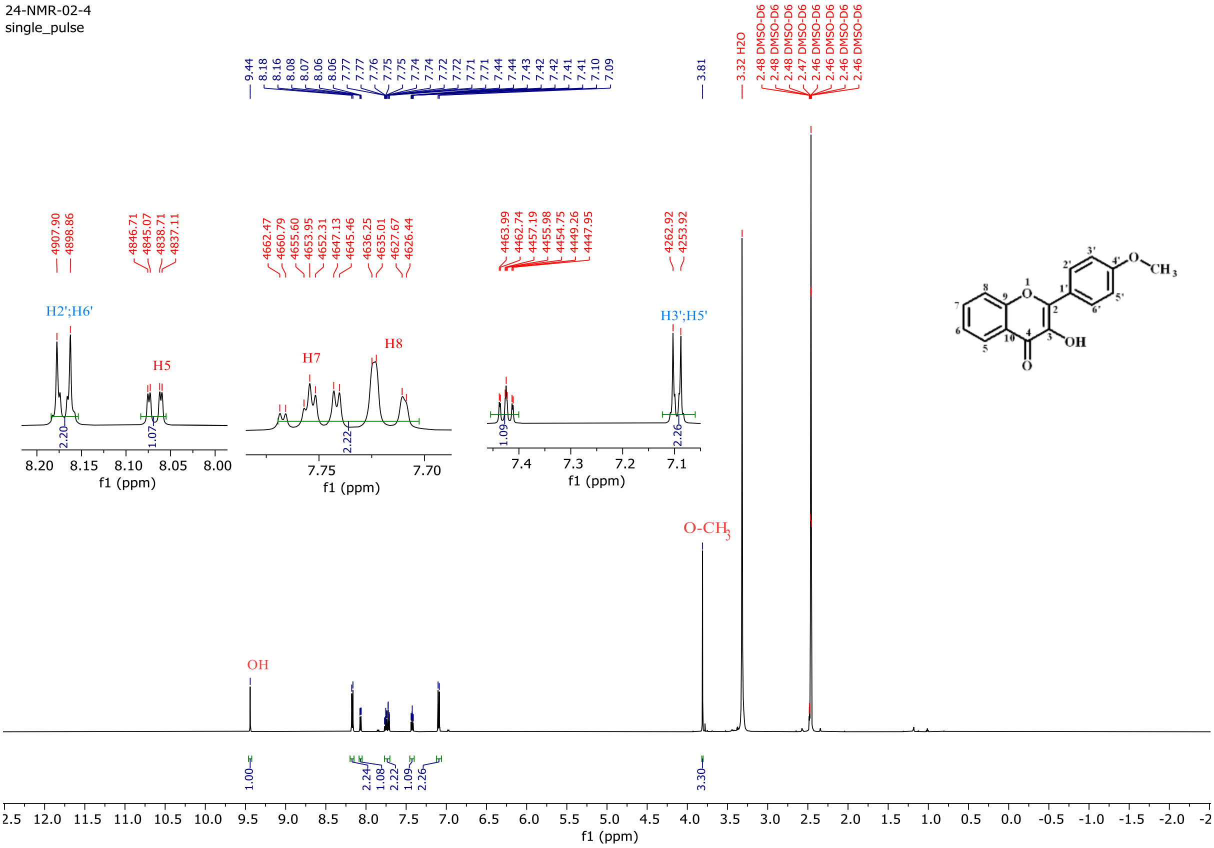

Figure S25. ^{1}H-NMR spectrum of compound **4b**

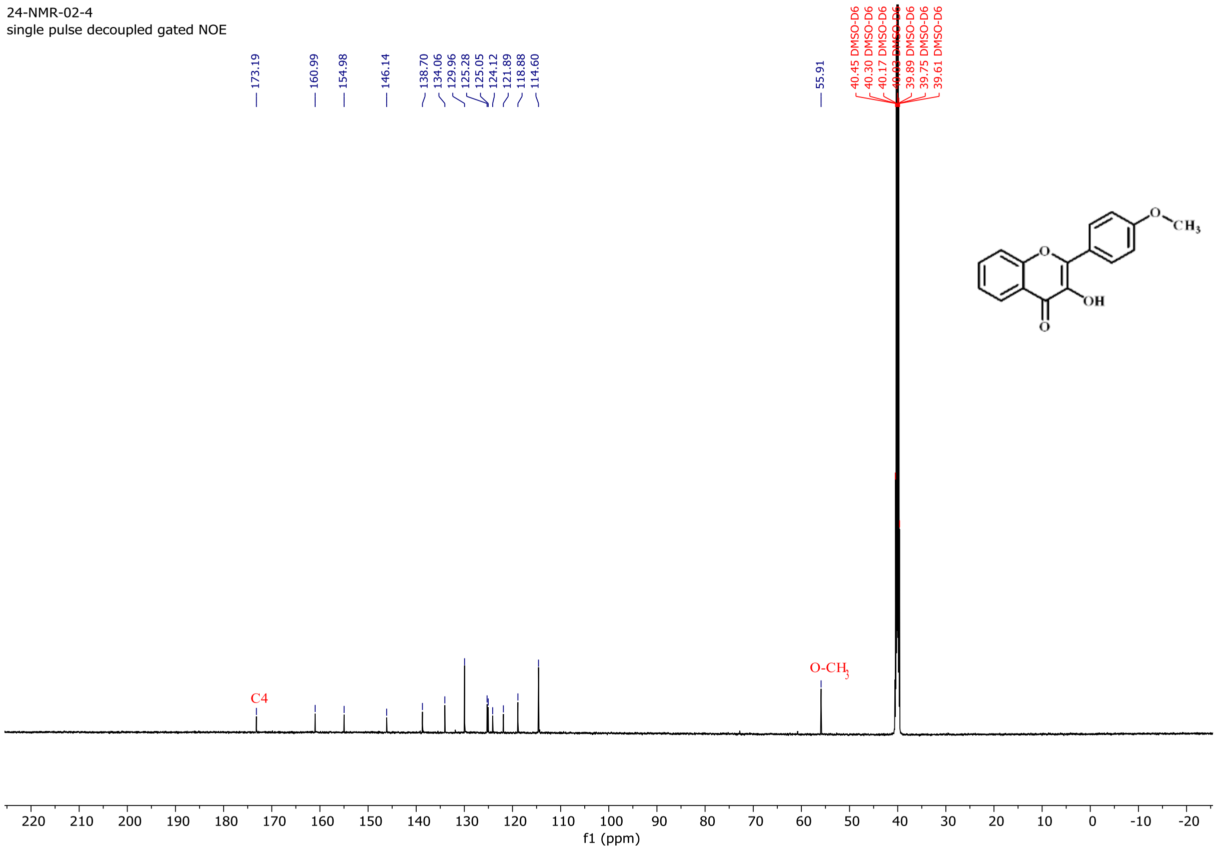

Figure S26. ^{13}C-NMR spectrum of compound **4b**

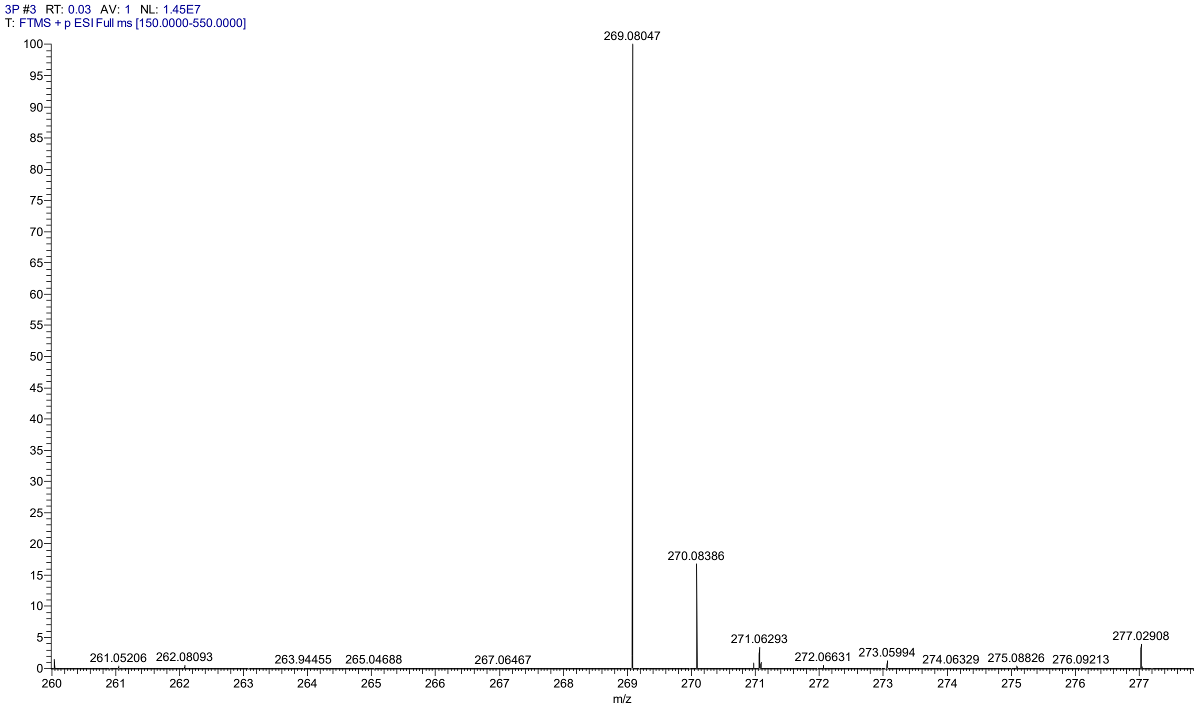

Figure S27. MS spectrum of compound **4b**

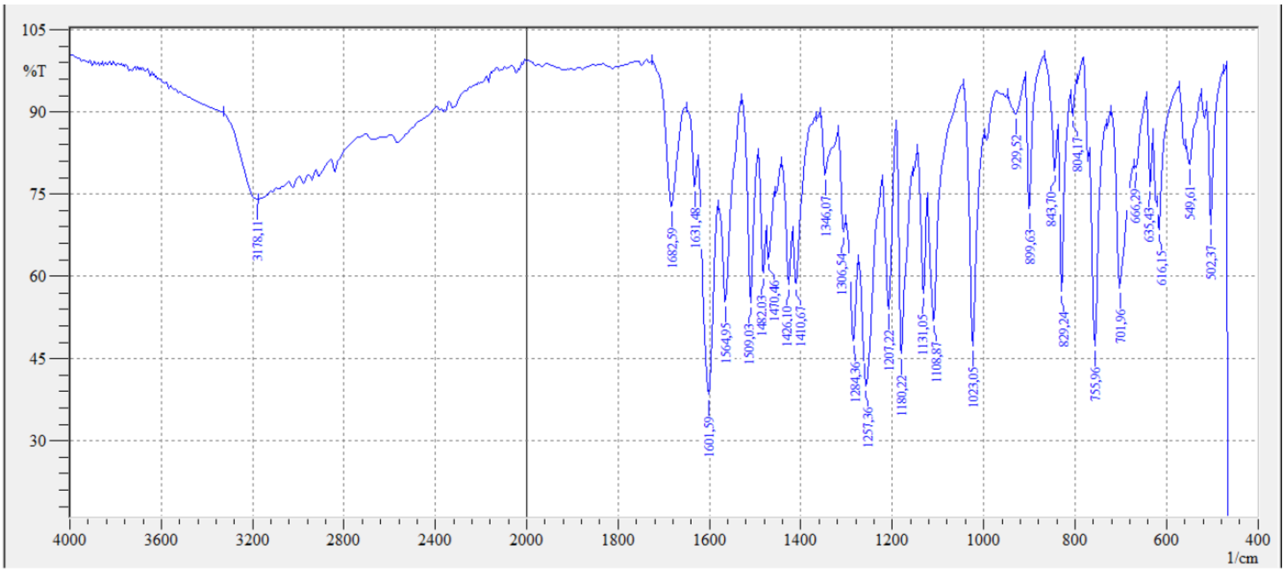

Figure S28. FTIR spectrum of compound **4b**

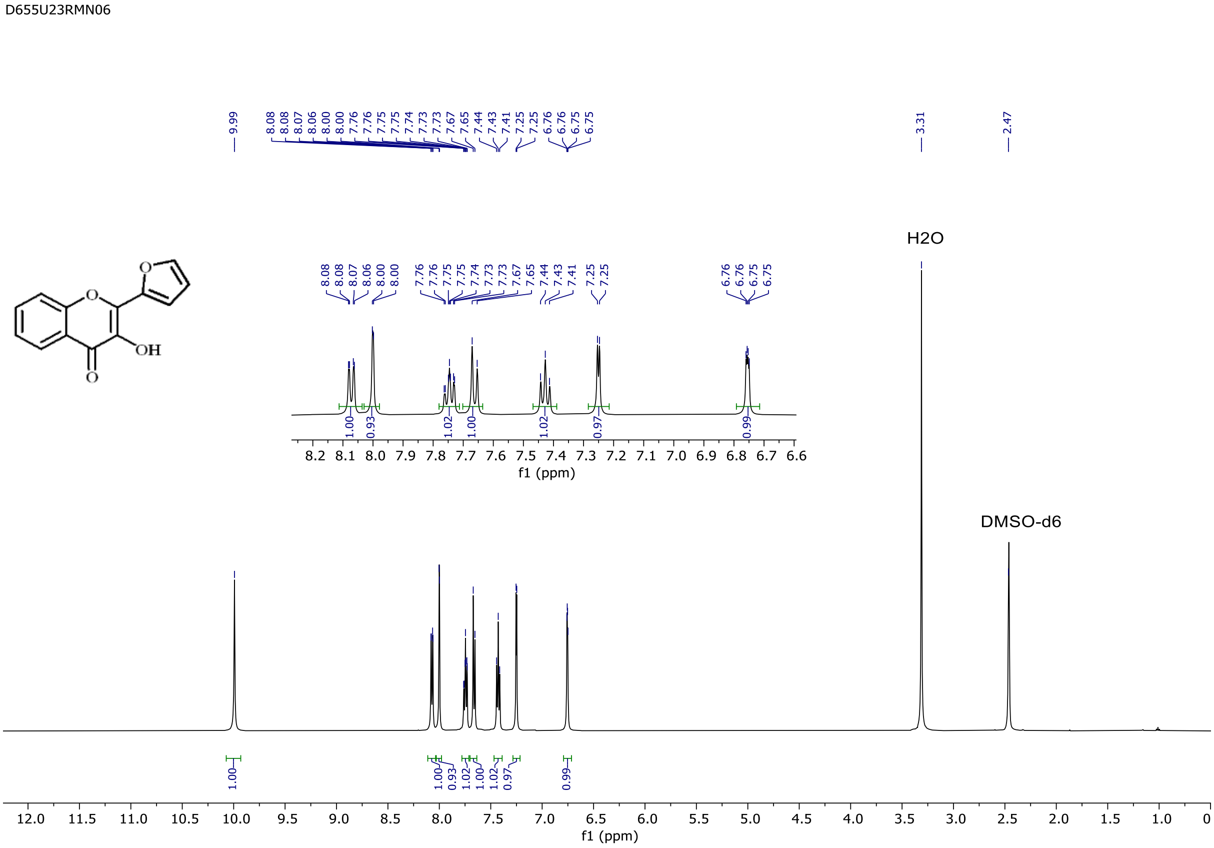

Figure S29. ^{1}H NMR spectrum of compound **4c**

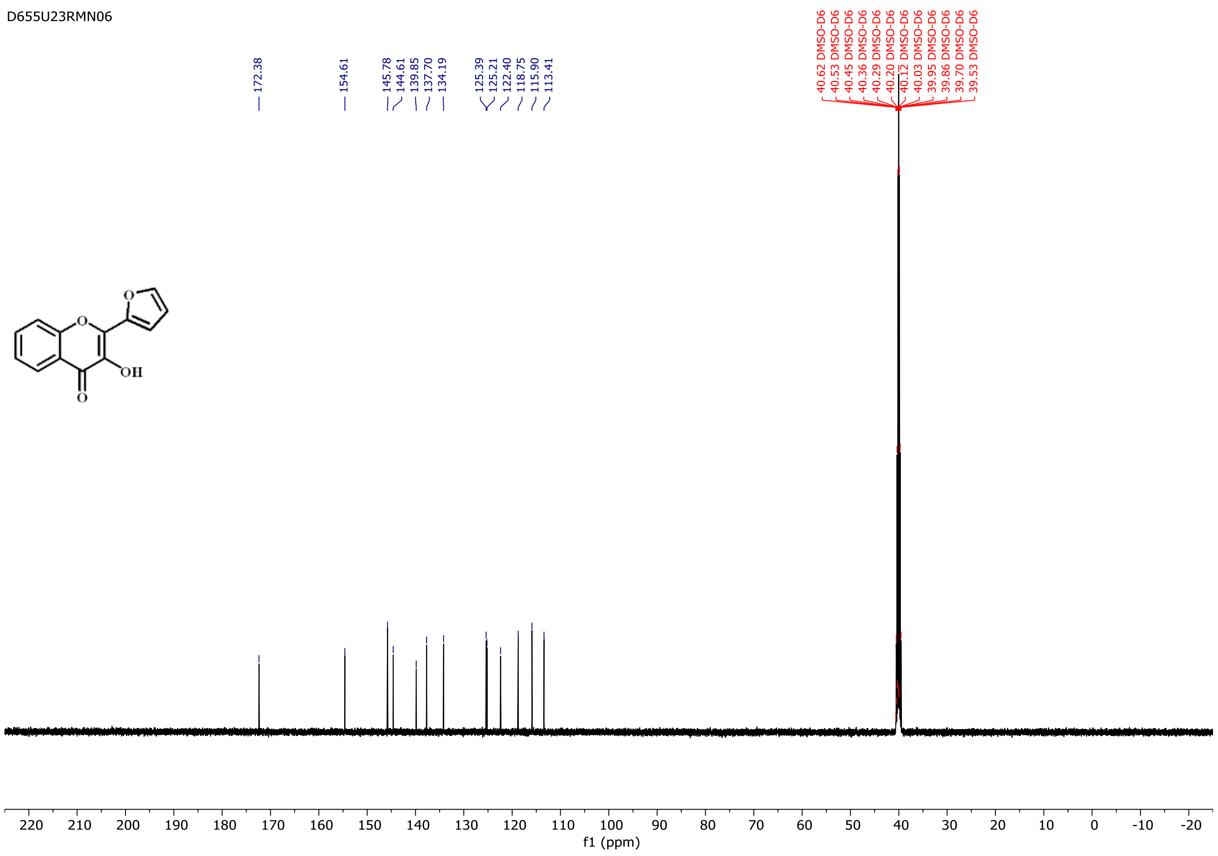

Figure S30. ^{13}C-NMR spectrum of compound **4c**

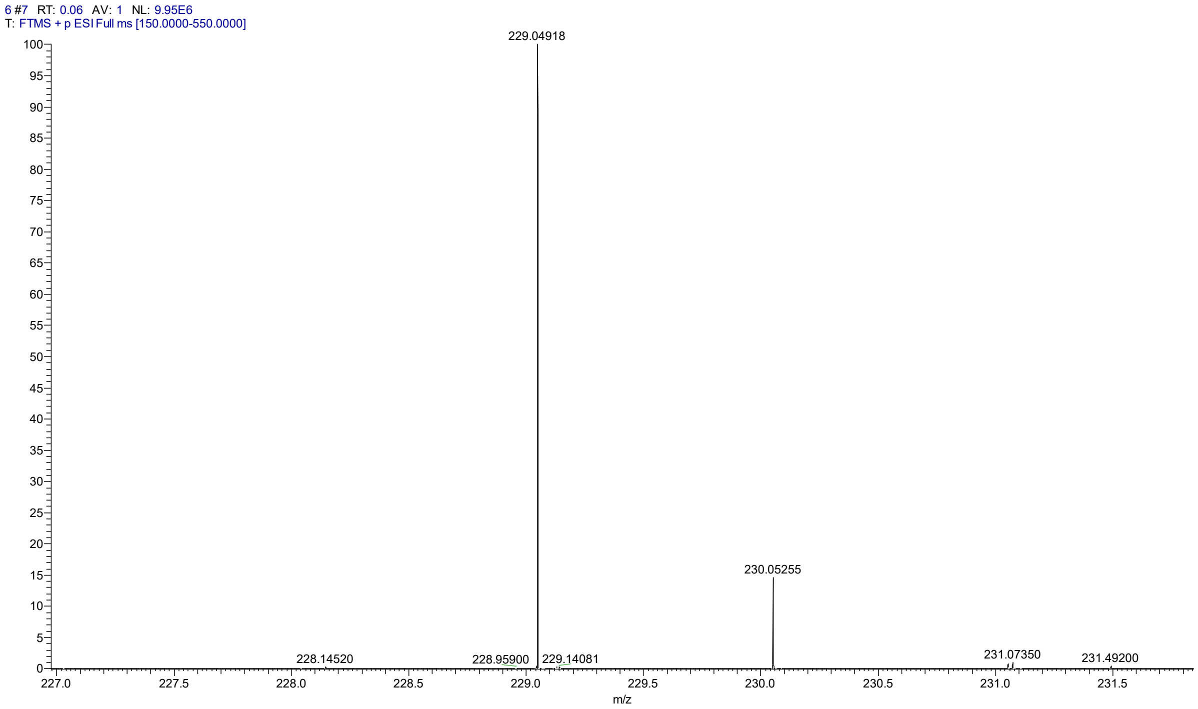

Figure S31. MS spectrum of compound **4c**

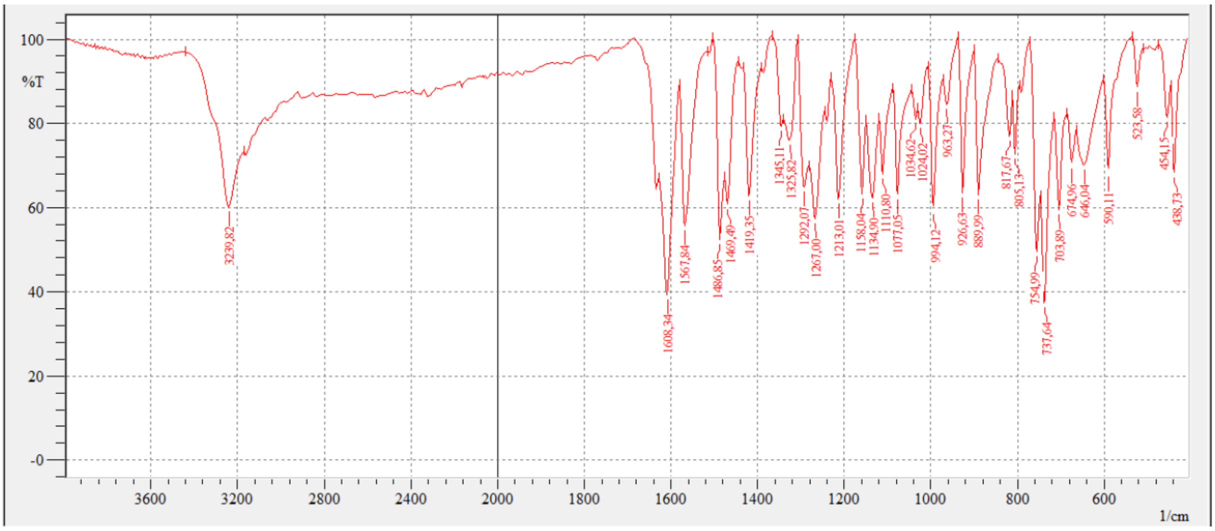

Figure S32. FTIR spectrum of compound **4c**

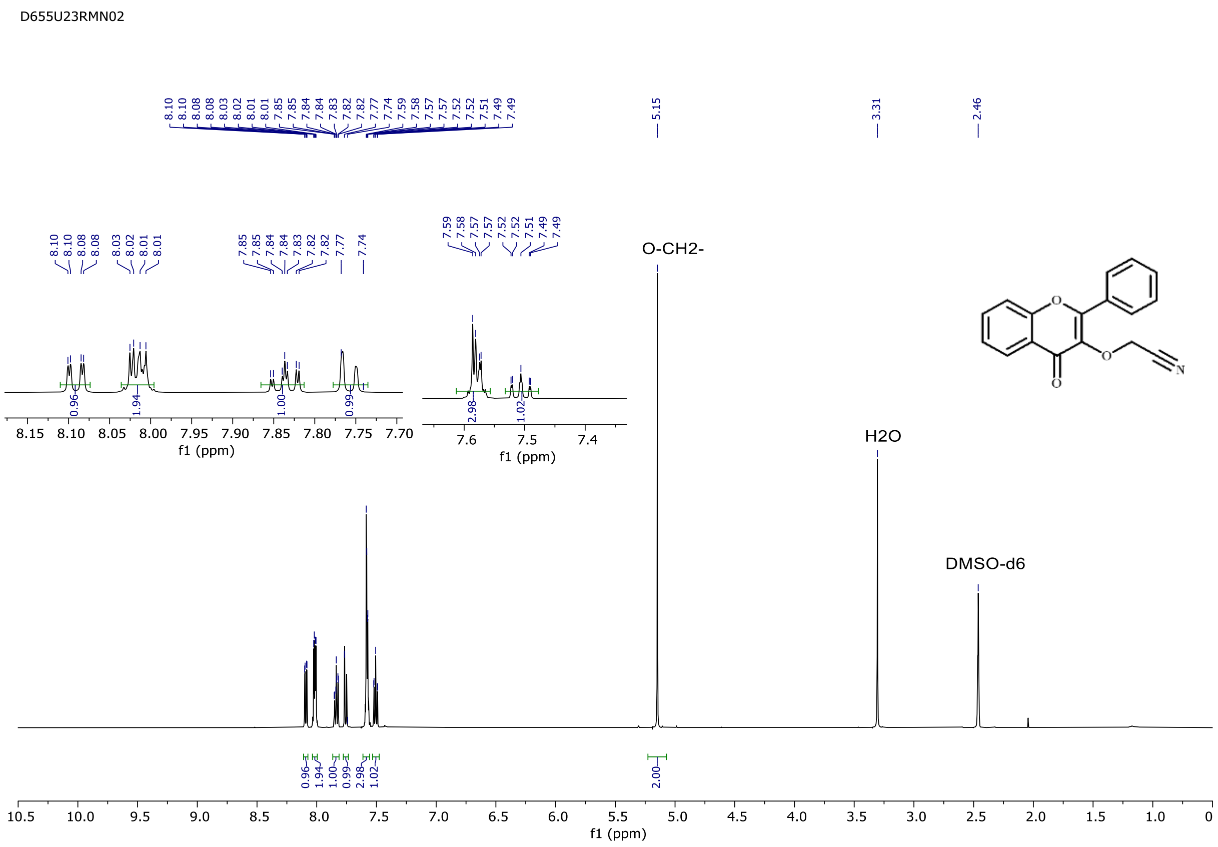

Figure S33. ^{1}H-NMR spectrum of compound **5a**

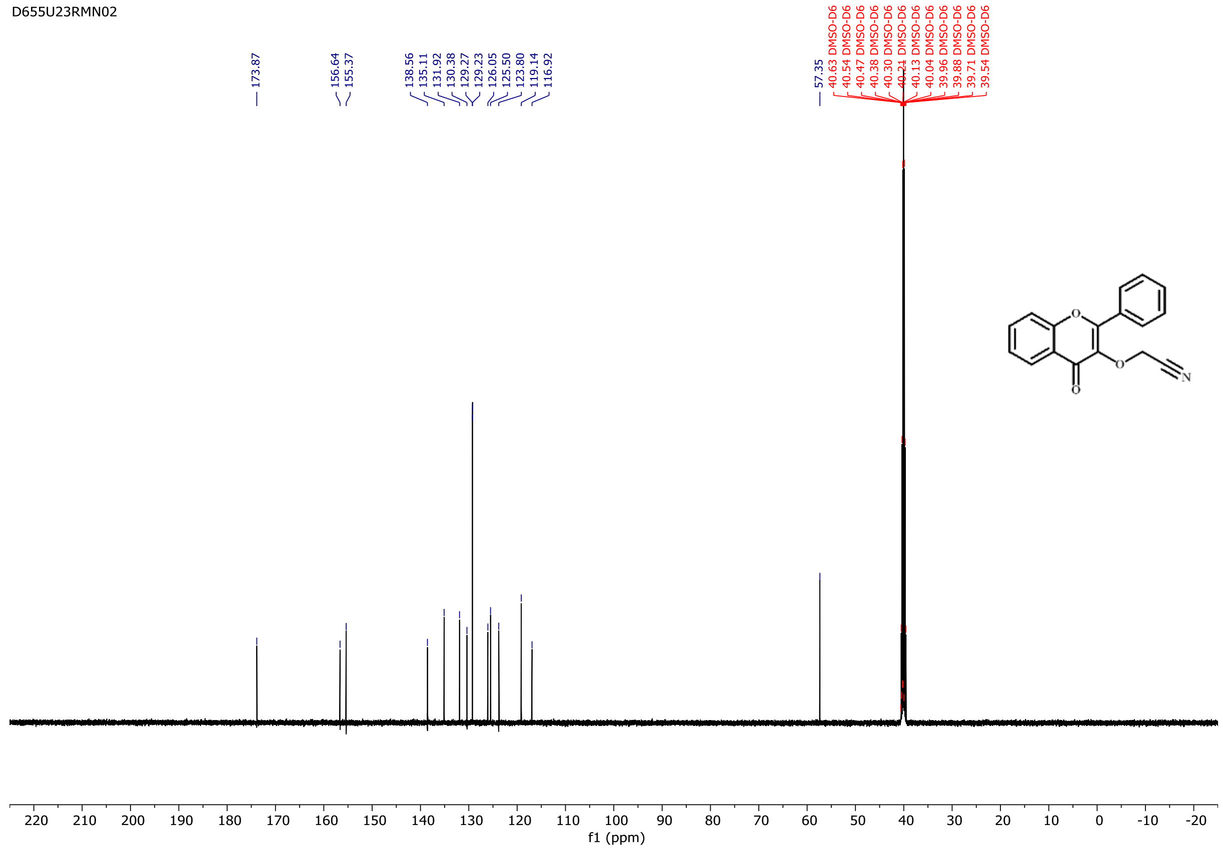

Figure S34. ^{13}C-NMR Spectrum of compound **5a**

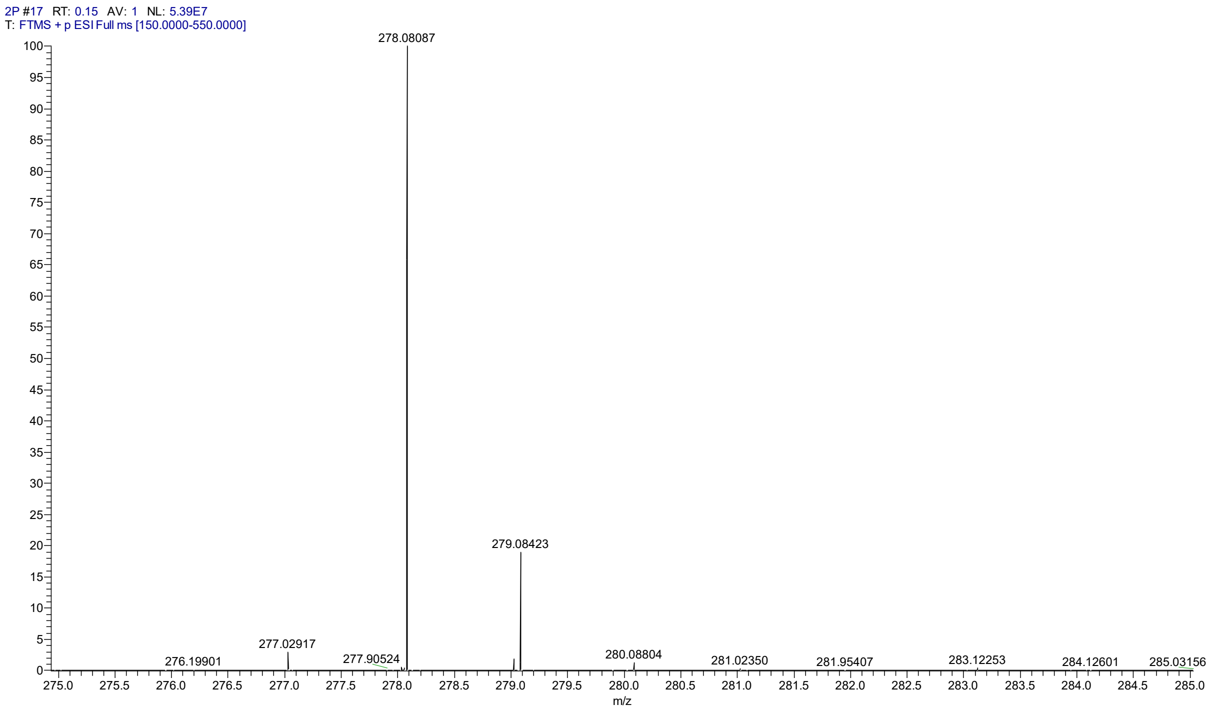

Figure S35. MS spectrum of compound **5a**

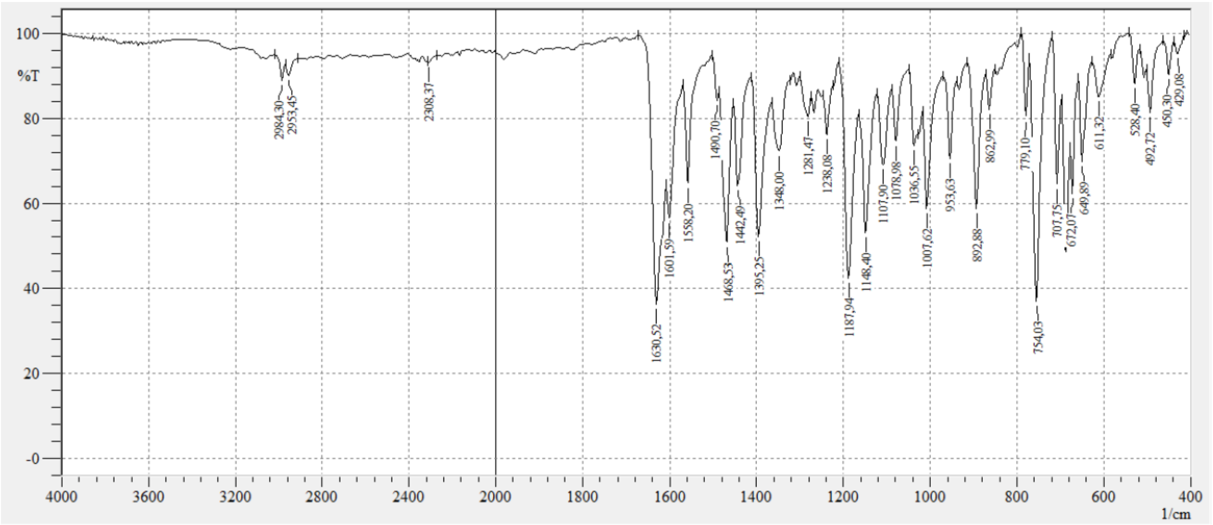

Figure S36. FTIR spectrum of compound **5a**

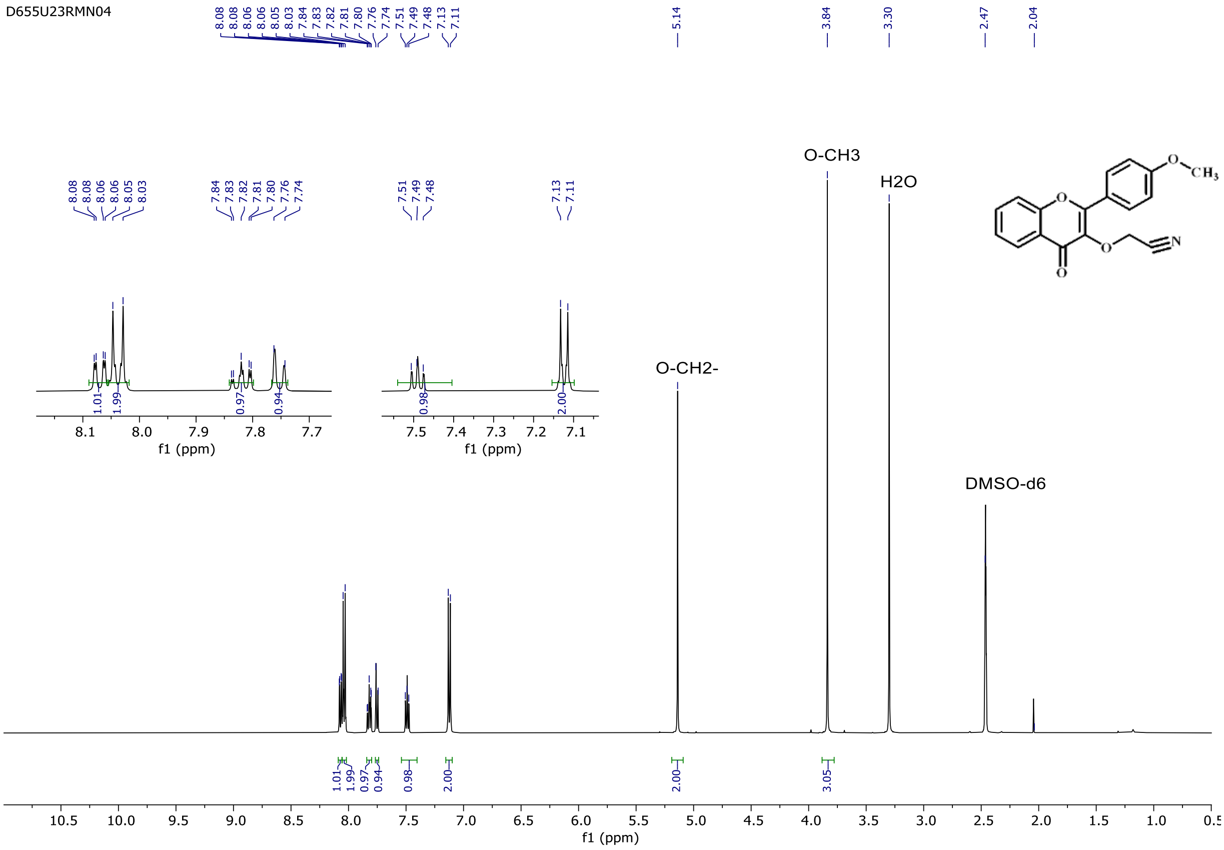

Figure S37. ^{1}H-NMR spectrum of compound **5b**

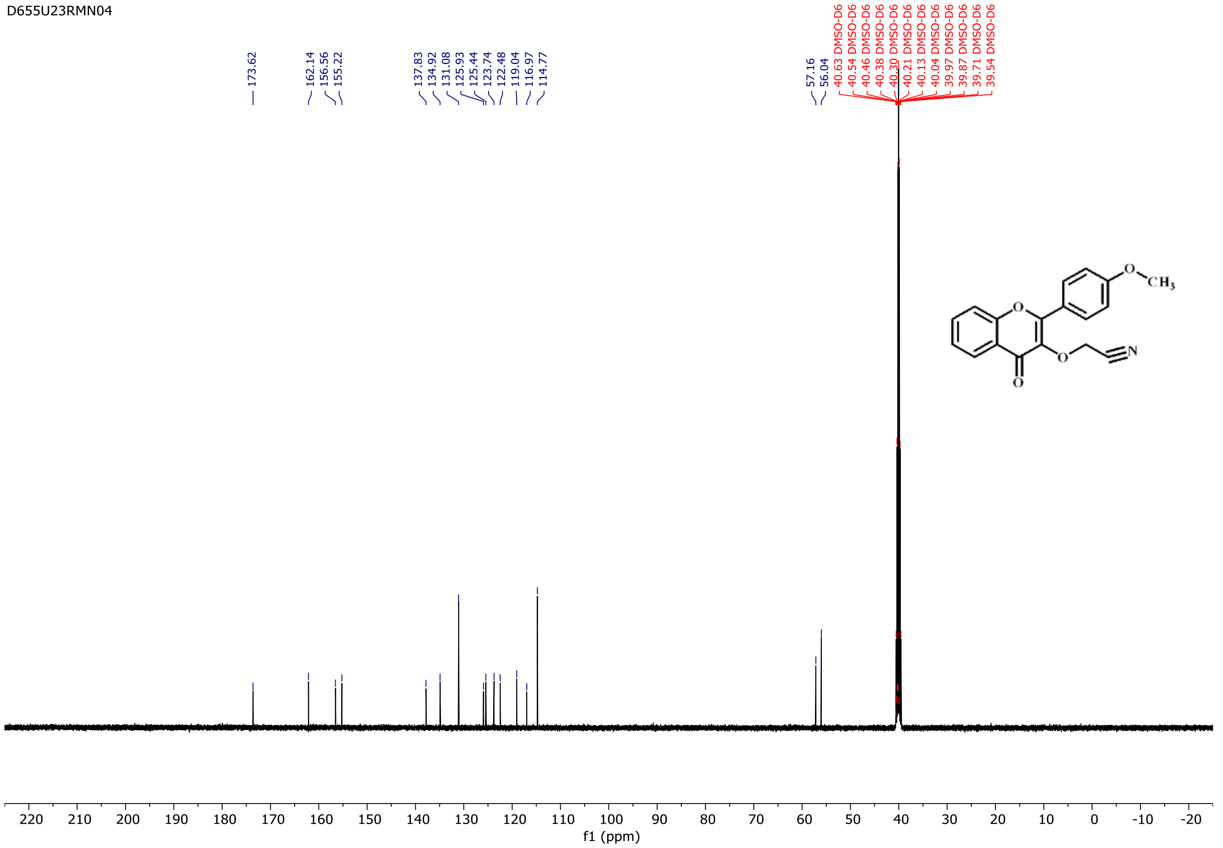

Figure S38. ^{13}C-NMR Spectrum of compound **5b**

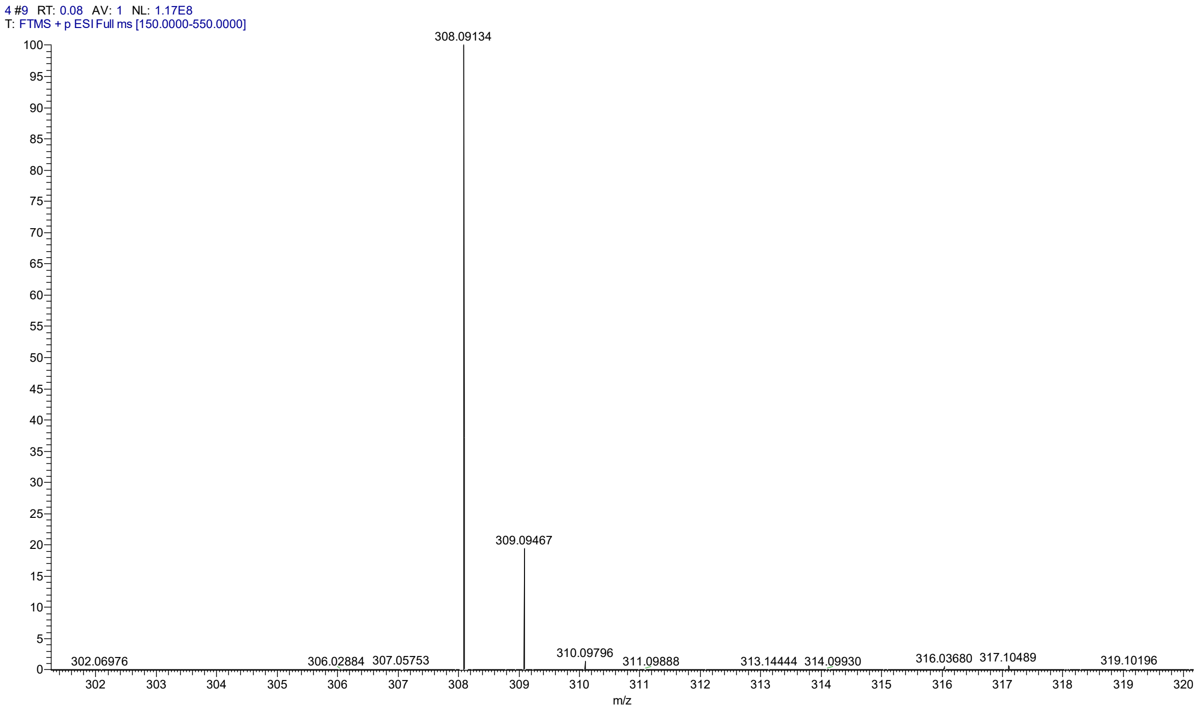

Figure S39. MS spectrum of compound **5b**

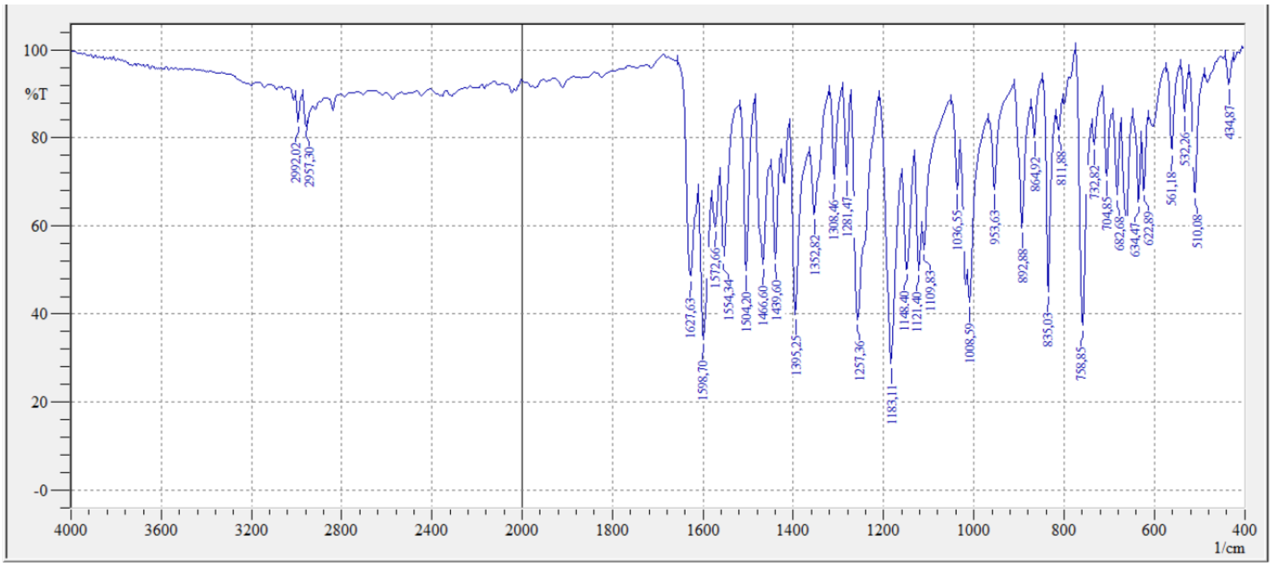

Figure S40. FTIR spectrum of compound **5b**

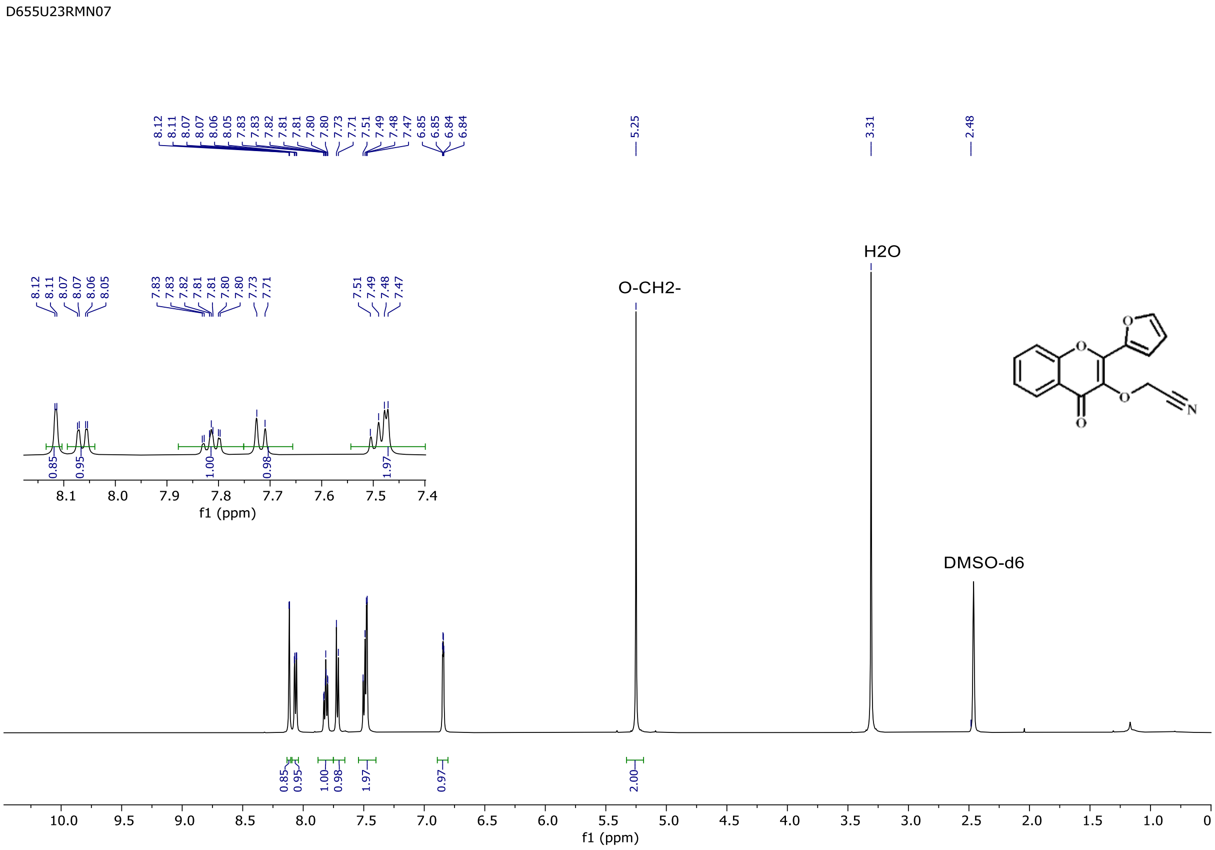

Figure S41. ^{1}H-NMR spectrum of compound **5c**

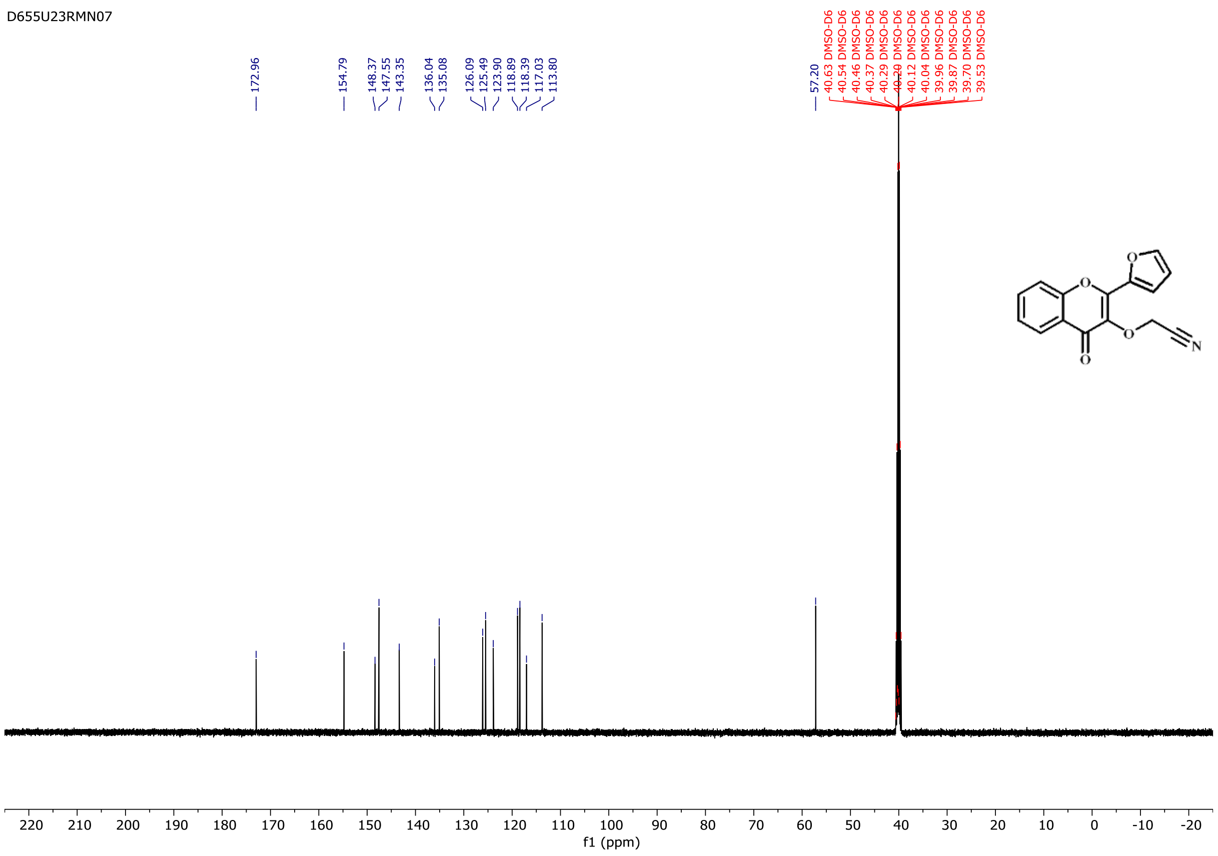

Figure S42. ^{13}C NMR spectrum of compound **5c**

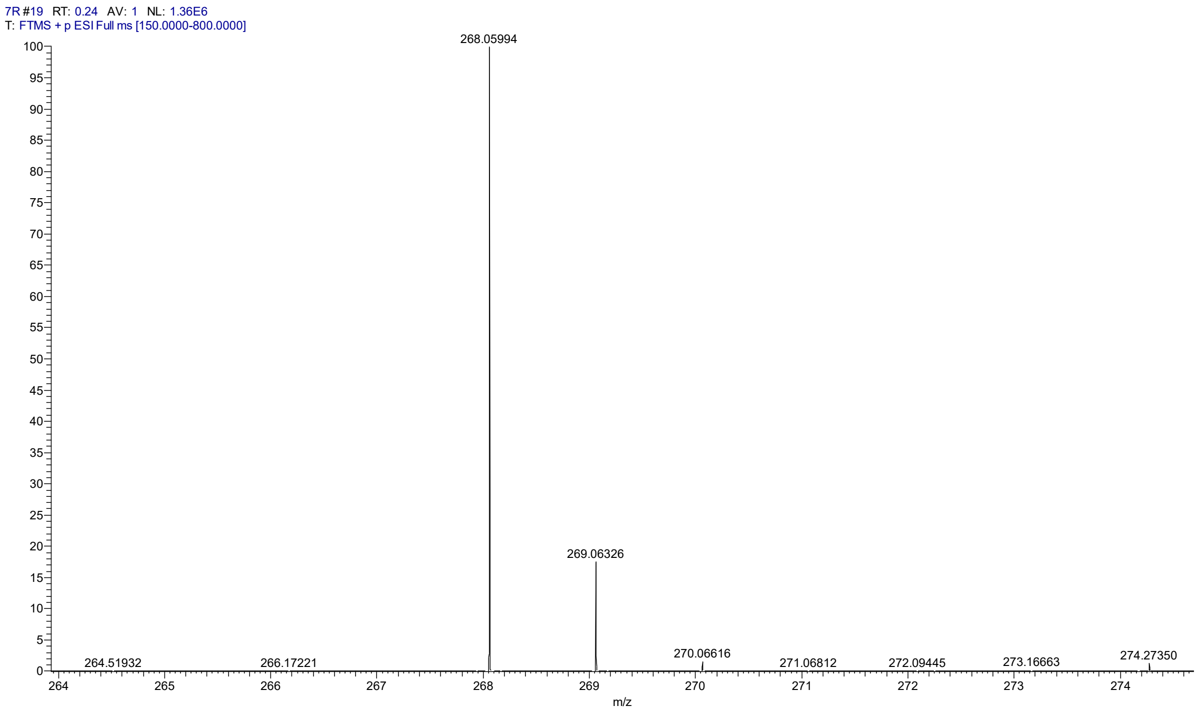

Figure S43. MS spectrum of compound **5c**

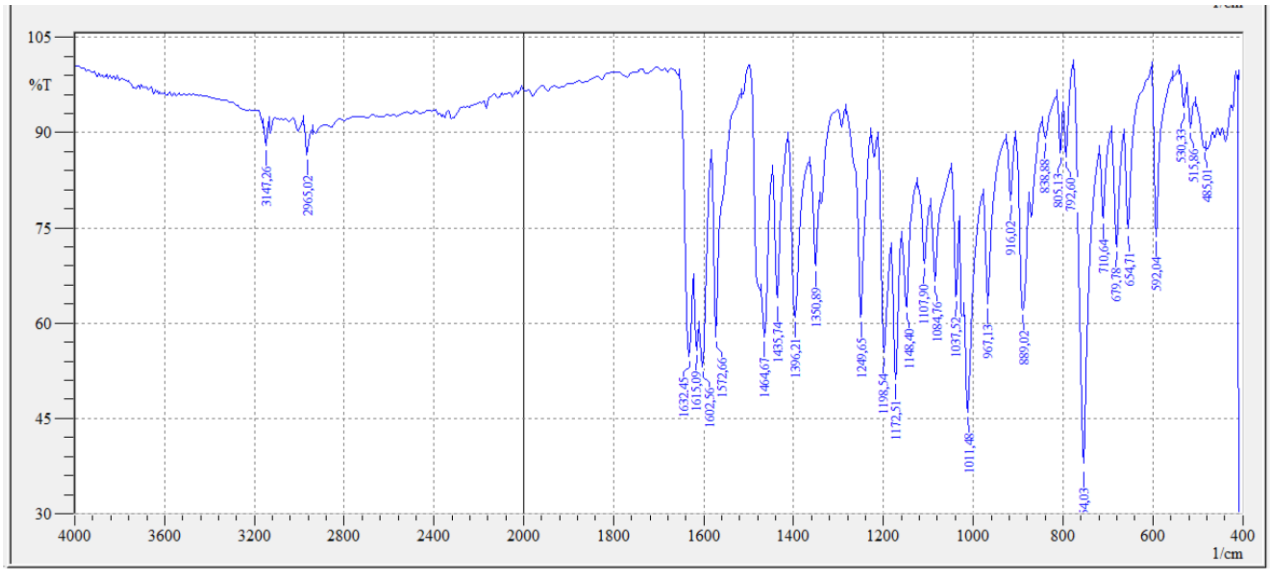

Figure S44. FTIR spectrum of compound **5c**

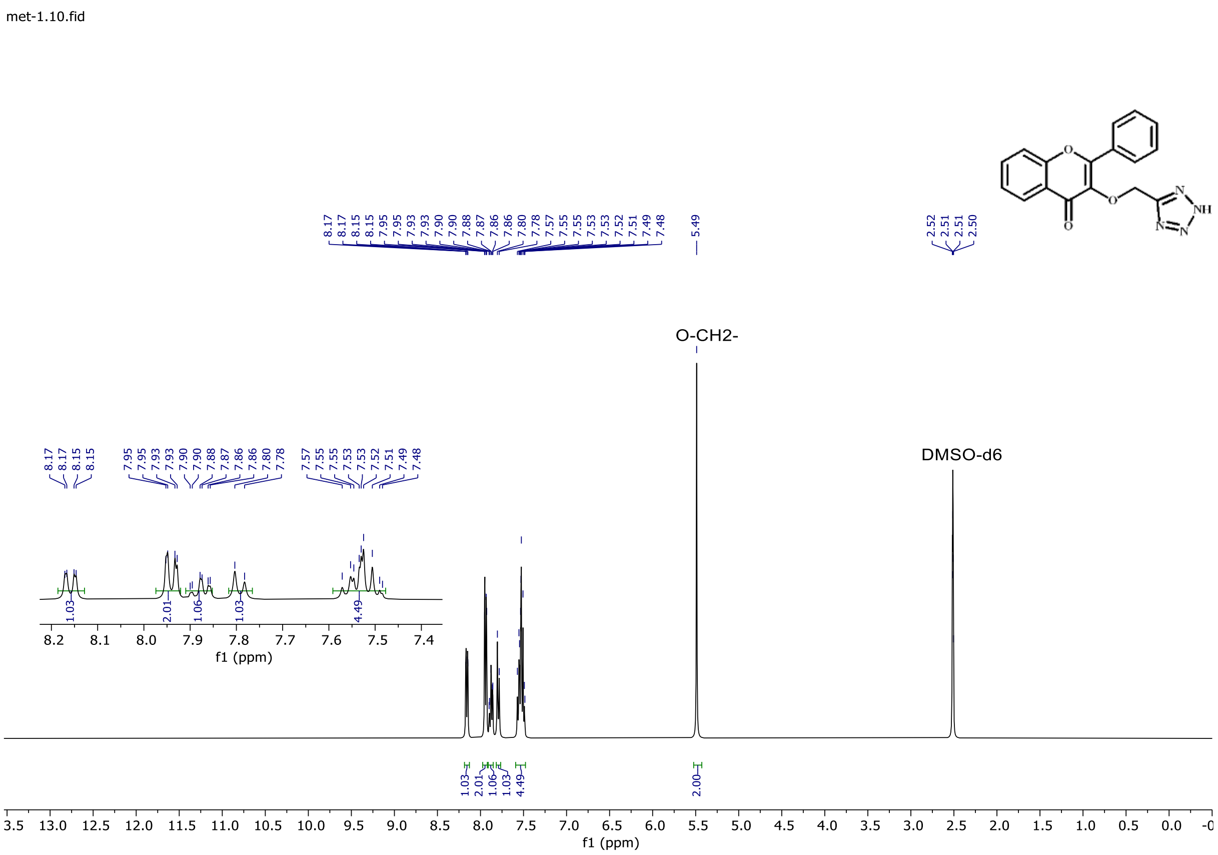

Figure S45. ^{1}H NMR spectrum of compound **6a**

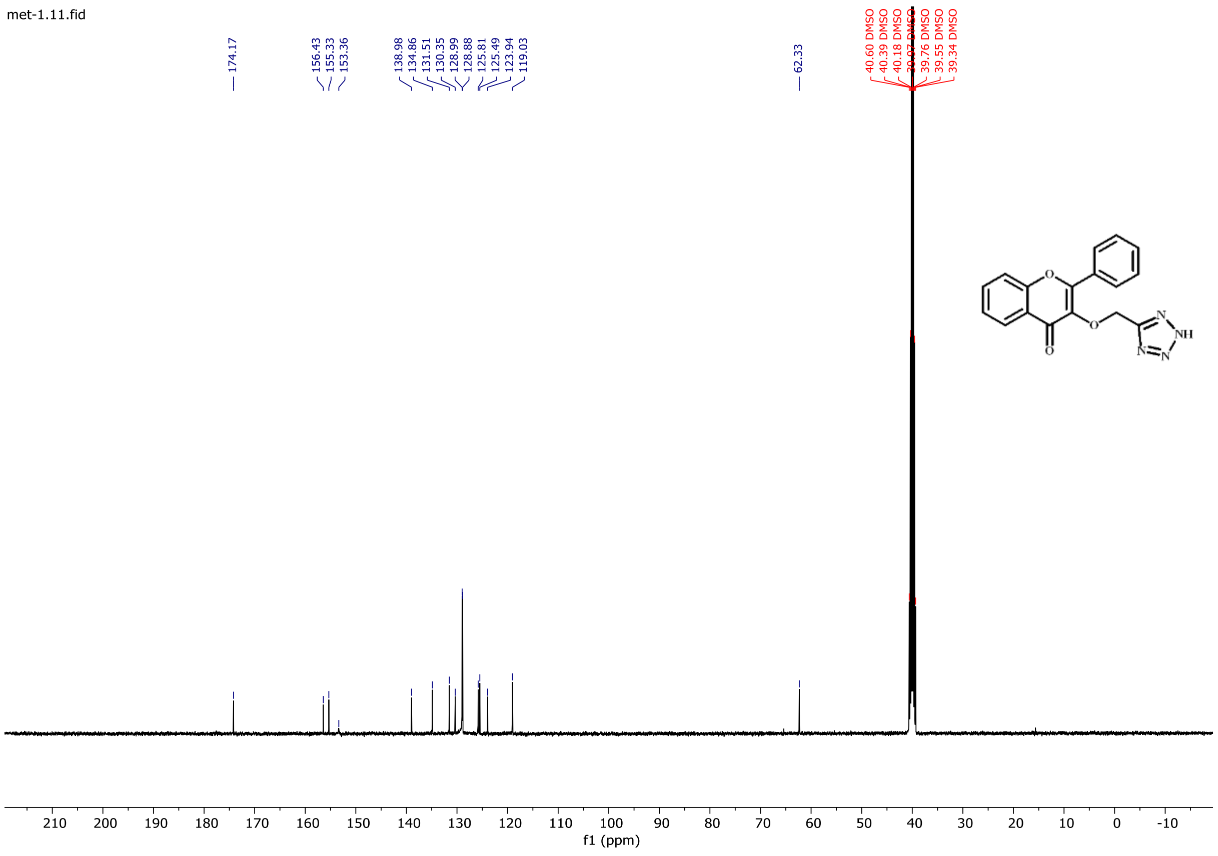

Figure S46. ^{13}C NMR spectrum of compound **6a**

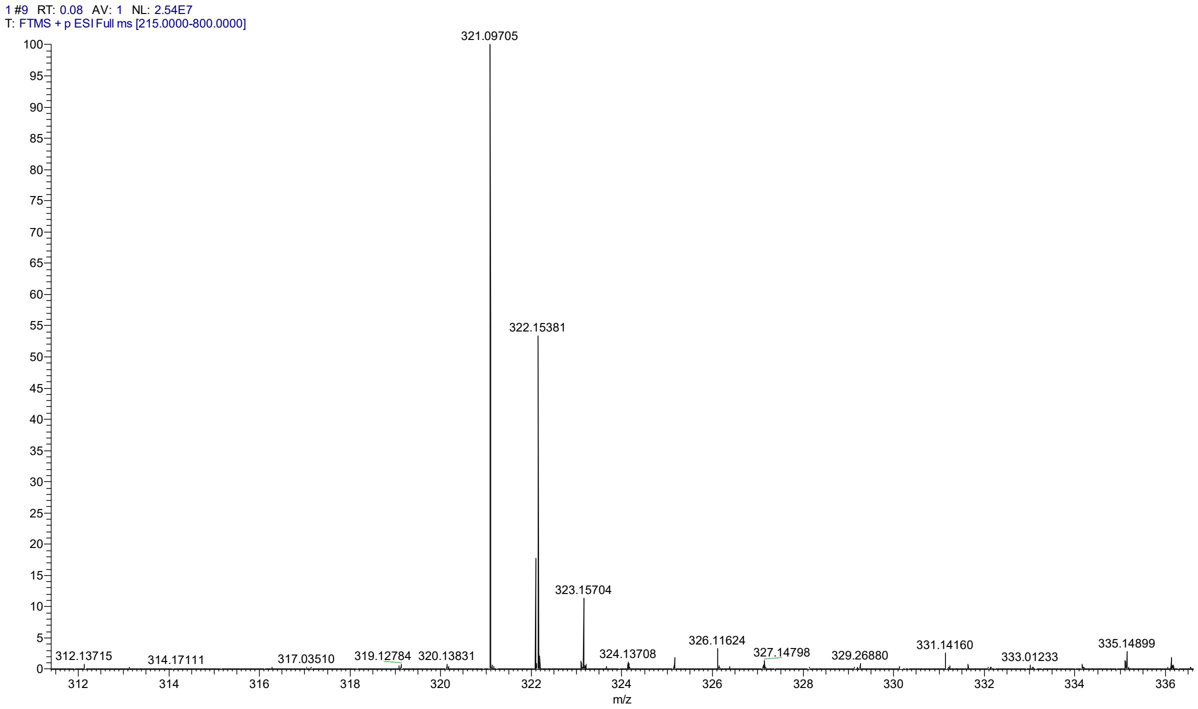

Figure S47. MS spectrum of compound **6a**

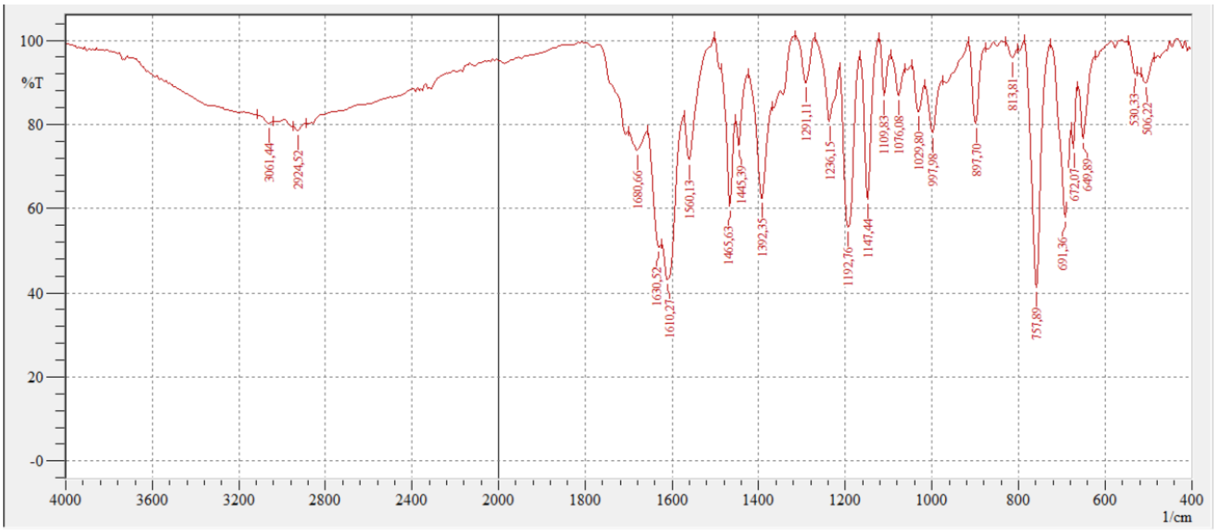

Figure S48. FTIR spectrum of compound **6a**

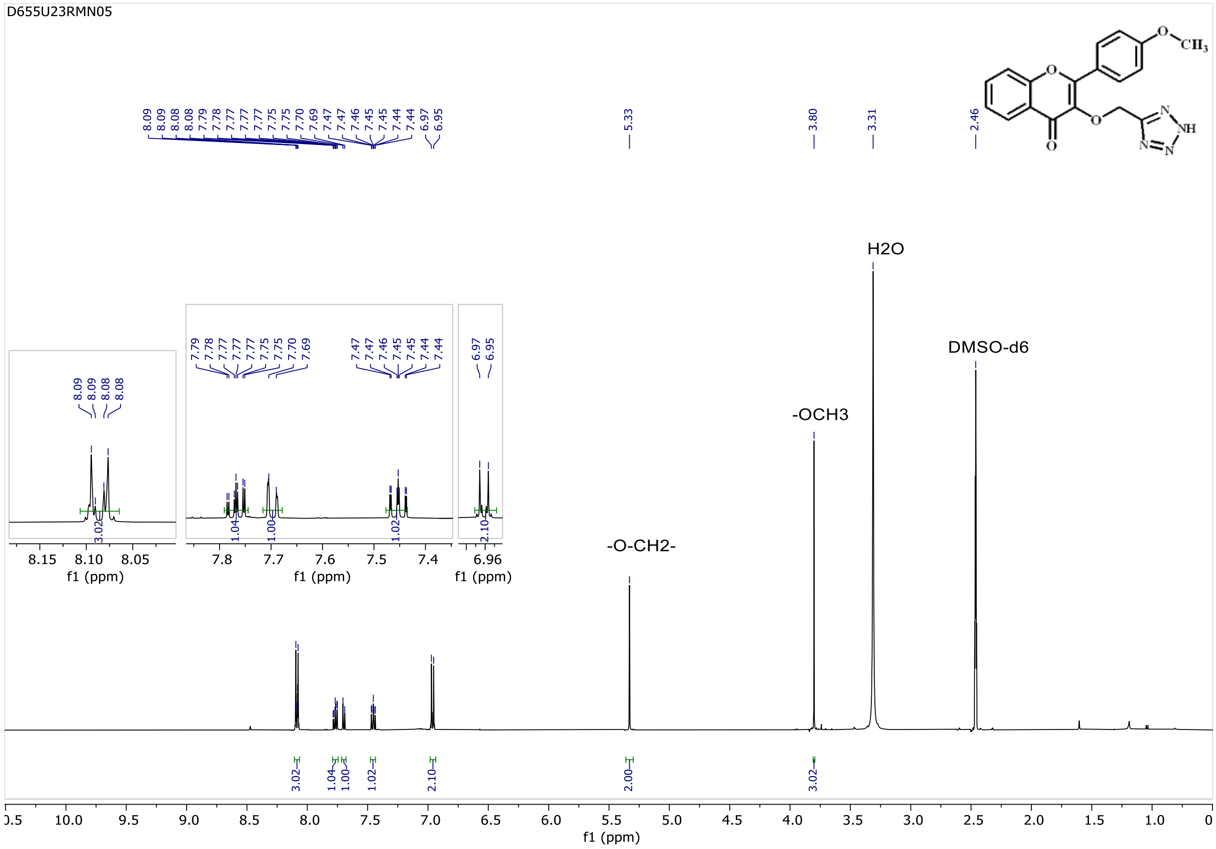

Figure S49. ^{1}H NMR spectrum of compound **6b**

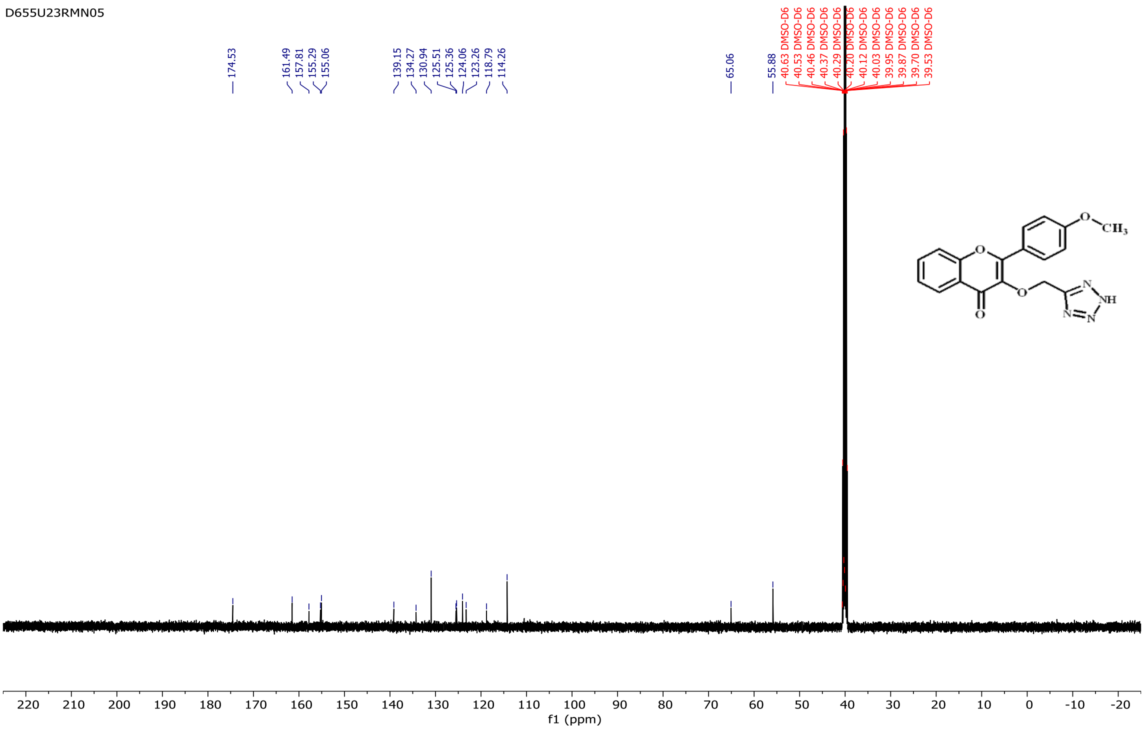

Figure S50. ^{13}C NMR spectrum of compound **6b**

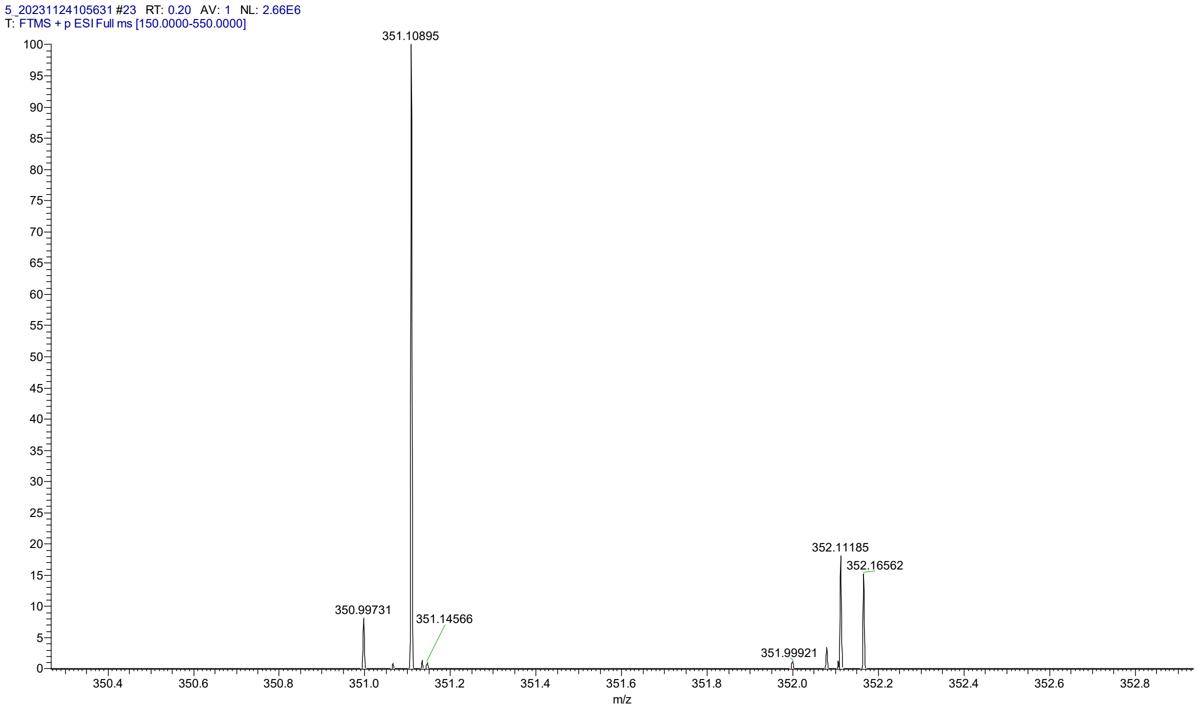

. **Figure S51.** MS spectrum of compound **6b**

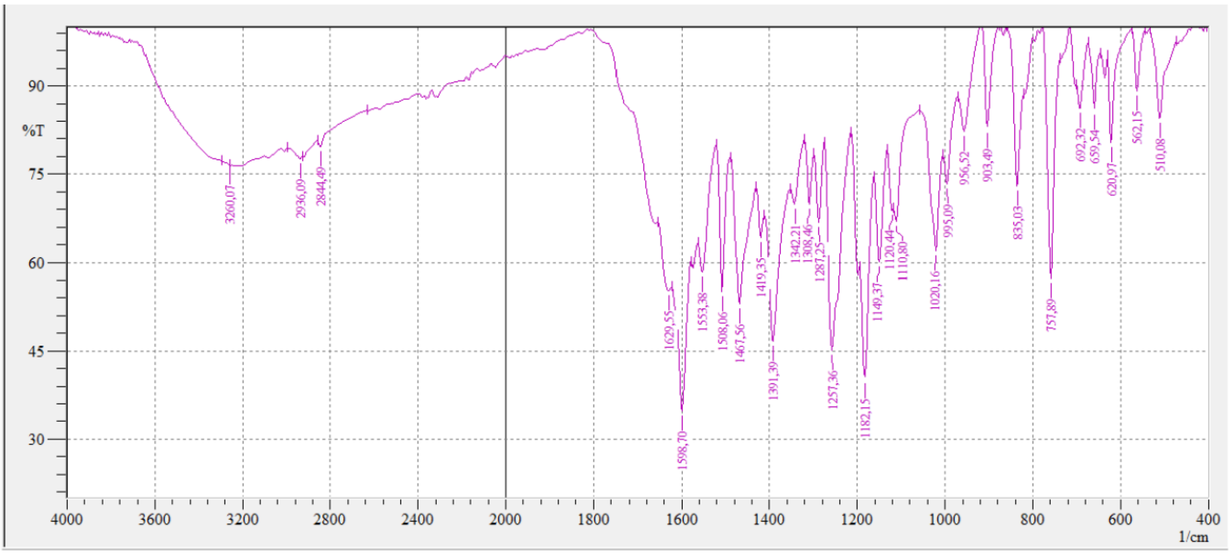

Figure S52. FTIR spectrum of compound **6b**

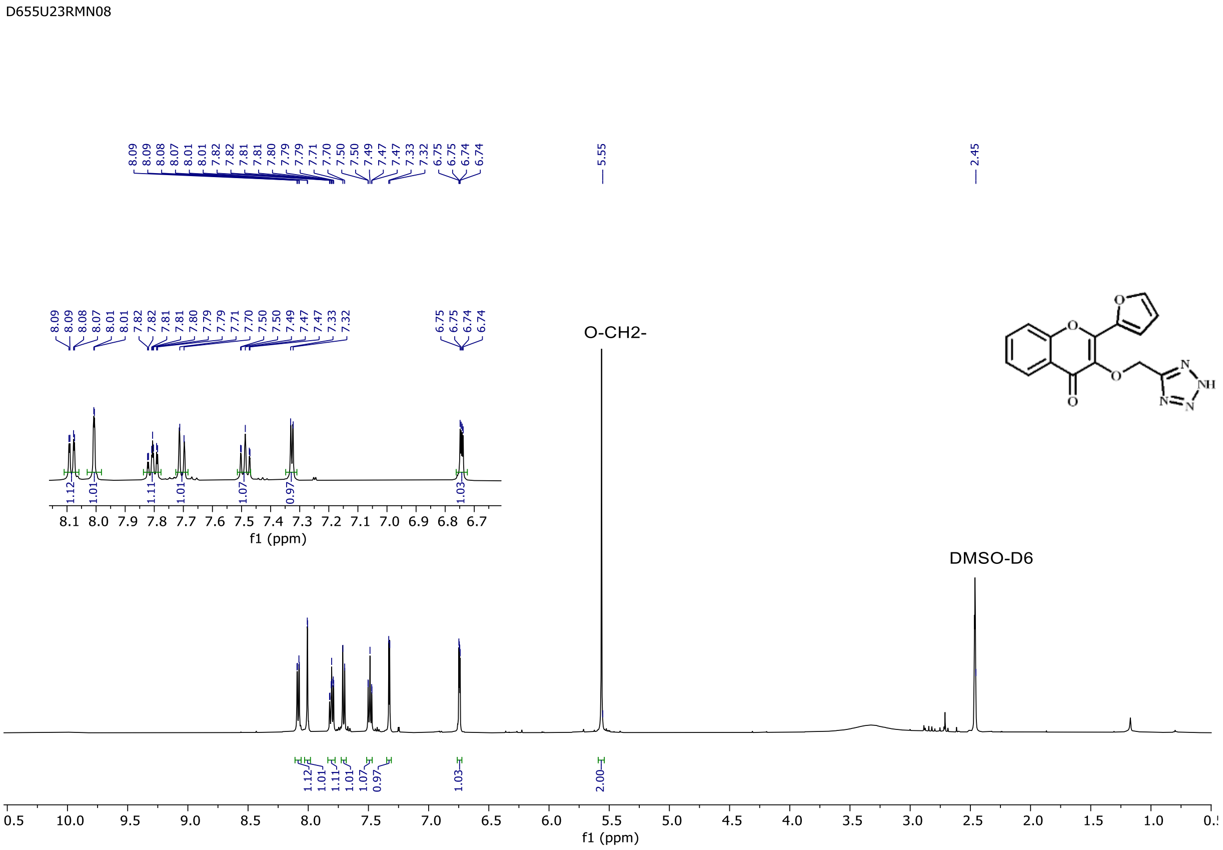

Figure S53. ^{1}H-NMR spectrum of compound **6c**

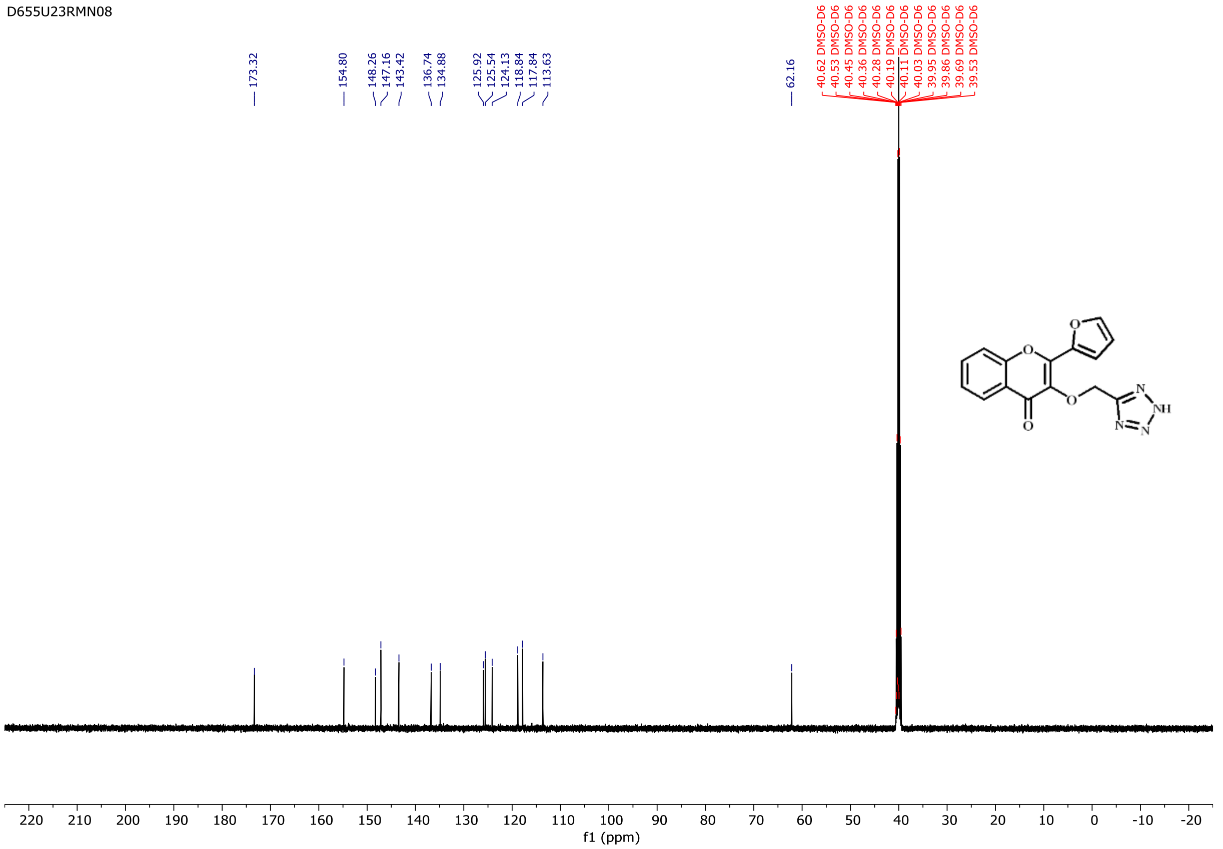

Figure S54. ^{13}C-NMR spectrum of compound **6c**

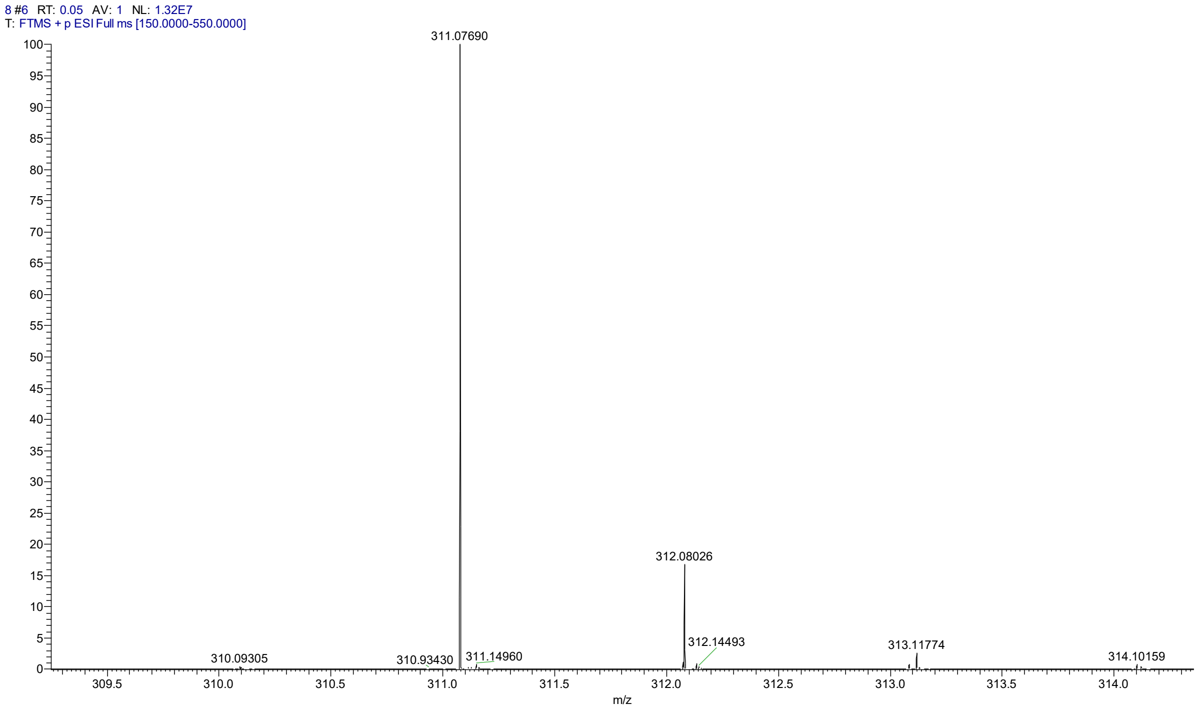

Figure S55. MS spectrum of compound **6c**

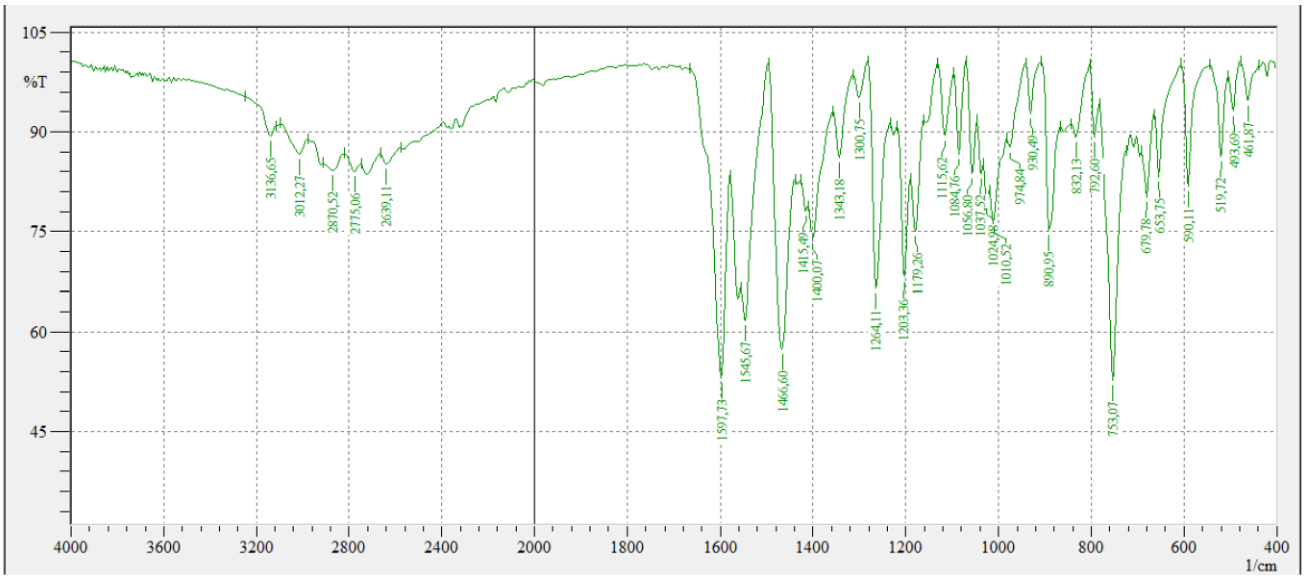

Figure S56. FTIR spectrum of compound **6c**
